# *Piper* Species: A Comprehensive Review on Their Phytochemistry, Biological Activities and Applications

**DOI:** 10.3390/molecules24071364

**Published:** 2019-04-07

**Authors:** Bahare Salehi, Zainul Amiruddin Zakaria, Rabin Gyawali, Salam A. Ibrahim, Jovana Rajkovic, Zabta Khan Shinwari, Tariq Khan, Javad Sharifi-Rad, Adem Ozleyen, Elif Turkdonmez, Marco Valussi, Tugba Boyunegmez Tumer, Lianet Monzote Fidalgo, Miquel Martorell, William N. Setzer

**Affiliations:** 1 Student Research Committee, School of Medicine, Bam University of Medical Sciences, Bam 44340847, Iran; bahar.salehi007@gmail.com; 2 Department of Biomedical Science, Faculty of Medicine and Health Sciences, Universiti Putra Malaysia, 43400 UPM Serdang, Selangor, Malaysia; dr_zaz@yahoo.com; 3 Department of Food and Nutritional Sciences, North Carolina A&T State University, Greensboro, NC 27411, USA; rgyawali@gmail.com (R.G.); ibrah001@ncat.edu (S.A.I.); 4 Institute of Pharmacology, Clinical Pharmacology and Toxicology, Medical Faculty, University of Belgrade, 11129 Belgrade, Serbia; jolarajkovic@yahoo.com; 5 Department of Biotechnology, Quaid-i-Azam University, Islamabad 45320, Pakistan; shinwari2008@gmail.com (Z.K.S.); tariqkhanbio@gmail.com (T.K.); 6 Food Safety Research Center (salt), Semnan University of Medical Sciences, Semnan 35198-99951, Iran; 7 Graduate Program of Biomolecular Sciences, Institute of Natural and Applied Sciences, Canakkale Onsekiz Mart University, 17020 Canakkale, Turkey; ademozleyen@gmail.com (A.O.); elifturkdonmez@stu.comu.edu.tr (E.T.); 8 European Herbal and Traditional Medicine Practitioners Association (EHTPA), 25 Lincoln Close, Tewkesbury GL20 5TY, UK; 9 Department of Molecular Biology and Genetics, Faculty of Arts and Science, Canakkale Onsekiz Mart University, 17020 Canakkale, Turkey; 10 Parasitology Department, Institute of Tropical Medicine “Pedro Kouri”, Havana 10400, Cuba; monzote@ipk.sld.cu; 11 Department of Nutrition and Dietetics, Faculty of Pharmacy, University of Concepcion, 4070386 Concepcion, VIII-Bio Bio Region, Chile; 12 Department of Chemistry, University of Alabama in Huntsville, Huntsville, AL 35899, USA; 13 Aromatic Plant Research Center, 230 N 1200 E, Suite 100, Lehi, UT 84043, USA

**Keywords:** pepper, essential oil, antimicrobial, traditional medicine, anticancer, anti-inflammatory

## Abstract

*Piper* species are aromatic plants used as spices in the kitchen, but their secondary metabolites have also shown biological effects on human health. These plants are rich in essential oils, which can be found in their fruits, seeds, leaves, branches, roots and stems. Some *Piper* species have simple chemical profiles, while others, such as *Piper nigrum*, *Piper betle*, and *Piper auritum*, contain very diverse suites of secondary metabolites. In traditional medicine, *Piper* species have been used worldwide to treat several diseases such as urological problems, skin, liver and stomach ailments, for wound healing, and as antipyretic and anti-inflammatory agents. In addition, *Piper* species could be used as natural antioxidants and antimicrobial agents in food preservation. The phytochemicals and essential oils of *Piper* species have shown strong antioxidant activity, in comparison with synthetic antioxidants, and demonstrated antibacterial and antifungal activities against human pathogens. Moreover, *Piper* species possess therapeutic and preventive potential against several chronic disorders. Among the functional properties of *Piper* plants/extracts/active components the antiproliferative, anti-inflammatory, and neuropharmacological activities of the extracts and extract-derived bioactive constituents are thought to be key effects for the protection against chronic conditions, based on preclinical in vitro and in vivo studies, besides clinical studies. Habitats and cultivation of *Piper* species are also covered in this review. In this current work, available literature of chemical constituents of the essential oils *Piper* plants, their use in traditional medicine, their applications as a food preservative, their antiparasitic activities and other important biological activities are reviewed.

## Table of Contents


Introduction ...........................................................................................................................................6Habitat and cultivation of *Piper* plants ..............................................................................................7
2.1.Habitat of *Piper nigrum* L. .............................................................................................................72.2.Cultivation of *Piper nigrum* L. ......................................................................................................8Chemical constituents of the essential oils of *Piper* species .............................................................9
3.1.*Piper aduncum* L. ..........................................................................................................................113.2.*Piper amalago* L. ............................................................................................................................153.3.*Piper betle* L. ..................................................................................................................................163.4.*Piper cubeba* Bojer .........................................................................................................................183.5.*Piper nigrum* L. .............................................................................................................................203.6.*Piper longum* L. .............................................................................................................................243.7.*Piper arboreum* Aubl. ....................................................................................................................243.8.*Piper auritum* Kunth ....................................................................................................................263.9.*Piper cernuum* Vell. ......................................................................................................................263.10.*Piper dilatatum* Rich. ..................................................................................................................283.11.*Piper gaudichaudianum* Kunth ...................................................................................................293.12.*Piper hispidum* Sw. (including references to the synonym *Piper hispidinervum* C.DC.) ....313.13.*Piper guineense* Schumach. & Thonn .......................................................................................343.14.*Piper marginatum* Jacq. ...............................................................................................................343.15.*Piper umbellatum* L. ....................................................................................................................363.16.*Piper tuberculatum* Jacq. .............................................................................................................363.17.Other *Piper* species ....................................................................................................................39Traditional uses of *Piper* species .......................................................................................................55
4.1.*Piper abbreviatum *Opiz ................................................................................................................ 554.2.*Piper aduncum *L. .......................................................................................................................... 554.3.*Piper boehmeriifolium *(Wall. ex Miq.) C.DC. ............................................................................. 564.4.*Piper sylvaticum *Roxb. ................................................................................................................. 564.5.*Piper capense *L.f. ........................................................................................................................... 564.6.*Piper cubeba *L. ............................................................................................................................... 564.7.*Piper gibbilimbum *C.DC. .............................................................................................................. 564.8.*Piper guineense *Schum and Thonn ............................................................................................. 564.9.*Piper longum *L. (syn. P. *latifolium *Forst.; P. *chaba *Hunter) ..................................................... 574.10.*Piper nigrum *L. ........................................................................................................................... 574.11.*Piper cavalcantei *Yunck. ............................................................................................................. 584.12.*Piper marginatum *Jacq................................................................................................................ 584.13.*Piper umbellatum *L. .................................................................................................................... 584.14.*Piper aborescens *Roxb. ................................................................................................................ 594.15.*Piper acutifolium *Ruiz and Pav. ................................................................................................ 594.16.*Piper alatabaccum *Trel. & Yunck ............................................................................................... 594.17.*Piper angustifolium *Lam. ........................................................................................................... 594.18.*Piper auritum *Kunth .................................................................................................................. 594.19.*Piper barbatum *Kunth ................................................................................................................ 604.20.*Piper betle *L. ................................................................................................................................ 604.21.*Piper claussenianum *(Miq.) C. DC. ............................................................................................ 604.22.*Piper cumanense *Kunth .............................................................................................................. 604.23.*Piper dennisii *Trel. ...................................................................................................................... 614.24.*Piper fimbriulatum *C. DC. .......................................................................................................... 614.25.*Piper glabratum *Kunth ............................................................................................................... 614.26.*Piper grande *Vahl........................................................................................................................ 614.27.*Piper hayneanum *C.DC. ............................................................................................................. 614.28.*Piper hispidum *L. ........................................................................................................................ 614.29.*Piper holtonii *C.DC. .................................................................................................................... 614.30.*Piper jacquemontianum *Kunth ................................................................................................... 614.31.*Piper jericoense *Trel. & Yunck ................................................................................................... 624.32.*Piper lanceaefolium *HBK. ........................................................................................................... 624.33.*Piper methysticum *G.Forst ......................................................................................................... 624.34.*Piper multiplinervium *C.DC. ..................................................................................................... 624.35.*Piper obrutum *Trel. & Yunck. ................................................................................................... 624.36.*Piper ovatum *Vahl ...................................................................................................................... 624.37.*Piper pulchrum *C.DC. ................................................................................................................ 624.38.*Piper pyrifolium *Vahl. ................................................................................................................. 624.39.*Piper regnellii *(Miq.) C. DC. ...................................................................................................... 634.40.*Piper retrofractum *Vahl .............................................................................................................. 634.41.*Piper sanvicentense *Trel. & Yunck. ........................................................................................... 634.42.*Piper sarmentosum *Roxb. ........................................................................................................... 634.43.*Piper sintenense *Hatus. .............................................................................................................. 634.44.*Piper strigosum *Trel. & Yunck. ................................................................................................. 644.45.*Piper stylosum *Miq. .................................................................................................................... 644.46.*Piper tuberculatum *Jacq. ............................................................................................................. 644.47.*Piper xanthostachyum *C. DC ...................................................................................................... 644.48.*Piper carpunya *Ruiz & Pav (syn: *P. lenticellosum *C.D.C.) ...................................................... 644.49.*Piper obliquum *Ruiz & Pavon ................................................................................................... 644.50.*Piper laetispicum *C. DC .............................................................................................................. 644.51.*Piper arboreum *Aubl. .................................................................................................................. 644.52.*Piper amalago *L. .......................................................................................................................... 654.53.*Piper ribesioides *Wall. (syn: *P. sumatranum *(Miq.) C. DC.) .................................................... 654.54.*Piper corcovadensis *(Miq.) C. DC. ............................................................................................. 654.55.*Piper futokadsura *Siebold ........................................................................................................... 654.56.*Piper elongatum *Vahl. ................................................................................................................. 654.57.*Piper mikanianum *(Kunth) Steud .............................................................................................. 654.58.*Piper medium *Jacq. ...................................................................................................................... 664.59.*Piper wallichii *(Miq.) Hand.-Mazz. .......................................................................................... 664.60.*Piper truncatum *Vell................................................................................................................... 664.61.*Piper aequale *Vahl ....................................................................................................................... 664.62.*Piper alyreanum *C.DC ................................................................................................................ 664.63.*Piper attenuatum *Buch.-Ham. ex Miq. ..................................................................................... 664.64.*Piper augustum *Rudge ............................................................................................................... 664.65.*Piper darienense *C.DC. ............................................................................................................... 664.66.*Piper reticulatum *L. ..................................................................................................................... 664.67.*Piper hongkongense *C. DC .......................................................................................................... 664.68.*Piper kadsura *(Choisy) Ohwi .................................................................................................... 674.69.*Piper macropodum *C. DC ........................................................................................................... 674.70.*Piper mutabile *C. DC .................................................................................................................. 674.71.*Piper puberulilimbum *C. DC ...................................................................................................... 674.72.*Piper yunnanense *Tseng ............................................................................................................. 674.73.*Piper callosum *Ruiz & Pav. ........................................................................................................ 674.74.*Piper conejoense *Trel. & Yunck. ................................................................................................. 674.75.*Piper novae-hollandiae *Miq. ........................................................................................................ 674.76.*Piper mullesua *Buch.-Ham. ex D. Don ..................................................................................... 674.77.*Piper peltatum *L. ......................................................................................................................... 674.78.*Piper interruptum *Opiz (syn: P. ribesoides Wall.) .................................................................. 684.79.*Piper guianense *(Klotzsch) C.DC. ............................................................................................. 684.80.*Piper fragile *Benth. ..................................................................................................................... 684.81.*Piper coruscans *Kunth. ............................................................................................................... 684.82.*Piper caninum *Blume ................................................................................................................. 684.83.*Piper bantamese *Blume ............................................................................................................... 684.84.*Piper sanctum *(Miq.) Schltdl. .................................................................................................... 684.85.*Piper sylvestre *Lam. .................................................................................................................... 694.86.*Piper lanatum *Roxb. ................................................................................................................... 694.87.*Piper porphyrophyllum *N.E.Br. .................................................................................................. 694.88.*Piper cernuum *Vell. .................................................................................................................... 694.89.*Piper cordulatum *C. DC. ............................................................................................................. 694.90.*Piper divaricatum *Meyer ............................................................................................................ 694.91.*Piper flaviflorum *C. DC. ............................................................................................................. 694.92.*Piper gaudichaudianum *(Kunth) Kunth ex Steud .................................................................... 694.93.*Piper hainanense *Hemsl. ............................................................................................................ 694.94.*Piper klotzschianum *Kunth. ....................................................................................................... 694.95.*Piper miniatum *Blume ................................................................................................................ 694.96.*Piper *aff. *pedicellatum *C. DC. .................................................................................................... 704.97.*Piper philippinum *Miq. (syn. *P. kwashoense *Hayata) .............................................................. 704.98.*Piper piscatorum *Trel. & Yunck................................................................................................. 704.99.*Piper ossanum *Trel. ..................................................................................................................... 704.100.*Piper semiimmersum *C. DC. ..................................................................................................... 704.101.*Piper submultinerve *C. DC. ...................................................................................................... 704.102.*Piper loretoanum *Trel. ............................................................................................................... 704.103.*Piper mediocre *C.DC. ................................................................................................................ 704.104.*Piper sanguineispicum *Trel. ...................................................................................................... 704.105.*Piper taiwanense *Lin & Lu ....................................................................................................... 704.106.Piper *trichostachyon *(Miq.) C. DC. ......................................................................................... 71Food preservative of *Piper* Plants ......................................................................................................71
5.1.Antioxidative Activity ................................................................................................................715.2.Antimicrobial Activity ................................................................................................................72Antiparasitic activities of *Piper* Species ............................................................................................75Biological activities *Piper* Plants ........................................................................................................77
7.1.Antiproliferative/Anti-cancer Properties .................................................................................777.2.Anti-inflammatory Properties ...................................................................................................817.3.Neuropharmacological Activities .............................................................................................837.4.Clinical Studies ............................................................................................................................87Conclusions and Future Perspectives ..............................................................................................96


## 1. Introduction

In these modern times, the concept of a return to the “roots” of medicine is starting to become more and more popular. Scientific progress has provided new approaches for the analysis of different folk herbs that are used in various cultures [1,2]. The pharmacological properties of plants used as food, medicine or for spiritual purposes during the centuries have been confirmed through new approaches to their analyses [3,4,5]. The heritage of using some plants in traditional medicine is continuously being corroborated in terms of their effects through scientific inquiry [6,7]. One of the widely distributed plant genera in pantropical regions is the genus *Piper. Piper* plants are also known under the common name ”pepper”. The presence of oil cells in the structures of almost all *Piper* species places them in the group of aromatic plants [8]. Besides their well-known uses as culinary spices, the secondary metabolites isolated from *Piper* plants show wide ranging human health effects.

One of the most extensively studied compounds isolated from *Piper* plants is piperlongumine, also known as piplartine. Piplartine is an amide alkaloid found in several *Piper* species (Piperaceae). It has been shown that piplartine has potential anticancer properties [9,10]. Piplartine also shows benefits in the treatment of the parasitic infection schistosomiasis, caused by helminth flatworms of the genus *Schistosoma* [11]. Compounds from *Piper tuberculatum* fruits show antiplasmodial and antileishmanial activities [12]. All these activities of *Piper* plants on neglected tropical diseases are very important for pantropical regions, which are the natural habitats of these plants. Piperlongumine shows anti-inflammatory effects in the central nervous system (CNS). In relation with inflammation-related brain diseases, such as Alzheimer’s disease (AD), Parkinson’s disease (PD) and Huntington’s disease, one of the potential approaches in prevention and treatment of these diseases is normalization of microglia activity. The anti-neuroinflammatory effects of piperlongumine are characterized as inhibition of the production of nitric oxide (NO) and prostaglandin E_2_ (PGE_2_) induced by lipopolysaccharide (LPS), also reducing the expression of inducible nitric oxide synthase and cyclooxygenase-2 as well as proinflammatory cytokines such as tumor necrosis factor (TNF)-α and interleukin-6 (IL-6), and also by suppressing the nuclear factor kappa B (NF-κB) signaling pathway [13].

Antimicrobial activity of *Piper* plants has been shown in the treatment of chronic periodontitis [14], as well as in the treatment of gastric pathogen *Helicobacter pylori* [15] and decreased *H. pylori* toxin entry to gastric epithelial cells [16]. In addition to the abovementioned pharmacological activities of *Piper* plants, different investigations have also indicated that these plants are active as anti-diabetic, anti-ulcer, diuretic, and local anesthetic agents [17]. Most of the information about the various biological activities of *Piper* plants has been derived from in vitro studies, while in vivo and toxicology studies are still somewhat limited. However, it can be noticed that these plants have multi-targeting potential, and their underlying mechanisms of action are waiting to be explored [18].

## 2. Habitat and Cultivation of *Piper* Plants

*Piper nigrum* is a member of family Piperaceae and is originally native to India. The plant is well known for its medicinal properties. It is the most commonly used spice, thus also called “the King of Spices”. Different types of black peppers are available having different colors. The most commonly known peppers are black and white peppers [19,20]. Kali Mirch is a common name for black pepper in Urdu, while green pepper, Madagascar pepper, white pepper, and black pepper are its common names in English. Black pepper has a wide range of applications. It is used as medicine, as a preservative and is also used in perfumes [21]. The active components of *P. nigrum* are used in foods as well as medicine. Pepper is used in sauces and meat dishes throughout the world. It contains an alkaloid called piperine which is known for its remarkable pharmacological actions, including antioxidant, antihypertensive and antiplatelet, antiasthmatics, analgesic, antitumor, antipyretic, antispasmodic, antidepressant, antidiarrheal, anxiolytic, anti-inflammatory, immunomodulatory, hepatoprotective, antifungal, antibacterial, antimutagenic, larvicidal, insecticidal, and many other activities [22]. Piperine inhibits several metabolic enzymes and increases the oral bioavailability of many vaccines, drugs, and nutrients ultimately enhancing their therapeutic effects. Piperine also helps in digestion by stimulating the intestinal and pancreatic enzymes. Piperine is the only main constituent responsible for most of the therapeutic actions of this spice. The fruits of *P. nigrum* are utilized to produce green and white peppers [23]. The fruit of *P. nigrum*, also known as peppercorn, in mature form seems dark red while in the dried form it appears as a small black wrinkled drupe with a diameter of 5 mm. The black pepper is normally produced by cooking the unripe fruits of pepper plant in hot water. White pepper is also produced from the same plant. White pepper is most common in Western countries. It is comprised of seeds only. For this purpose, the fully ripe pepper berries are soaked in water for few days which results in softening of the fruit flesh ultimately leading to its decomposition. The skin is then rubbed off, resulting in the naked seed. The seeds are then dried. Ground black pepper, most commonly known as pepper, is normally found on every dining table alongside table salt around the world [23].

### 2.1. Habitat of Piper nigrum L.

*P. nigrum* prefers hot and moist places for growth. The plant exists in a wide range of diverse conditions ranging from high elevations to various soils and climatic conditions. It is found in all the tropics as well as subtropics of the world. The geographic distribution of black pepper is influenced by the minimum temperature of the coldest month and the wettest month’s precipitation. The physiological activities in the wettest periods are at their maximum. During the wettest period, the flowering occurs, fills grains and ripens. This period requires a lot of water [24]. Similarly, *P. nigrum* requires an adequate amount of rainfall and humidity. The ideal conditions for its growth and cultivation are the hot and humid climates. Black pepper successfully grows between 20° North and South latitudes. The temperature between 10 to 40 °C is optimum for its growth [25].

Black pepper is naturally distributed in India, where the Western Ghat forests are rich in this plant. The biodiversity hotspots in this area are reported to be the only known existing source of wild *P. nigrum* germplasm in the world. Different studies have reported the modeling of the bioclimatic distribution of black *P. nigrum* in Asia with the help of ecological niche modeling (ENM), Maxent software and WorldClim bioclimatic data [25]. The ENM tool has been applied to many problems in evolutionary ecology, invasive-species management, biogeography, and conservation. The distributions of crops in ecological and geographical locations can be outlined using the ENM tool. It is useful for the introduction and conservation of species. It is also useful in applications where detailed information regarding the geographic distribution of species is required [26].

ENM-based studies predict that the eastern as well as western coasts of the Indian Peninsula, various regions of the Malay Archipelago, the southeast coastal areas of China and the eastern part of Sumatra Island consist of areas having the highest probabilities (>50%) [27]. Certain undocumented regions were also predicted to be suitable. The biological characteristics of the species and visual assessment of the predicted regions were like each other. The Malabar Coast in India is the origin of black pepper. Black pepper was then taken to Malaysia, Indonesia, and other countries. India has about 2000 years history of black pepper cultivation, during which black pepper expanded its geographical range. It is understandable that bioclimatic and current distributions of black pepper are basically similar.

For instance, Sen et al. [26] assessed the geographical distributions of pepper species by ENM tool and concluded that according to the future climate scenarios, the habitat of *P. nigrum* will witness reduction in the Western Ghat Forests in India [26]. Thus, such requirements limit dispersal of this species and do not allow the species to gain larger geographical distribution. Similarly, the conditions of Hainan province of China are more suitable for cultivation of black pepper. The temperature of this area is usually between 22.5 to 25.6 °C with 1640 mm of average rainfall each year. Such conditions have low management cost, great efficacy, and a better output [25].

### 2.2. Cultivation of Piper nigrum L.

The Western Ghats forests are considered as a primary center for cultivation of black pepper. It is believed that black pepper was first domesticated in this region many centuries ago. The cultivation of pepper was the then introduced to other countries in Asia and on other continents such as Sri Lanka, Malaysia, Madagascar, Brazil, Latin America, some African countries and many Southeast Asian countries. It is found in almost every country of southeast and southern Asia, except Bhutan and Pakistan. Thailand is the largest producer in terms of kilograms per hectare (3595) while India leads in the annual production in terms of tonnage (average 191,000 tonnes per annum). As reported by International Trade Centre of Geneva, the current trade in spices has been calculated to be 400,000 to 450,000 metric tons which worth 1.5 to 2 billion US dollars annually. Thirty-four percent of the total trade is contributed by black pepper. In 2014, its production in the world reached 462,955 tons [28]. As reported by Food and Agriculture Organization (FAO), 433,238 tons of peppercorns were produced by cultivating black pepper on 553,144 hectares of land in 2008 [25]. Does not add anything to the previous sentence which has more recent data. By 2020, the consumption of *P. nigrum* may reach 280,000 metric tons around the globe [29]. Check this number – if 463000 tons in 2014 it is expected to grow to 280000000 in 6 years?

China produces 27,210 tons of black pepper annually and is considered the fifth largest producer in the world. The Hainan province of China contributes 90% of the country’s annual black pepper production [25]. Black pepper is cultivated in Africa, Asia, and Latin America, which makes it a widespread species [30]. Although cultivated for thousands of years, black pepper is yet to be introduced in many regions. It is very important to determine the suitability of those regions for its cultivation. Scientists and naturalists are investigating the geographical distributions of many plant species and their relationship with environmental changes.

Studies were carried out to check the suitability for the cultivation of black pepper. Such studies reported that there are suitable areas in North Vietnam where black pepper could be introduced and cultivated in the future [31]. Conclusively, there is much need to conserve the wild varieties of black pepper. This is required to preserve the genetic resources for use in the future. This can be done through model prediction along with the distribution map of black pepper’s wild varieties. The conservation efforts can further be improved by understanding gene flow patterns and genetic structure of wild black pepper. Move above-this is “habitat”

## 3. Chemical Constituents of the Essential Oils of *Piper* Species

The *Piper* genus is an extremely well known and widely distributed pantropical taxon of aromatic plants, many of which have been used in the past as food and medicinal plants. *Piper* plants are rich in essential oils (EOs), which can be found in many tissues and organs: fruits, seeds, leaves, branches, roots and stems [32,33]. The chemistry of these EOs has been extensively studied since the 1960s, but according to Dyer [8] only about 10% of *Piper* species species (112 of 1457 accepted species world-wide, www.theplantlist.org) has been phytochemically studied. For this review it was possible to find chemical information relative to the volatile components for around 130 species, although only for around 16 of them the information is detailed enough.

Due to the wide variations in chemical content, and the different tissues from which the EOs were obtained, it is difficult to summarize the nature of the *Piper* EOs. According to Mgbeahuruioke et al. [33] there are more that 270 identified compounds in *Piper* EOs. In the review by Xiang et al. [34] the authors state that more than 80 compounds were identified in Chinese *Piper* spp., and that these compounds belonged mainly to the mono- and sesquiterpene hydrocarbon classes, followed by aldehydes, alcohols, acids, ketones, esters, and phenols. Some species have a simple profile, while others, such as *P. nigrum, P. betle, P. auritum*, contain very diverse suites of secondary metabolites. According to Mgbeahuruioke et al. [33] it is possible to show several differences in chemical composition of the EOs of fruits and of leaves/aerial parts. Fruit EOs are less chemically diverse than EOs from aerial parts, sesquiterpene hydrocarbons and oxygenated compounds are, generally speaking, the most important chemical classes in fruits and aerial parts, and β-caryophyllene is the most important compound in fruit EOs, while it is less important in the leaves.

Generally speaking the EOs are rich in all classes of volatile chemical compounds, but the composition is highly variable, both inter and intra specifically, and these differences seem to depend on polymorphism, plant part, geographical differences, environmental conditions and chemotypes [32,35]. Amongst the most important compounds found in the EOs there are, for monoterpene hydrocarbons: α-pinene, myrcene, limonene, α-terpinene, *p*-cymene, β-pinene, α-phellandrene, (*Z*)-β-ocimene; for oxygenated monoterpenoids: 1,8 cineole, linalool, terpinen-4-ol, borneol, camphor; for sesquiterpene hydrocarbons: β-elemene, β-sesquiphellandrene, (*Z*)-β-bisabolene, (*Z*,*Z*)-α-farnesene, *ar*-curcumene, α-zingiberene, δ-cadinene, β-caryophyllene, α-humulene, germacrene D, bicyclogermacrene, α-cubebene; for oxygenated sesquiterpenoids: spathulenol, (*E*)-nerolidol, caryophyllene oxide, α-cadinol, *epi*-α-bisabolol; and for phenylpropanoids: safrole, dillapiole, myristicin, elemicin, (*Z*)-asarone, eugenol, apiole, and sarisan [32].

Thin et al. [35] have proposed a more complex division of *Piper* spp. EOs into six groups, according to the dominating chemical classes:

(A) EOs dominated by monoterpene compounds
  - *Piper demeraranum*: limonene, sabinene, β-pinene and α-pinene.
  - *Piper chimonanthifolium*: piperitone
  - *Piper cubeba*: sabinene and 1,8-cineole
(B) EOs dominated by sesquiterpene compounds
  - *Piper majusculum* leaf: β-caryophyllene, germacrene D and β-elemene
  - *Piper cernuum*: β-elemene and *epi*-cubebol
  - *Piper madeiranum*: β-caryophyllene and germacrene D-4-ol
  - *Piper duckei* germacrene D and β-caryophyllene
  - *Piper nigrum:* β-caryophyllene
  - *Piper lepturum* var. *lepturum*: β-guaiene
  - *Piper lepturum* var. *angustifolium*: β-bisabolene
(C) EOs dominated by both monoterpene and sesquiterpene compounds.
  - *Piper hispidum*: α-copaene and α-pinene
  - *Piper demeraranum*: limonene and β-elemene
  - *Piper aduncum*: camphor, viridiflorol and piperitone
(D) EOs dominated by phenylpropanoid compounds
  - *Piper caninum, Piper auritum, Piper hispidinervum*: safrole
  - *Piper aduncum*: dillapiole
  - *Piper divaricatum*: methyleugenol and eugenol
  - *Piper betle*: chavibetol
  - *Piper patulum*: 1,3,5-trimethoxy-2-propenylbenzene
  - *Piper klotzsdhianum*: 2,4,5-trimethoxy-1-propenylbenzene
  - *Piper marginatum*: (Z)-asarone
(E) EOs dominated by benzenoid compounds
  - *Piper klotzsdhianum*: 1-butyl-3,4-methylenedioxybenzene, 1-butyl-3,4-methylenedioxybenzene and 1-butyl-3,4-methyl- enedioxybenzene
  - *Piper sarmentosum*: benzyl benzoate, benzyl alcohol, 2-hydroxy-benzoic acid phenylmethyl ester and 2-butenyl-benzene
  - *Piper harmandii*: benzyl benzoate and benzyl salicylate
(F) EOs dominated by non-terpenoid compounds
  - *Piper maclurei*: methyl oleate
  - *Piper caldense*: pentadecane

As suggested above, there are chemical differences which many authors have declared to be due to the existence of chemotypes. According to da Silva et al. [32] there is evidence of chemotypes for at least seven of the Neotropical species: *Piper aduncum* leaf EOs seem to come in nine chemotypes (CTs), dominated by 1,8-cineole, (*E*)-nerolidol, dillapiole, and asaricin; *Piper amalago* has monoterpenoid and sesquiterpenoid CTs; *Piper cernuum* leaf oils are dominated by dihydroagarofuran, monoterpene hydrocarbons or sesquiterpene hydrocarbons; *Piper divaricatum* shows two CTs, an eugenol/methyl eugenol CT and a safrole CT; *Piper marginatum* from Brazil is dominated by phenylpropanoids but there are wide variations in the specific molecules; *Piper hispidum* leaf EOs from Cuba are dominated by eudesmol, those from Panama by dillapiole, and those from Colombia by (*E*)-nerolidol; and finally *Piper umbellatum* EOs from Costa Rica and Brazil are dominated by sesquiterpene hydrocarbons, while those from Cuba by camphor and safrole.

According to Mgbeahuruioke et al. [33] the African *Piper* spp. EOs show great chemical diversity and presence of chemotypes. The authors cite seven chemotypes of *Piper guineense* form Nigeria (β-caryophyllene/germacrene D; asaricin; α-pinene/β-pinene/germacrene B; β-pinene/α-pinene/β-caryophyllene; β-pinene/α-pinene/1,8-cineole; linalool) and three chemotypes of *P. guineense* from Cameroon (α-pinene/β-pinene; β-caryophyllene/limonene//pinenes; β-caryophyllene) [33].

Notable differences between Neotropical and African general composition is the greater quantitative importance of the monoterpene hydrocarbons β-pinene, α-phellandrene, and (*Z*)-β-ocimene, and of the sesquiterpene hydrocarbons β-elemene, β-sesquiphellandrene, (*Z*)-β-bisabolene, (*Z*,*Z*)-α-farnesene, *ar*-curcumeme, α-zingiberene, and δ-cadinene. On the other hand the African species are less rich in the monoterpene hydrocarbons myrcene, α-terpinene, and *p*-cymene, and in the sesquiterpene hydrocarbon α-cubebene.

### 3.1. Piper aduncum L.

The composition of the EOs is quite variable, and these degrees of chemical polymorphism have been attributed both to genetic differences and to changing environmental conditions [36]. In a 2017 review of the Neotropical species of *Piper*, the authors write that at least nine different chemotypes of *P. aduncum* have been characterized: there is evidence of at least two chemotypes from Equadorian specimens, one from the western Amazonian region characterized by dillapiole (31.5% to 97.3%) [32,37] and the second from the Atlantic Forest dominated by terpenoid compounds such as (*E*)-nerolidol and linalool [38]. The EOs from Panama are high in sesquiterpenes such as β-caryophyllene and aromadendrene, those from Bolivia are high in the monoterpene 1,8-cineole [39], and those from the Amazon, Malaysia and Cuba are rich in phenylpropanoids, namely in dillapiole (1.5–97.3%; 64.5%; 58% respectively) [40].

There are also differences in the composition of EOs distilled from different organs. According to Navickiene et al. [38] who distilled Brazilian specimens, fruit EOs are dominated by monoterpenes (an average of 85.1%), leaf EOs are dominated by sesquiterpenes (specifically by β-caryophyllene), while the rare root EO contains mainly monoterpenes but at lower level than fruits (an average of 66.9%).

On the other hand, there is a very high variability even in EOs from the same organs: da Silva et al. [32] describe leaf EOs rich in monoterpenoids (1,8-cineole), sesquiterpenoids ((*E*)-nerolidol), or phenylpropanoids like dillapiole or asaricin; an EO from the leaves of *P. aduncum* var. *ossanum* from Cuba was mainly composed of piperitone (20.1%), viridiflorol (13.0) and camphor (13.9%) (see Table 1 and Table 2). The EOs from the aerial parts of specimens from the Americas, southeast Asia and Oceania seem dominated by dillapiole (30–90%) but specimens from Bolivia were dominated by 1,8-cineole (40%), those from Panama were rich in sesquiterpenes like β-caryophyllene and aromadendrene, those from Brazil were rich in linalool or (*E*)-nerolidol and surprisingly devoid of phenylpropanoids, those from Equador were abundant in dillapiole, piperitone and (*E*)-β-ocimene [36]. The EOs from aerial parts of Cuban plants were particularly rich in oxygenated compounds, both monoterpenoids (50.3%) and sesquiterpenoids (29.2%), with lower amounts of monoterpene hydrocarbons (9.7%) and sesquiterpene hydrocarbons (8.2%) [41] (see Table 3 and Table 4). The EO from inflorescences from Brazilian samples consisted mainly of oxygenated monoterpenoids (43.9%), followed by sesquiterpene hydrocarbons (30.5%) and phenylpropanoids (10.0%) [32,42] (See Table 5).

There is evidence of at least two chemotypes of leaf EOs from Equadorian specimens, one from the western Amazonian region characterized by dillapiole (31.5% to 97.3%) [43] and the second from the Atlantic Forest dominated by monoterpenoid compounds such as (*E*)-nerolidol and linalool [32]

Da Silva et al. [32] describe EOs of aerial parts from two different chemotypes, one from Equador with dillapiole at 45.9%, (*E*)-β-ocimene at 19.0%, and piperitone at 8.4%; and one from Cuba with piperitone between 19.0% and 23.7%, camphor between 9.4% and 17.1%, and viridiflorol between 13.0% and 14.5%; the review of the literature reveals a general preference for dillapiole in the species from the Neotropical region, and a clear distinction for the specimen from China, dominated by eugenol.

Da Silva et al. [32] report the composition of *P. aduncum* stem EO from Brazil, which comprises α-pinene (7.2%), β-pinene (14.2%), limonene (8.7%), (*Z*)-β-ocimene (5.5%), (*E*)-β-ocimene (13.3%), linalool (11.8%), β-caryophyllene (7.6%), α-humulene (6.3%), and (*E*)-nerolidol (10.6%), and the composition of a root EO, which comprises α-selinene (14.1–16.5%), geranyl 2-methylbutyrate (8.9–13.6%), bulnesol (4.6–6.1%), elemicin (4.6–5.9%), dillapiole (13.0–18.4%), and apiole (16.3–29.5%).

### 3.2. Piper amalago L.

The majority of analyses on *P. amalago* EOs have been done on leaves, and are summarized in Table 6. Although from limited evidence, there seems to be a prevalence of sesquiterpene hydrocarbons such as bicyclogermacrene, β-phellandrene and germacrene D, with significant amounts of monoterpene hydrocarbons such as α-pinene, and some sesquiterpenoids such as spathulenol [50].

Da Silva et al. [32] report the compositions of Brazilian inflorescence EOs, which show striking differences. The main compounds of the first EO are: *allo*-aromadendrene (18.5%), silphiperfol-6-ene (13.5%), limonene (10.5%), *p*-cymene (9.3%) and α-muurolol (5.0%), while those of the other ones are (*E*)-nerolidol (14.2–19.9%), germacrene D-4-ol (10.3–12.7%), α-cadinol (8.2–11.1%), β-phellandrene (7.3–8.2%), bicyclogermacrene (3.0–9.1%), τ-cadinol (4.9–6.1%), and δ-cadinene (2.3–6.6%).

Da Silva et al. [32] report the main components of the EO of aerial parts of Brazilian specimen: limonene (20.5%), zingiberene (11.2%), δ-elemene (6.8%), and α-pinene (5.2%). They also report the main components of a Brazilian, stem-only EO: longifolene (6.6%), α-amorphene (23.3%), and α-muurolol (9.3%). Another stem EO from Brazil was analyzed by dos Santos et al. [50] and again it shows a different chemical breakdown from the EO described by da Silva et al. [32]: bicyclogermacrene (12.01%), α-cadinol (9.43%), isocaryophyllene (8.32%), γ-muurolol (8.29%), (*E*)-nerolidol (5.24%), spathulenol (4.38%), and γ-cadinene (3.77%). Although different from each other, these EOs shows a dominance of sesquiterpenes and sesquiterpenoids.

According to da Silva et al. [32] the EO from the roots of Brazilian *P. amalago* contain α-amorphene (14.4%).

### 3.3. Piper betle L.

According to Burfield [51] a typical EO from *Piper betle* leaves is dominated by phenylpropanoids and aromatic compounds, can contain up to 40% eugenol, and up to 40% of carvacrol and chavicol taken together, while chavibetol is characteristic of the EOs from the whole plant. Other typical compounds are α terpinene, *p*-cymene, 1,8-cineole, β-caryophyllene, α-humulene, allyl pyrocatechol, allylcatechol, methyl eugenol, and estragol (methyl chavicol).

In a preliminary work on *Piper betle* leaf EO from Sri Lanka, the authors identified (but didn’t quantify) the following compounds [52]: safrole, eugenol, allyl pyrocatechol diacetate, chavibitol acetate, β-phellandrene, terpinen-4-ol, estragole, anethole, 1,8-cineole, linalool, α-pinene, and methyl eugenol.

The review of the published data by Lawrence [53] confirms the description by Burfield [51], in that the EOs are dominated by phenylpropanoids such as chavicol (range 1–48%), chavibetol (range 2–69%) chavibetyl acetate (range 13–20%), eugenol (range 9–63%), eugenyl acetate (range 2–19%), and safrole (range 40–48%). (*E*)-Isoeugenol results significantly elevated in two specimens from Thailand (28.32%) and Vietnam (72%). β-Caryophyllene is the only non-phenylpropanoid that is present in many different samples in significant amounts (range 3–11%) [53]. Table 7 summarizes Lawrence’s [53] data plus data from papers published after the review.

In a Chinese study that analyzed the EOs from leaves and stem (aerial parts), the main components were (*E*)-isoeugenol (10.3–44.8%), δ-cadinol (9.32%), caryophyllene oxide (8.78%), spathulenol (8.00%), propiopiperone (5.75%), α-cadinol (5.62%), caryophyllenol II (3.06%), and δ-cadinene (2.91%) [34].

### 3.4. Piper cubeba Bojer

According to Burfield [51] the EOs from the fruits of *Piper cubeba* are mainly composed of sesquiterpene hydrocarbons, and specifically β-caryophyllene, δ-cadinene, α- and β-cubebenes, and minor amounts of monoterpenes. The survey by Lawrence [60] describes a similar scenario, the sesquiterpene hydrocarbons represent the main class of constituents, with α-copaene, β-cubebene, *allo*-aromadendrene, γ-muurolene and germacrene D the most important ones, followed by δ-cadinene and β-caryophyllene. However the review underlines the importance of the monoterpene sabinene, present in high amounts (up to 29.6%) [60]. For the complete chemical breakdown, see Table 8 below.

Later studies tend to confirm these studies, and find that the main compounds are the cubebenes (between 5.6% and 22.8% in 3/5 of the reviewed studies), sabinene (between 9.6% and 19.99% in 3/5 of the studies), α-copaene (between 0.9% and 8.8% in 4/5 of the studies), germacrene D (between 1.5% and 7.5% in 4/5 of the studies), β-caryophyllene (between 0.4% and 5.3% in 3/5 of the studies), *allo*-aromadendrene (between 2.3%v and 4.10% in 3/5 of the studies), δ-cadinene (between 0.2% and 4.7% in 2/5 of the studies). For the complete chemical breakdown see Table 9.

### 3.5. Piper nigrum L.

Narayanan [62] recognizes around 135 compounds in these EOs, belonging to the monoterpenoid, sesquiterpenoid, aliphatic, aromatic and other chemical groups. He states that generally speaking the EOs are composed by 70–80% of monoterpene hydrocarbons (mainly α-pinene up to 13%, β-pinene up to 40%, limonene up to 32%), 20–30% sesquiterpene hydrocarbons (mainly β-caryophyllene up to 22%) and less than 4% oxygenated constituents [62]. A recent paper on different types of Chinese *P. nigrum* (black, white and green) confirms these ranges with 39.74–64.67% monoterpene hydrocarbons, 1.85–3.44% monoterpenoids, 20.87–43.89% sesquiterpene hydrocarbons and 2.21–10.81% sesquiterpenoids [63].

As with many of the EOs of the *Piper* genus, the chemical composition is however extremely varied, and Narayanan [62] considers taxonomical differences (varieties), geography, maturity of the raw material, and differences in distilling parameters and analytical techniques as the principal causes of this variety. Major compound classes recognized by Narayanan [62] are the following:

(1) *Monoterpene hydrocarbons*: camphene, δ-3-carene, *p*-cymene, limonene, myrcene, (*Z*)-β-ocimene, α-phellandrene, β-phellandrene, α- and β-pinenes, sabinene, α- and γ-terpinenes, terpinolene and α-thujene.

(2) *Oxygenated monoterpenoids*: borneol, camphor, carvacrol, *cis*-carveol, *trans*-carveol, carvone, carvotanacetone, 1,8-cineole, cryptone, *p*-cymene-8-ol, *p*-cymene-8-methyl ether, dihydrocarveol, dihydrocarvone, linalool, *cis*-*p*-mentha-2,8-dien-1-ol, *p*-mentha-3,8-dien-1-ol, *p*-mentha-1(7),8-dien-1-ol, 1 (7)-*p*-menthadien-4-ol, *p*-mentha-1,8-dien-5-ol, *p*-mentha-1,8-dien-4-ol, *cis-p*-menth-2-en-1-ol, myrtenal, myrtenol, methyl carvacrol, *trans*-pinocarveol, pinocamphone, *cis*-sabinene hydrate, *trans*-sabinene hydrate, terpinen-4-ol, 1-terpinen-5-ol, α-terpeneol, phellandral, piperitone, citronellal, nerol, geraniol, isopinocamphone, methyl citronellate, methyl geranate, α-terpenyl acetate, terpenolene epoxide and *trans*-limonene epoxide.

(3) *Sesquiterpene hydrocarbons*: *cis*-α-bergamotene, *trans*-α-bergamotene, β-bisabolene, β-carophyllene, α- and β-cadinenes, calamenene, α-copaene, α- and β-cubebenes, *ar*-curcumene, β- and δ-elemene, β-farnesene, α-guaiene, α-humulene, isocaryophyllene, γ-muurolene, α-santalene, α- and β-selinenes, ledene, sesquisabinene and zingiberene.

(4) *Sesquiterpenoids*: 5,10(15)-cadinadien-4-ol, caryophylla-4(12),8(13)-dien-5β-ol, β-caryophyllene alcohol, caryophyllene ketone, caryophyllene oxide, epoxy-dihydrocaryophyllene, (*Z*)-nerolidol, cubenol, *epi*-cubenol, viridiflorol, α- and β-bisabolols, cubebol, elemol and eudesmol.

(5) *Miscellaneous compounds*: eugenol, methyl eugenol, myristicin, safrole, benzaldehyde, (*E*)-anethole, piperonal, *m*-methylacetophenone, *p*-methylacetophenone, butyrophenone, benzoic acid, phenylacetic acid, cinnamic acid and piperonic acid.

Lawrence [64,65,66] has published three reviews on *P. nigrum.* Table 10 summarizes his finding relative to the main compounds in black and white *P. nigrum* [64,65], while Table 11 details the data from his latest review [66]. Table 12 summarizes the main data from papers published after Lawrence’s reviews or not taken into account by the author.

The roots of *P. nigrum* from China were analyzed and the EO was found to contain sabinene (< 0.2%) and δ-3-carene (10.9–21.1%) [72]. Leaves and stems of a Chinese plant were distilled and the main compounds characterizing the EO were β-caryophyllene (13.8%), spathulenol (6.22%), and caryophyllene oxide (6.00%) [34].

### 3.6. Piper longum L.

A very recent review by Lawrence [73] is summarized in the Table 13 below, from which it can be seen that the EOs seem to be characterized by non-terpenoid compounds such as pentadecane and heptadecane isomers, and by sesquiterpene hydrocarbons such as germacrene D, β-caryophyllene, and β-selinene. In only one case the characterizing compound is a phenylpropanoid, eugenol.

### 3.7. Piper arboreum Aubl.

The EO of *Piper arboreum*, and specifically that obtained from the leaves, is on average dominated by sesquiterpenoids, particularly by sesquiterpene hydrocarbons and secondarily by their oxygenated derivatives; monoterpenoids only play a minor role. According to dos Santos et al. [74], the EO from the leaves contains 65.85% of sesquiterpenes, 22.59% as hydrocarbons and 43.26% as oxygenated derivatives, and only 4.28% of monoterpenes (see Table 14).

da Silva et al. [32] report the composition of a Brazilian EO from the flowers, dominated by sesquiterpene hydrocarbons. The main components were germacrene D (49.3%), linalool (10.4%), germacrene A (8.5%), β-caryophyllene (6.6%), limonene (6.3%), and β-elemene (5.3%) [32]. The same authors report of the composition of a Brazilian EO obtained from the stems which again is dominated by sesquiterpene hydrocarbons: β-caryophyllene (26.5%) and bicyclogermacrene (21.1%), followed by δ-3-carene (18.7%), and α-copaene (9.0%) [32]. Navickiene et al. [38] report that the fruits of *P. arboreum* contain 74.4% sesquiterpenes and 22.6% monoterpenes, while the root EO contains 72.2% sesquiterpenes and 25.6% monoterpenes.

### 3.8. Piper auritum Kunth

From the scant data available it appears that EOs from all tissues of *Piper auritum*, are dominated by phenylpropanoids, and specifically by safrole. Data collected and reviewed by da Silva et al. [32] describe three EOs distilled from leaves, one from Panama, characterized by safrole (70%), and two from Cuba, characterized one by safrole (64.5%) and camphene (5.5%), and the second by safrole (71.8%). A more recent paper describes a Colombian EO containing as main components safrole and myristicin [75]. An EO obtained from the aerial parts of plants from Cuba contains safrole at 86.9%, γ-terpinene at 1.32% and terpinolene at 1.11% [76], while a Colombian one contains safrole at 91.3%, and an EO from the flowers, distilled in Costa Rica, has the highest level of safrole: 93.2% [32].

### 3.9. Piper cernuum Vell.

The chemical composition of the leaf oils (from Brazil) have been reviewed by da Silva et al. [32], and can be divided in two groups, one dominated by dihydroagarofurans and the other with a more conventional composition, with monoterpene and sesquiterpene hydrocarbons as major components. See Table 15.

A similar subdivision can be observed even in other organs’ EOs. An EO from aerial parts of Brazilian plants contained, as major compounds, trans-dihydroagarofuran at 31.0%, elemol at 12.0%, and 10-epi-γ-eudesmol at 13.0%, while an EO from the flowers (again from Brazil) contained α-copaene at 6.5%, β-caryophyllene at 9.8%, germacrene D at 14.3%, bicyclogermacrene at 6.5%, and spathulenol at 9.7%. The authors also analyzed the EO from the branches, and the main compounds present were quite distinct from the other EOs: camphene (46.4%), p-cymene (5.8%), linalool (8.7%), α-terpineol (11.6%), and carvacrol (11.6%) [32].

### 3.10. Piper dilatatum Rich.

The EOs from the leaves seem to be dominated by hydrocarbons, usually monoterpenes, and secondarily by the oxygenated derivatives. An EO from Brazil contained (*E*)-β-ocimene (19.7%), β-caryophyllene (11.4%), germacrene D (8.9%), bicyclogermacrene (8.8%), spathulenol (6.5%), caryophyllene oxide (5.3%), but a second EO, again from Brazil, contained myrcene (41.77%) and α-pinene (17.7%) as main monoterpene hydrocarbons, and 9-*epi*-caryophyllene (2.15%), bicyclogermacrene (1.51%), β-caryophyllene (2.18%), and δ-cadinene (1.41%) as major sesquiterpene hydrocarbons, with 1,8 cineole at 2.7% and 2-tridecanone at 4.39% [32].

There are more data on EOs derived from aerial parts, and they were reviewed by da Silva et al. [32], showing a clearer dominance by sesquiterpene hydrocarbons, and specifically by germacrene D (6.7–43.0%), bicyclogermacrene (6.7–34.7%), and β-caryophyllene (5.1–11.7%), with a sesquiterpenoid such as spathulenol at 9.3–40.6%. See Table 16.

### 3.11. Piper gaudichaudianum Kunth

According to Schindler and Heinzmann [77] the literature on *P. gaudichaudianum* EOs reports a generic composition characterized by sesquiterpene hydrocarbons (22.5 to 36.4%), with smaller percentages of oxygenated sesquiterpenoids (0.2 to 5.8%), trace amounts of monoterpene hydrocarbons and no oxygenated monoterpenoids. However, the authors found in their analyses that the EOs from leaves and reproductive organs had a strikingly different composition, and were dominated by the phenylpropanoid class, and in particular by dillapiole, which in fresh leaves was at levels between 68.4% and 69.2%, and in the inflorescences between 83.1% and 87.5% [77]. Myristicin was also present in the reproductive organs in the range of 6.2 to 11.9%. This high percentage of phenylpropanoids left less room for other constituents, but the main ones were α-humulene (13.3–37.5%), β-caryophyllene (10.4–19.3%), β-pinene (5.6–7.0%), (*E*)-nerolidol (5.32–22.4%), β-caryophyllene (8.9%), bicyclogermacrene (7.4%), β-selinene (3.7–15.7%), α-selinene (8.9–16.6%), *allo*-aromadendrene (7.7%) and linalool (4.8%) [77]. See Table 17.

### 3.12. Piper hispidum Sw. (Including References to the Synonym Piper hispidinervum C.DC.)

As with many other *Piper* spp. EOs, the composition of *P. hispidum* EOs is very variable, although from limited evidence it can be proposed that monoterpene and sesquiterpene compounds are the most common ones. There are reports of leaf EOs from Cuba rich in eudesmols [24], from Panama rich in dillapiole [48] and from Colombia rich in (*E*)-nerolidol [79]. Dos Santos et al. [74] describe EOs mainly composed of sesquiterpenoids, and da Silva et al. [32] describe an EO rich in monoterpenes (α-pinene at 15.3% and β-pinene at 14.8%). A *Piper hispidinervum* (sic) leaf EO contained 53.98% sesquiterpene hydrocarbons, 33.06% of monoterpenoids and no trace of phenylpropanoids [47]. Table 18 summarizes the data on the main compounds of *P. hispidum* (and *P. hispidinervum)* leaves.

### 3.13. Piper guineense Schumach. & Thonn

There have been several reports of *P. guineense* fruit EOs. The composition is varied and the main compounds have been identified as monoterpenes, sesquiterpenes and phenylpropanoids [81], and there is evidence of the existence of many chemotypes, the most important ones the dillapiole CT, β-caryophyllene CT, β-pinene CT, linalool CT [82], and the relatively new type, myristicin + ishwarane CT from Cameroon [83]. Martins et al. [69] describe the aerial parts EO from S. Tomé e Príncipe with elevated amounts of dillapiole and myristicin, and Jirovetz et al. [81] describe a black *P. guineense* fruit EO from Cameroon with, as major compounds, β-caryophyllene, β-elemene, bicyclogermacrene and α-humulene, and the white version of the fruit containing mainly β-caryophyllene, (*Z*)-β-ocimene, limonene, β-pinene, linalool and α-humulene. Oyedeji et al. [83] also cite EOs from Cameroon dominated by α-pinene, β-pinene; from Nigeria dominated by myristicin and savisan; and from Congo dominated by linalool, myristicin and β-caryophyllene. An EO from Nigeria is characterized by monoterpenoids at 55.6% [83], while a second EO again from Nigeria is dominated by sesquiterpenoids at 64.4% and monoterpenoids at 21.3% [83], and an EO from Colombia has total sesquiterpenes at 74.4%, with oxygenated sesquiterpenoides at 46.4%, followed by sesquiterpenes hydrocarbons at 28.0% [79]. Table 19 summarizes recent data.

### 3.14. Piper marginatum Jacq.

According to da Silva et al. [32] the EOs from the leaves appear to be characterized by the presence of phenylpropanoids such as safrole and propiopiperone, but with a high variability of concentration. However, a recent paper that used a cluster-analysis technique on the composition of 22 samples of EO from leaves from Brazil claims to have found evidence for at least seven different chemotypes [86]:

- Chemotype I: safrole and propiopiperone
- Chemotype II: propiopiperone and *p*-menthα-1(7),8-diene
- Chemotype III: propiopiperone, myristicin, (*E*)-β-ocimene, γ-terpinene
- Chemotype IV: β-caryophyllene, α-copaene, and propiopiperone
- Chemotype V: (*E*)-isoosmorhizole, (*E*)-anethole, and isoosmorhizole
- Chemotype VI: 2-methoxy-4,5-(methylenedioxy)propiophenone, methoxy-4,5-(methylene- dioxy)propiophenone isomer 5, (*E*)-isoosmorhizole.
- Chemotype VII: β-caryophyllene, bicyclogermacrene, (*E*)-asarone.

In conclusion, it appears that the plant metabolism, although always producing phenylpropanoids as main constituents, can be driven to produce propiopiperone and related compounds (CT 1-4 and 6) or compounds like (*Z*)-anethole, (*E*)-anethole, isoosmorhizol, and nothosmorhizol (CT 5-7) [86]. Table 20 summarizes the results of two reviews on *P. marginatum* leaf EO compositions, and of three papers not reviewed previously [75].

The composition of the stem EO has been rarely studied; in one case from Brazil the main compounds were (*E*)-asarone (32.6%) and patchouli alcohol (25.7%), followed by (*Z*)-asarone (8.5%), elemicin (6.9%), β-caryophyllene (6.8%), seychellene (5.8%), (*E*)-methyleugenol (3.6%) and α-copaene (2.7%) [87]. In another case the main compound was still a phenylpropanoid, myristicin (19.3%), followed by propiopiperone (18.6%), β-caryophyllene (11.6%) and δ-3-carene (6.9%) [32].

The EO from the aerial parts seems also dominated by phenylpropanoids, as shown by a sample from Costa Rica with as the principal compound (*E*)-anethole (45.9%), followed by *p*-anisaldehyde (22.0%), anisyl methyl ketone (14.2%), estragole (6.6%), and *p*-cymene (7.1%) [87]. A sample from Colombia is still characterized by phenylpropanoids but with a very different composition: elemicin (18.0%), α-phellandrene (11.1%), β-caryophyllene (11.0%), limonene (7.5%), isoelemicin (9.2%), β-elemene (4.0%), and bicyclogermacrene (4.0%) [75]. Of the three Brazilian samples reviewed by da Silva et al. [32], two had a a characteristic phenylpropanoid composition, the first contained isoosmorhizol (32.2%), (*E*)-anethole (26.4%), and (*Z*)-isoosmorhizole (11.2%), while the second contained propiopiperone (21.8%), elemol (5.9%), and β-caryophyllene (5.0%). The third sample however has as the main compound *p*-menthα-1(7),8-diene (39.0%), followed by (*E*)-β-ocimene (9.8%), and propiopiperone (19.0%) [32]. A Brazilian paper details the composition of an EO from the flowers of a Brazilian *P. marginatum* sample: the composition is patchouli alcohol (23.4%), (*E*)-asarone (22.1%), (*E*)-caryophyllene (13.1%), α-acoradiene (9.7%), α-copaene (9.4%), and (*Z*)-asarone (4.5%) [87].

### 3.15. Piper umbellatum L.

da Silva et al. [32] found that *P. umbellatum* EOs from Costa Rica and from Brazil, were rich in sesquiterpene hydrocarbons. Brazilian EOs had as main compounds germacrene D (34.2–55.8%), δ-cadinene (15.0%), bicyclogermacrene (9.0–11.8%) γ-muurolene (8.9%), β-caryophyllene (6.3%), and γ-cadinene (5.9%) [32]. A recent paper confirms these findings, with the three main compounds β-selinene (16.12%), bicyclogermacrene (10.64%) and β-caryophyllene (5.35%); EOs from Costa Rica were quite similar, with main compounds β-caryophyllene (28.3%), germacrene D (16.7%), (*E*,*E*)-α-farnesene (14.5%) β-elemene (6.9%), and bicyclogermacrene (6.6%); the EO from the Cuba was rich in camphor (9.6%), and safrole (26.4%), with lower amounts of β-caryophyllene (6.6%) [78]. Martins et al. [69] found that the EO from aerial parts of *P. umbellatum* from S. Tomé e Príncipe were characterized by β-pinene (26.8%), α-pinene (17.6%), and (*E*)-nerolidol (12.4%).

### 3.16. Piper tuberculatum Jacq.

*P. tuberculatum* EO from seeds was mostly composed of β-elemene, β-caryophyllene and β-farnesene, among others [88]. In the review by da Silva et al. [32] three leaf EO samples from Brazil, all dominated by sesquiterpenoids, have been compared. In two cases the most important compound was β-caryophyllene at 26.3% and 40.2%, and in the third one it was spathulenol at 15.8% [32]. A fruit EO sample from Brazil was instead dominated by monoterpene hydrocarbons (73.95%), mainly by β-pinene (27.74%) and α-pinene (26.54%), with sesquiterpene hydrocarbons at 22.8%, and β-caryophyllene at 14.38%. The oxygenated compounds for both mono and sesquiterpenes only summed up to 7% [88]. See Table 21.

Brazilian leaf EOs are composed mainly by sesquiterpene hydrocarbons with smaller amounts of sesquiterpenoids and monoterpene hydrocarbons [32,38,44,78]. Ordaz et al. [89] describes an EO from Venezuela with as main component spathulenol (11.37%).

da Silva et al. [32] report the main compounds in the EO obtained from the flowers as α-pinene (28.7%), β-pinene (38.2%), (*E*)-β-ocimene (9.8%), and β-caryophyllene (14.0%), and the main compounds in the EO obtained from the stems as: α-pinene (17.3%), β-pinene (27.0%), (E)-β-ocimene (14.5%), and β-caryophyllene (32.1%). 

To summarize the main chemical groups characterizing the 16 species analyzed above, following Thin and et al. [35] subdivision, we have:

(1) EOs dominated by monoterpene compounds: *P. aduncum, P. cubeba, P. dilatatum, P. nigrum, P. hispidum, P. guineense.*
(2) EOs dominated by sesquiterpene compounds: *P. aduncum, P. amalgamo, P. cubeba, P. nigrum, P. arborescens, P. tuberculatum, P. umbellatum, P. cernuum, P. dilatatum, P. gaudichianum, P. hispidum, P. guineense.*
(3) EOs dominated by both monoterpene and sesquiterpene compounds: *P. aduncum, P. cubeba, P. dilatatum, P. nigrum, P. hispidum, P. guineense.*
(4) EOs dominated by phenylpropanoid compounds: *P. aduncum, P. betle, P. auroitum, P. gaudichiuanum, P. guineense, P. marginam.*

### 3.17. Other Piper Species

The following tables (Table 22, Table 23, Table 24, Table 25, Table 26 and Table 27) summarize the main data regarding the remaining *Piper* species for which there is scant data. The tables are subdivided according to the origin of the EO.

*Piper acre*. An EO from Vietnam contained, as main compounds, sabinene (19.9%), (*E*)-nerolidol (15.6%), δ-cadinene (13.5%), benzyl benzoate (7.0%), and β-phellandrene (2.6%) [35].

*Piper caldense*. An EO, according to Salleh et al. [92], was characterized by terpinen-4-ol (18.5%), α-terpineol (15.3%), caryophyllene oxide (6.2%), and α-cadinol (9.8%). 

*Piper caninum*. An EO, according to da Silva et al. [32], was characterized mainly by safrole (25.5%), similar to *P. auritum* (leaf EO, 70%), *Piper callosum* (leaf EO, 70%), *P. hispidinervum* (leaf EO, 81–88%), *P. betle* (floral EO, 27.6%), *Piper mikanianum* (leaf EO, 82%) and *Piper xylosteoides* (aerial parts EO 47.83%). The other main compounds were β-caryophyllene (9.8%), germacrene D (7.8%), β-pinene (4.9%), δ-elemene (4.1%), linalool (2.9%), limonene (2.7%), eugenol (2.4%), τ-muurolol (2.4%), bicyclogermacrene (2.3%), camphor (0.3%), α-cubebene (0.5%), β-cubebene (0.3%), aromadendrene (0.8%), *allo*-aromadendrene (0.3%), α-bisabolene (0.4%), germacrene B (1.1%), globulol (0.3%), α-cadinol (1.0%) and farnesyl acetate (1.2%) [32].

*Piper carniconnectivum*. An EO from Brazil, according to da Silva et al. [32], was characterized by spathulenol (23.7%), β-pinene (19.0%), α-pinene (8.0%), and caryophyllene oxide (7.8%).

*Piper laosanum*. An EO from Vietnam contained, as main compounds, sabinene (14.9%), benzyl salicylate (14.3%), (*E*)-nerolidol (9.3%), and (*Z*)-α-copaene-8-ol (4.5%) [35].

*Piper caldense* root EO was characterized by valencene (10.5%), pentadecane (35.7%), and selina-3,7(11)-diene (5.4%) [32].

One sample of *Piper capense* fruit EO was dominated by monoterpene hydrocarbons such as β-pinene (33.2%), sabinene (10.0%) and α-pinene (8.9%) [106]. *Piper gibbilimbum* EO from Papua New Guinea was dominated by the rare alkenylphenol derivatives gibbilimbols, specifically gibbilimbol A (46.0%), gibbilimbol C (19.2%) and gibbilimbol B (7.7%), plus other more common compounds such as camphene (13.6%) and α-pinene (6.5%) [40].

The EO from *Piper carpunya* flowers from Peru was characterized by high levels of safrole (32.0%) and 1,8-cineole (30.2%), followed by α-terpinene (9.8%), *p*-cymene (7.7%), and α-pinene (6.2%) [32].

The EOs from *Piper claussenianum* flowers from Brazil were dominated by the monoterpene alcohols linalool (50.2–56.5%) and (*E*)-nerolidol (22.7–24.3%), followed by α-humulene (2.4%) [32].

*Piper consanguineum* EO from Peru contained camphor (25.3%), camphene (22.4%), isoborneol (12.8%), α-pinene (4.8%), β-bisabolol (4.5%) [107].

The EO from leaves, roots, seeds and stems of the plant *Piper klotzschianum* contain as main compounds 4-butyl-1,2-methylenedioxybenzene (36.9–96.2%), limonene (17.8%), α-phellandrene (17.0%), γ-asarone (5.4–9.1%), *p*-cymene (7.4%), *trans-*α-bergamotene (8.8%) [101].

*Piper majusculum* EO from Indonesia contained no monoterpenes, but was dominated by sesquiterpenes: β-caryophyllene (17.27%), caryophyllene oxide (14.26%), α-selinene (14.21%), *cis*-calamenene (9.62%), spathulenol (8.76%), α-bergamotene (7.02%), α-copaene (5.88%), (*Z*)-α-bisabolene (4.95%), β-bisabolene (4.13%), δ-cadinene (2.80%), α-cubebene (2.64%), α-humulene (2.25%) [108].

## 4. Traditional Uses of *Piper* Species

According to Wan Salleh et al. [109,110] the genus *Piper*, which belongs to the Piperaceae family, consist of five subgenera and roughly 1400 species distributed throughout the tropical and subtropical regions. A literature search led to the identification of 1048 species as provided by the website www.theplantlist.org of which only 143 species were scientifically reported in PubMed search engine. Of these, only 93 species were reported or cited to have been used traditionally for medicinal purposes (based on searches using the PubMed, Researchgate and Semantic Scholar search enginea). Further searches using the Yahoo and Google search engines lead to the indentification a number of other new species (not recorded), of which only 19 species were reported to posess traditional medicinal uses. Overall, 106 species were identified to possess medicinal values and used traditionally in various parts of the tropical and subtropical regions. The list of *Piper* species and their traditional medicinal uses are reported below.

### 4.1. Piper abbreviatum Opiz

The paste of leaves of *Piper abbreviatum* is used externally by the peoples of Philippines to treat splenomegaly. In addition, the fruits are utilized to treat coughs and colds as well as for flatulence [111]. Wan Salleh et al. [110] also reported *P. abbreviatum* to have a carminative effect.

### 4.2. Piper aduncum L.

*P. aduncum* is traditionally used to treat stomach aches, vaginitis, influenza, rheumatism, cough, fever and general infections [112,113]. In Mexico it is used to treat urological problems, dermatological conditions, and skin tumors [114]. In the Caribbean region, the leaves and roots are made into tea and used to treat diarrhea, dysentery, nausea, ulcers, genito-urinary infections. In addition to these usages, *P. aduncum* is also traditionally used as an antihemorrhagic agent to control bleeding [36,115]. Other than that, its EO is a well-known insecticide, molluscicide and antibacterial.

In Africa, as well as Jamaica, an infusion of leaves from *P. aduncum* is used to treat stomach pains [116] while in Papua New Guinea *P. aduncum* is used in as an antiseptic [117].

In Papua New Guinea folk medicine, the extract of the leaves of *P. aduncum* is used to treat insect bites, dressing sores and cuts, and scabies whereas the extract of the barks is used for the treatment of toothache, diarrhea, dysentery, scabies, cuts, cough and fungal infections. Moreover, the roots are used to treat stomach and respiratory ailments, skin wounds and dysentery; while extracts of the stem and fruits are used for treating headache and toothache, respectively [17]. In the traditional medicine of Indonesia, *P. aduncum* is used to treat burns [17].

In Latin America, traditional uses of *P. aduncum* have been recorded in Brazil, Colombia, Honduras and Peru. In the Brazilian Amazon, the leaves of *P. aduncum* are used to treat intestinal and stomach ailments, erysipelas, cystitis, gynecological inflammation, disorders of the digestive tract, wound healing, pyelitis, influenza and and as an insect repellent [17]. The native cultures of Honduras, on the other hand, used the leaves, fruits and stems of *P. aduncum* for treatment of female disorders, pains, as digestive and skin cleanser [17] whereas in Colombia, extracts of the plant are used to treat dysentery and hemostasis [17]. Furthermore, the extracts of *P. aduncum* leaves are used in Peru for treatment of diarrhea with the aerial parts also applied against rheumatic afflictions, and as an astringent, styptic and antiseptic. Interetingly, the Yaneshas tribe living in the Peruvian Amazon used teas and steam baths from the *P. aduncum* leaves for general infections and fever [17]. Other than those countries, *P. aduncum* leaves and fruits are used as antimycotic, antimicrobial and styptic in various part of the eight Amazon regions [17].

Other medicinal usages reported for *P. aduncum* include as a digestive, anti-gonorrhea, antiblennorrhea, stomachic, anti-hemorrhagic agents [118] and as a diuretic, anti-inflammatory, antidiarrheal agents [119].

### 4.3. Piper boehmeriifolium (Wall. ex Miq.) C.DC.

The roots of *Piper boehmeriifolium* are used in the Ayurvedic system of Indian medicine as laxative, anthelmintic, and carminative agents. In addition, *P. boehmeriifolium* also is used in the treatment of bronchitis, spleen diseases, and tumors [120].

In China, *P. boehmeriifolium* is traditionally used for promoting blood circulation and, thus, claimed to be useful in antiplatelet therapies and in the treatment of thrombotic diseases [121]. Other than that, *P. boehmeriifolium* is also used to alleviate pain and, to treat rheumatism and arthritic conditions [122].

### 4.4. Piper sylvaticum Roxb.

The roots of *Piper sylvaticum* are applied in the Ayurvedic system of Indian medicine to treat bronchitis, diseases of the spleen, and tumors, and also for for their laxative, anthelmintic, and carminative properties [120]. Chahal et al. [115] also cited that the roots of *P. sylvaticum* are widely used in Ayurvedic medicine as an effective antidote to snake poison.

### 4.5. Piper capense L.f.

In Cameroon, *P. capense* is reported to be used for treating cancer [123,124], while the aerial part of *P. capense* is traditionally used in Comoro Islands for diarrhea and cough [115,125]. Other reports have cited the traditional uses of *P. capense* in the treatment of abdominal pain, diarrhea, and cough [126]. An extensive discussion on traditional usages of *P. capense* can be found on the Useful Tropical Plants Database website [127]. According to the author’s report, the fruit is considered to be carminative, diuretic, stimulant and stomachic and vermifuge, and when prepared as infusion can be taken to treat stomach problems, including indigestion, flatulence and colic; heart and kidney problems; and as a cough medicine. Moreover, the leaf preparations are used to treat including abdominal disorders; bilious fever; kwashiorkor; hematuria; bacterial skin infections; epileptic attacks; and polio. On the other hand, the root is said to be anthelmintic, the sweetened root infusion or seed extract is consumed to alleviate cough, the raw or cooked root is eaten as an aphrodisiac tonic while the root is applied to the soles of the feet to treat paralysis of patients suffering from cerebral bleeding. Further, the macerated bark is drunk to treat sore mouth and throat; chest complaints; and venereal diseases, the infused bark is used to treat sterility while the pulverized bark is applied on wounds and against vaginal discharge.

### 4.6. Piper cubeba L.

*P. cubeba* has been listed in Moroccan and Chinese traditional medicine as one of the importance plants for the treatment of cancer [128]. Other than that, *P. cubeba* has also been reported to be used traditionally to treat renal disorder, gonorrhea, syphilis, abdominal pain, enteritis and asthma [129].

Extensive discussion on traditional usages of *P. cubeba* found in the website known as ‘Useful Tropical Plants Database’ [127] revealed that *P. cubeba* is a bitter, antiseptic and stimulant herb with its fruits having diuretic and expectorant effects while also improving digestion. In addition, the immature dried fruits are used in the treatment of coughs, bronchitis, sinusitis, throat and genito-urinary infections, poor digestion and amebic dysentery.

### 4.7. Piper gibbilimbum C.DC.

The juice squeezed from heated bark of *P. gibbilimbum* with traditional ash salt are used in Papua New Guinea, to treat cancer or other internal sores [130].

### 4.8. Piper guineense Schum and Thonn

Uhegbu et al. [131] have reported that the seeds of *P. guineense* are used by the people of Nigeria to relieve stomach discomfort caused by excess gas, as an adjuvant in the treatment of rheumatic pains, as anti-asthmatic or aphrodisiac agents, to control weight, consumed by women after childbirth to improve uterine contraction for expulsion of a placenta and other remains from the womb, taken by lactating mothers during postpartum period to encourage or stimulate uterine contractions, hence, help to return the uterine muscle to its original shape or as abortifacient agent.

Besong et al. [132] have also reported that the leaves of *P. guineense*, which are considered aperitif, carminative and eupeptic, are used by the Nigerian to treat respiratory infections, rheumatism and syphilis to relieve flatulence, to treat female infertility and low sperm count in male while the fruits are used as an aphrodisiac. On the other hand, the roots are chewed and juice swallowed as an aphrodisiac and used as chewing sticks for cleaning the teeth. Furthermore, Soladoye et al. [133] and Kuete et al. [123] have also reported that the Nigerian and Cameroonian used seeds possess anticancer properties. Besong et al. [132] have also reported that the fruit extract of *P. guineense* is used in China to treat epilepsy. Nwosu and Okafor [134] have also reported on *P. guineense* usage in the treatment of eczema (tinea versicolor), common cold and fever in humans.

### 4.9. Piper longum L. (syn. P. latifolium Forst.; P. chaba Hunter)

*Piper longum* is used in traditional medical practice in the Cook Islands wherein the leaves are pounded in a wooden bowl with little water and used to wash the chest of a person with suspected breast cancer [135]. This application of *P. longum* to treat tumors has also been recorded in Indian Ayurvedic medicine. Other than that, roots and fruits of *P. longum* are also being used in India, especially in Western Ghats and central Himalayas regions, as an antidote to snake bite and scorpion stings, and to treat chronic bronchitis, cough and cold. Moreover, the fruits have been used in traditional remedies against intestinal distress with the ripe fruits also applied as an alternative to tonic [115]. Moreover, Sireeratawong et al. [136] also reported that the dried mature unripe fruits of *P. longum* are widely applied as carminative, element tonic, antidiarrheal, expectorant and oxytocic for postlabor in Thai traditional medicine while Naz et al. [137] stated that *P. longum* is also used for pain alleviation, fever and piles without referring to the part of *P. longum* used.

An extensive review by Fern et al. [127] revealed that the fruits of *P. longum* are used internally in traditional Chinese medicine to treat stomach chills, vomiting, acid regurgitation, headache and rhinitis while in Ayurvedic medicine it is also used to treat colds, asthma, bronchitis, arthritis, rheumatism, lumbago, sciatica, epilepsy, indigestion and wind. The fruits were also claimed to help improves the digestion and possessed decongestant, antibiotic and analgesic effects. Externally, the fruit is utilized to treat toothache. On the other hand, the root is considered diuretic, stimulant and sudorific.

In Bangladesh, specifically in the Satkhira–Bagerhatt area of Khulna division, *P. longum* is used in folk medicine wherein the root, which is alexiteric, is useful for treating asthma and bronchitis while helping to improve consumption; the fruit, which is pungent, thermogenic, anthelmintic, expectorant, stimulant and carminative, helps to improve appetite and taste, and is useful for treating hemorrhoidal infections, asthma, bronchitis, fever, inflammation, piles, pain in the abdomen and at the anus; and the stem is used to alley post-delivery pain in mothers and also useful in rheumatic pains and diarrhea [138].

### 4.10. Piper nigrum L.

The root of *P. nigrum* is used by the people of Thailand in the form of ghee, powders, enemas, and balms to treat to abdominal tumors, abdominal fullness, adenitis, cancer, cholera, cold, colic, kidney stone, asthma and headache [139]. In addition, the plant is also used in traditional Chinese medicine to treat epilepsy [140] and applied in some formulae to treat respiratory or gastric cancers in China [141,142]. *P. nigrum* is also used in traditional Middle Eastern medicine as a nerve tonic [140]. In traditional Ayurvedic medicine, *P. nigrum* is used in combination with *P. longum* to treat intermittent fevers, to promote the secretion of bile and recommended for neurological, broncho-pulmonary and gastrointestinal disorders, (including dyspepsia, flatulence, constipation and hemorrhoids) [140]. Moreover, Agbor et al. [143] also reported on the folk medicine uses of fruits and leaves of *P. nigrum* for the treatment of coughs, intestinal diseases, bronchitis, venereal diseases, and rheumatism while Aziz et al. [144] cited the general application of *P. nigrum* for treating diarrhea, fever, cold, colic disorder and gastric conditions.

Fern et al. [127] have extensively reported on the usages of *P. nigrum* as a stimulating expectorant in traditional Western and Ayurvedic medicines, and as a tranquilizing and anti-emetic in traditional Chinese medicine. Morevoer, the seed is applied internally in Western herbalism to treat indigestion and wind, and in Chinese medicine to treat stomach chills, food poisoning, cholera, dysentery, diarrhoea and vomiting caused by cold. Furthermore, *P. nigrum* is used externally in Ayurvedic medicine to treat nasal congestion, sinusitis, epilepsy and skin inflammations with the EO used to ease rheumatic pain and toothache.

### 4.11. Piper cavalcantei Yunck.

The leaves of *Piper cavalcantei*, in a form of decoction, is considered by native Amazon people as an excellent antipyretic and analgesic agent [32,145].

### 4.12. Piper marginatum Jacq.

*P. marginatum* leaves and stems are used in Brazil, especially in Paraíba State, against snake bites and as a sedative [146] while in north-eastern Brazil and in the northern region, especially in the Amazon, *P. marginatum* is commonly used for the treatment of inflammatory diseases, snake bites, as well as the liver and bile duct diseases [87]. In addition, the indigenous communities in Central America, the Antilles, and South America used *P. marginatum* for gastrointestinal problems [147]. Other than that, Almeida et al. [44] also reported the uses of *P. marginatum* as a tonic, carminative, stimulant, diuretic, and sudorific agents against stomach, liver and gall-bladder pain, toothaches, and snake and insect bites while da Silva et al. [119] reported *P. marginatum* to have antispasmodic actions. A preparation made from the leaves of *P. marginatum* is used in French Guyana to treat malaria while in Trinidad and Tobago, Puerto Rico and Surinam, *P. marginatum* has been used to treat female disorders and to help during childbirth [17]. Furthermore, *P. marginatum* is also traditionally used in Brazil to treat asthma, erysipela, problems of the urinary system, vesicle and liver diseases, and to control blood pressure [17].

### 4.13. Piper umbellatum L.

*P. umbellatum* is traditionally used as an anti-inflammatory in Brazil, to treat wounds in Cuba, and to treat fever in Peru [148], to treat onchocerciasis in Cameroon [149] and, to treat wounds by various west African tribes [117].

Generally, Roersch [150] also reported that *P. umbellatum* is traditionally used to treat a wide range of ailments such as kidney disease, women diseases, diarrhea, skin afflictions, burns, rheumatism, malaria, intestinal parasites, inflammation and fever in 24 countries in three continents, America, Africa and Asia with additional uses, namely for treating miscarriages, boils, dermatosis and leucorrhea, recorded by Céline et al. [112] and Calderón et al. [151]. Agbor et al. [143] have reported the uses of *P. umbellatum* as a spiritual plant associated with mystical powers and shaman and also as a sedative. In addition to the above claims, the infusion of *P. umbellatum* leaves is also used in the treatment of infectious and inflammatory diseases [150]. In recent report, Durant-Archibold et al. [17] have highlighted the traditional medicinal uses of different parts *P. umbellatum* applied in different manners (decoctions, infusions, maceration, and teas) for the treatment of the urinary tract infections, skin and liver ailments, contusions, digestive problems, pains, wound healing, swelling, rheumatism, women’s diseases, as antipyretic and anti-inflammatory.

In addition to the above scientific writings, Fern et al. [127] in his website has made an extensive report on the traditional uses of *P. umbellatum*. The leaves of *P. umbellatum* are extensively applied in various forms to treat different types of ailments as listed below. The leaves are used as an antseptic, emollient, vermifuge and vulnerary; the leaf juice is taken as a diuretic, emmenagogue and galactagogue, used as ear drops to remedy earache or eye drops to remedy conjunctivitis; a decoction of leaves is consumed to treat hypertension, toothache, jaundice, malaria, urinary and kidney problems, syphilis and gonorrhea, leucorrhea, menstrual problems and stomach-ache, used as a wash for feverish children, or applied on wounds and inflamed tumors; the crushed leaves are utilized in the form of an enema to treat rectal prolapse; an infusion of young ground-up leaves is taken to treat severe colic; the aerial parts are commonly given to women to regulate menses and prevent abortion, and; the leaves are also taken in order to expel tapeworms, while suppositories of the leaves are used to rid the body of pinworms, used in massages for relieving migraine and other forms of headache, and are applied in a friction to relieve rheumatic pain, and applied as a poultice on swellings, boils and burns. In Brazil, the leaves of *P. umbellatum* are used in baths to subdue edema and uterine complaints. The leaves and fruits are used to treat pain in the kidneys, edema, anemia and colic while the fruits of *P. umbellatum* are chewed with *P. betle* leaves to treat coughs. On the other hand, the root of *P. umbellatum* is considered diuretic, febrifuge and stimulant, and also to promote the flow of bile while a decoction of its root is used to improve digestion and to treat dyspepsia, constipation, jaundice, malaria, urinary and kidney problems, syphilis and gonorrhea, leucorrhea, menstrual problems and stomach-ache or applied externally to treat wounds and inflamed tumors. Moreover, the roots are macerated in alcohol and used to treat rheumatism. Meanwhile, a mixture of pounded twigs and seeds, and salt is taken to treat intestinal worms, a tea made from the flower clusters is used in the treatment of coughs.

### 4.14. Piper aborescens Roxb.

*Piper aborescens* has been traditionally used to treat rheumatism and posseses cytotoxic activity and antiplatelet aggregation [152].

### 4.15. Piper acutifolium Ruiz and Pav.

*Piper acutifolium* has been reported to be used traditionally as antiseptic and to treat wound healing, vaginal infections, gastritis, skin ulcerations and ailments [153,154].

### 4.16. Piper alatabaccum Trel. & Yunck

*Piper alatabaccum* is traditionally used to treat stomach aches and diarrhea [155].

### 4.17. Piper angustifolium Lam.

*Piper angustifolium* is traditionally used as an antiseptic and to treat cutaneous leishmaniasis-associated lesions, stomatitis, vaginitis and liver disorders [156]. Fern et al. [127] has extensively reported on the traditional uses of *P. angustifolium*. According to the report, an EO prepared from the leaves of *P. angustifolium* is an aromatic stimulant, diuretic and astringent; the leaves are used internally in the treatment of gastric and intestinal problems, including peptic ulcers, diarrhea and dysentery, and commonly used in Bolivia and Peru to treat internal bleeding such as rectal bleeding and hemorrhoids, and bleeding in the urinary tract. The leaves, applied externally as a decoction, are also valuable remedy for minor wounds, insect stings and inflamed skin, or used as a mouthwash and douche.

### 4.18. Piper auritum Kunth

*P. auritum* is traditionally used to treat fever and sore throat [157]. According to Durant-Archibold et al. [17], *P. auritum* is used as diuretic, antipyretic, for gout, angina, erysipelas, venereal diseases, colic, and headache as well as an appetite stimulant, local anesthetic, and wound poultice. The Chinantec tribe in Mexico drank the decoction of *P. auritum* leaves to facilitate childbirth and as an emmenagogue while the Mayan tribe used the plant traditionally for healing wounds. In Guatemala, *P. auritum* is used as galactagogue and for the treatment of dysmenorrhea; in Panama, Columbia as well as Guatemala, the juice of crushed leaves or the decoction of roots are drunk or used in baths for snakebites or rubbed onto the body as a snake repellent; in El Salvador and Ecuador, respectively, the juice of *P. auritum* leaves is applied to remove ticks and head lice; in Costa Rica, the fresh leaves of *P. auritum* are used to treat headaches; in Panama, the Gunas tribes drunk the infusion of *P. autritum* leaves to treat common colds; and in Mexico, *P. auritum* is applied against dermatological illnesses [17,158].

### 4.19. Piper barbatum Kunth

Several reports revealed the traditional medicinal uses of *Piper barbatum* to include for treatment of headache, stomach pain, dermatitis, disinfectant, and healing of wounds [151,159].

### 4.20. Piper betle L.

The leaves of *P. betle* have been traditionally used in India, China and Thailand for prevention of oral malodor due to its anti-bacterial activity, as a mouth freshener and masticatory, for their wound healing property [160], to enhance digestive and pancreatic lipase stimulant activities [161], for prevention of catarrhal and pulmonary afflictions [162] and, for preventing secretion or bleeding as well as an aromatic stimulant and anti-flatulent agent [163]. In addition, the leaf of *P. betel* is also traditionally valued as an aphrodisiac [163] and for the treatment of a range of diseases such as halitosis, boils and abscesses, conjunctivitis, constipation, headache, hysteria, itches, mastitis, mastoiditis, leucorrhea, otorrhea, ringworm, swelling of gums, rheumatism, abrasion, cuts, injuries as well as scabies, mouth odor, cough remedy, bronchitis, and nosebleed while the root is famous for its female contraceptive effects [118,164,165,166,167]. Ding et al. [121] have also reported on *P. betle* traditional uses in China for promoting blood circulation and is claimed to be useful in antiplatelet therapies and in the treatment of thrombotic diseases.

Dwivedi and Tripathi [168] have cited the Ayurverdic medicinal uses of *P. betle* leaves in a variety of decoctions to cure wounds, urticaria, burns, impectigo, furuneloris, eczema, lymphangitis, asthma and rheumatism, with its juice having a beneficial stomatic, stomachic and febrifuge effect, and used to treat pharyngitis, abdominal pain and swelling. Moreover, a paste of *P. betle* leaves is applied on cuts and wounds or mixed with salt and hot water to treat filariasis; the mixture of *P. betle* and *P. nigrum* leaf is consumed to cure obesity; the leaves of *P. betle* are mixed with mustard oil, warmed and are applied to the chest of children and old people for treatment to reduce cough and dyspnea while the juice of *P. betle* combined with honey is also used to treat coughs, dyspnea, and in indigestion, among children; and the leaves of *P. betle* smeared with oil are useful on the breasts of lactating women to promote milk secretion. In addition, the leaves can also be applied locally to cure inflammatory swelling such as orchitis, arthritis and mastitis; used to improve bad breath, body odor and prevent tooth decay; used to prevent and treat vaginal ejection, and reduce itching of the vagina; and used to stop bleeding in the nose. They also revealed the uses of *P. betle* roots and fruits to treat malaria and asthma with the roots also used in combination with *P. nigrum* to generate sterility in women while the oil prepared from *P. betle* is for irritation of the throat, larynx, bronchi, gargle and inhalation in diphtheria.

Fern et al. [127] reported that the leaves of *P. betle* are anthelmintic, antibacterial, antifungal, antiseptic, aphrodisiac, astringent, carminative, expectorant, laxative, sialagogue, stimulant, stomachic and tonic, and used to treat nosebleed, ulcerated noses, gums and mucous membranes while the leaf preparations and the leaf sap are applied to wounds, ulcers, boils and bruises. The leaf extract is applied for wounds in the ears and as an infusion for the eye while the leaf decoction is used to bathe a woman after childbirth, or is drunk to lessen an unpleasant body odor. Furthermore, the heated leaves of *P. betle* are applied as a poultice on the chest against cough and asthma, on the breasts to stop milk secretion, and on the abdomen to relieve constipation.

### 4.21. Piper claussenianum (Miq.) C. DC.

*P. claussenianum* has been traditionally used to treat candidiasis and vaginal infections [169].

### 4.22. Piper cumanense Kunth

*Piper cumanense* has been traditionally used to treat malaria and fever [170].

### 4.23. Piper dennisii Trel.

*Piper dennisii* has been traditionally used to treat rheumatic pain and arthritis [112].

### 4.24. Piper fimbriulatum C. DC.

*Piper fimbriulatum* has been traditionally used in Panama to treat pain and has shown antiplasmodial activity [171,172].

### 4.25. Piper glabratum Kunth

*Piper glabratum* has been traditionally used to treat skin ailments, skin ulcerations, wounds and as an antiseptic [151,154].

### 4.26. Piper grande Vahl

*Piper grande* has been traditionally used in Panama to treat leishmaniasis-associated lesions and has demonstrated antiplasmodial activity [172].

### 4.27. Piper hayneanum C.DC.

*Piper hayneanum* has been traditionally used to treat wounds and skin diseases [173].

### 4.28. Piper hispidum L.

*P. hispidum* has been traditionally used to treat wounds and symptoms of cutaneous leishmaniasis, skin ailments, and stomach aches [115,154,174,175]. In addition, Michel et al. [176] reported that *P. hispidum* has been used to treat aches and pains in Nicaragua, to regulate menstruation in Peru, and to treat urinary infections in the Amazon. In Peru, the crushed leaves of *P. hispidum* are traditionally applied on the skin by the Chayahuitas, an Amazonian Peruvian ethnic group, to heal wounds and to treat cutaneous leishmaniasis while Facundo et al. [116] reported that the infusion of leaves from *P. hispidum* is used to treat stomach pains in Jamaica. In South and Central America, particularly Brazil, Colombia, Ecuador, Guatemala, Honduras, Mexico, Panama and Peru, *P. hispidum* has been ethnomedicinally used to treat snakebites, insect bites, head lice, amygdalitis and mouth sores, and as a skin cleansing, diuretic, teeth whitening, and antihemorrhagic agent [177].

Durant-Archibold et al. [17] have also reported on the folklore uses of *P. hispidum* in various South American countries. A leaf infusion is drunk for its antihemorrhagic and diuretic effects in Brazil while in Ecuador it is applied to kill head lice; a leaf decoction is used in Colombia to treat malaria while in Panama it is used to treat conjunctivitis, diarrhea and hemorrhages; the leaves are used as a remedy to treat female diseases by the Q’eqchi Maya tribe from Guatemala, to ease the pain of childbirth, anemia, and rheumatism in Nicaragua, to treat mumps and tonsillitis, and to prevent tooth decay by the Totonacs ethnic group from Mexico; to treat insect and snake bites, and as a skin cleanser in Honduras; and to treat stomach aches and colds when used in combination with *P. aduncum* by the people of Jamaica. In addition, the inflorescence is applied topically for muscle aches in Nicargua. Almeida et al. [44] also reported that the tea of the decoction of *P. hispidum* leaves is useful for the treatment of malaria while Lans et al. [158] reported the application of *P. hispidum* as remedies for colds, fever, stomach aches and for aches and pains in eastern Nicaragua and Jamaica.

### 4.29. Piper holtonii C.DC.

*Piper hotonii* is traditionally utilized in Panama to treat leishmaniasis symptoms [151,175] with specific usage for treating malaria symptoms recorded for the people of Colombia by Garavito et al. [170].

### 4.30. Piper jacquemontianum Kunth

*Piper jacquemontianum* is traditionally applied in Panama as a remedy for fever, headaches, colds, nervousness, diabetes, stomachache, as a digestive, and for pain [177].

In various Latin America countries, including Guatemala, *P. jacquemontianum* is used in folklore medicinal uses to treat skin ailments, infections, anemia and body aches [154,178].

### 4.31. Piper jericoense Trel. & Yunck

*Piper jericoense* has been reported to be traditionally utilized as antiplasmodial and cytotoxic agents [179].

### 4.32. Piper lanceaefolium HBK.

*Piper lanceaefolium* has been reported to be traditionally utilized to treat skin infection [180].

### 4.33. Piper methysticum G.Forst

In the Pacific Islands, *Piper methysticum* is traditionally valued for its relaxant properties and use as an alternative medication for anxiety, stress, and insomnia [181]. The roots of *P. methysticum***,** prepared as beverage, possess sedative effect, but if consumed in high concentrations it is anesthetic and hypnotic [118]. In addition, *P. methysticum* is also used in traditional systems of medicine to treat asthma, common cold, cystis, gonorrhea, headaches, menstrual irregularities, urinary tract infections and warts, and to induce muscle relaxation and reduce weight [182].

Fern et al. [127] has also extensively revealed the traditional medicinal uses of *P. methysticum* in his website. The roots of *P. methysticum*, either in fresh or dried form, are medicinally used for its diuretic and aphrodisiac effects, and ability to relieve pain, relax spasms and has a stimulatory effect upon the circulatory and nervous systems. It is consumed internally to treat genito-urinary infections, gall bladder complaints, arthritis and rheumatism with the root bark scrapings also chewed to soothe sore throats and toothaches. The root is also used externally to relieve joint pains. On the other hand, the leaves are chewed as a treatment for bronchitis, rubbed onto centipede bites, insect stings and stings from poisonous fish with the liquid pressed from the leaves used to treat convulsions and stiffness in children in Fiji. In addition, a leaf infusion is used to treat several types of inflammation and is used to treat watery vaginal discharges, the branches are used as a remedy for sore throats.

### 4.34. Piper multiplinervium C.DC.

*Piper multiplinervium* is used by Guna Indians of Panama to treat gastrointestinal ailments, snakebites, body aches, menorrhagia, to heal wounds, as a hemostatic, and for teeth whitening [177]. On the other hand, Durant-Archibold et al. [17] reported that the Gunas Amerindians drunk an infusion prepared from young leaves of *P. multiplinervium* to treat different types of pains, with Calderón et al. [172] specifically reported the use of *P. multiplinervium* for treating stomach aches.

### 4.35. Piper obrutum Trel. & Yunck.

*Piper obrutum* has been reported to be traditionally used as medicinal remedy because of its antiplasmodial and cytotoxic activity [179].

### 4.36. Piper ovatum Vahl

*Piper ovatum* has been reported to be traditionally used in Brazil as medicinal remedy because of its anti-inflammatory and analgesic effects [183].

### 4.37. Piper pulchrum C.DC.

*Piper pulchrum* has been reported to be traditionally used to treat hemorrhagic venom effect from snakebite and as antidote for snakebite [184].

### 4.38. Piper pyrifolium Vahl.

According to Fortin et al. [185], *Piper pyrifolium* is traditionally used to cure diarrhea and as a diuretic.

Fern et al. [127] reported that, other than diuretic, *P. pyrifolium* is also used as a depurative. A decoction of *P. pyrifolium* is applied to treat stomatitis in young children, and also to treat blennorrhagia. On the other hand, the fruits are febrifuge and stomachic, and are also used in the treatment of blennorrhagia while the stem internodes of *P. pyrifolium* are used in the treatment of asthma and neuralgia.

### 4.39. Piper regnellii (Miq.) C. DC.

*Piper regnelli* is traditionally used to treat wounds, swellings and skin irritations [186]. In addition, the leaf and root of *P. regnelli* are used as crude extracts, infusions or plasters to treat wounds, reduce swellings, and relieve skin irritations [187].

### 4.40. Piper retrofractum Vahl

*Piper retrofractum* is said to have stimulant and carminative effects, and help to improve digestion. It is also used to treat intestinal disorders and postpartum treatment in women, and to cure rheumatic pain and body pain after childbirth [188]. Specifically, the fruits are said to have stimulant, expectorant, carminative and anthelmintic effects, and traditionally used to improve appetite and taste, and to treat cough, cold, asthma, bronchitis, fever, piles and hemorrhoidal affections. In Thailand, the fruit of *P. retrofractum* is reported to be useful for treatment of bronchial asthma, bronchitis, muscle pain, and other maladies while in Indonesia the fruit is mixed in carminative and sudorific remedies. In addition, the root is alexiteric and medicinally useful in the treatment of asthma and bronchitis [188].

According to Fern et al. [127] the root of *P. retrofractum* is chewed and the saliva swallowed or a decoction of the root is drunk as a treatment for colic, dyspepsia and gastralgia while the salted and oiled leaves are heated over the embers of a fire and stroked over the entire body, from head to foot, for treating postpartum fevers and chills. In addition, the dried fruits are said to have antidiarrheal, aromatic, carminative, oxytocic, stimulant and stomachic effects, and traditionally used in the treatment of coughs and colds, and hemorrhoids.

### 4.41. Piper sanvicentense Trel. & Yunck.

*Piper sanvicentense* has been used in folklore medicine due to its anti-tumor and anticancer properties [189].

### 4.42. Piper sarmentosum Roxb.

*P. sarmentosum* is widely used in folklore medicine of the Asian and South East Asian regions [190]. In Malaysia, the leaves and roots of *P. sarmentosum* are applied to the forehead to relieve headache while its decoction is utilized to cure muscle weakness and pain in the bones. In Indonesia, the rootlets of *P. sarmentosum* are chewed with betel nut and the juice is swallowed to treat coughs and asthma, chewed with ginger to treat toothache or chewed with a little nutmeg and ginger to treat pleurisy. The warmed leaves coated with coconut oil are applied to the painful chest while the finely ground leaves mixed with small amount of water are smeared on the throat to treat coughs. In Thailand, the roots are used as carminative and stomachic while the fruits and leaves are used as an expectorant. Tuntiwachwuttikul et al. [191] also reported that the leaves and roots of *P. sarmentosum* are used for the treatment of toothache, foot dermatitis, cough and asthma in Malaysia and Indonesia. In China, *P. sarmentosum* is traditionally used for promoting blood circulation and claimed to be useful in antiplatelet therapies and in the treatment of thrombotic diseases [121].

Other than that, Fern et al. [127] also reported on the traditional uses of *P. sarmentosum* wherein the whole plant is reported to possess anodyne, anti-inflammatory and expectorant effects while the leaf is also claimed to have carminative effect. *P. sarmentosum* is also used to cure skin diseases, rheumatism, headache, diarrhoea and toothache.

### 4.43. Piper sintenense Hatus.

*Piper sintenense* is used in folklore medicine to treat snake bites and wounds [192].

### 4.44. Piper strigosum Trel. & Yunck.

*Piper strigosum* is used in folklore medicine to treat symptoms associated parasitosis and leishmaniasis, wounds [193].

### 4.45. Piper stylosum Miq.

*Piper stylosum* is used in folklore medicine to treat fever and pain [109]. The root of the plant is used as a poultice or decoction after confinement [109].

### 4.46. Piper tuberculatum Jacq.

*P. tuberculatum* is used in folklore medicine as antidiuretic, analgesic and sedative and antidote for snakebites and to treat digestive disorders [10]. In some northeastern Brazil communities, especially in Paraíba State, the leaves and stems of *P. tuberculatum* have been used as antidote for snake bite and sedative [146,194]. In addition to the above usages, Burci et al. [195] have also reported on the wide used of leaves and fruits infusions of *P. tuberculatum* in Brazilian folk medicine as an analgesic. In addition, the leaves are used as a hemostatic in venomous snake bites in Eastern Colombian [196] and to treat dermatological illnesses in Mexico [158].

### 4.47. Piper xanthostachyum C. DC

*Piper xanthostachyum* is used in Panama folklore medicine to treat leishmaniasis symptoms [172].

### 4.48. Piper carpunya Ruiz & Pav (syn: P. lenticellosum C.D.C.)

The leaves of *P. carpunya* are widely used in folk medicine of tropical and subtropical countries of South America, especially Ecuador, as an anti-ulcer, antidiarrheal, anti-inflammatory and anti-parasitical remedy as well as an ailment for skin irritations [197,198].

### 4.49. Piper obliquum Ruiz & Pavon

*Piper obliquum* leaves are utilized in the Central and South America, especially Guyana and Ecuador, as analgesic or antiarthritic by topic application on the affected body part [36,115]. Specifically, the Ketchwa Indians in Ecuador used *P. obliquum* to treat dental problems while the warmed leaves of *P. obliquum* is used by the Patamona Amerindians in Guyana for treating pains, muscular aches and for arthritis. In addition, the stem of the plant is used in the French Guiana folk medicine to treat hernia.

### 4.50. Piper laetispicum C. DC

*Piper laetispicum,* popularly known in the southern part of China folk medicine, is used for reducing stasis and promoting blood circulation, which are claimed to be useful in antiplatelet therapies and in the treatment of thrombotic diseases [121], and as an analgesic [115]. Moreover, the extracts of *P. laetispicum* are used in Brazilian folk medicine to reduce blood pressure.

### 4.51. Piper arboreum Aubl.

A decoction of *P. arboreum* has been used in the Brazilian traditional medicine against venereal diseases and infections of the urinary throat [199,200] and to treat sexually transmitted diseases, bronchitis, urinary infections, rheumatic problems and as carminative [17,201]. Moreover, *P. arboreum* is used in the folk medicine of northeast of Brazil as sedative and to counteract the effects of snake bite while the inflorescense of *P. arboreum* or a decoction of it leaves are used to prepare a remedy for treatment of hepatic pains [17].

Moreover, Fern et al. [127] also reported that the leaves of *P. arboreum* are taken as a remedy after overeating or in the case of stomach poisoning. A decoction or cold water infusion of *P. arboreum* leaves is used to treat body aches and fevers while the warmed leaves are used as a poultice around joints to relieve arthritic pains and topically around affected area as a treatment for aches, pains and strains. Moreover, the macerated leaves and stems are used as anti-venom.

### 4.52. Piper amalago L.

The leaves of *P. amalago* is traditionally used in Mexico and Brazil to treat heart problems, like hypertension, burns, inflammation, and infections [202], several conditions, including gastro-intestinal and chest pain [203] and to treat stomach pains and various infections [116]. The infusion of *P. amalago* leaves is typically used to relieve intestinal colic, stomachaches, and muscle aches while the alcoholature of the leaves is used during the bath to hydrate and treat the loss of hair. In addition, the infusions of *P. amalago* roots are used as diuretic and against renal stones [202].

Specifically, in Brazil, *P. amalago* is used as an analgesic and anti-inflammatory agent for renal disturbances, such as renal stones [119], and as diuretic, for treating hypertension and renal calculi [17]. In addition, the leaves of *P. amalago* are used to prepare a tea that is used for treating burns; in Puerto Rico and the Caribbean, the chewed leaves of *P. amalago* are put on bleeding cuts [17]; in Trinidad, Puerto Rico and other Caribbean countries *P. amalago* leaf infusions are used as ritual baths or baths to perfume the body [158]. In Mexico, *P. amalago* is used by the Huasteco-Maya tribes against edema, inflamations and as an antipyretic agent. Moreover, the leaves are also used to treat headache, nosebleed, pains, sores, and to prevent miscarriage. Its bark is used to treat cough, gastrointestinal and chest pains. The tender shoots are applied for treating vertigo problems [17].

Fern et al. [127] reported that the leaves and young shoots are discutient, the root is diaphoretic, resolutive and sudorific while the infusion of *P. amalago* flowers is aperitive and vermifuge. Furthermore, the fresh leaves of *P. amalago* are brewed to provide a remedy for coughs while the root is used to treat snake bites

### 4.53. Piper ribesioides Wall. (syn: P. sumatranum (Miq.) C. DC.)

*Piper ribesioides* is widely used in traditional medicine of Indonesia and Malaysia wherein the root is used to treat an illness caused from asthma, diarrhea, and abdominal pain, the leaves treat body wind element abnormality, alleviate chest congestion and excrete phlegm while the flowers have been used to treat urticaria [204].

Fern et al. [127] also reported that *P. ribesioides* is antiseptic and stimulant with both the fruits and EO having diuretic and expectorant effects whilst also improving digestion. The EO of *P. ribesioides* is also reported to be carminative, diuretic and a stimulating expectorant. The immature, dried fruits are applied in the treatment of coughs, bronchitis, sinusitis, throat and genito-urinary infections, poor digestion and amoebic dysentery.

### 4.54. Piper corcovadensis (Miq.) C. DC.

The leaves of *Piper corcovadensis* are used in the North and Northeast regions of Brazil to treat rheumatism in the form of poultice and infusions as well for flu and cough. In the southeast region of Brazil, the roots and branches of *P. corcovadensis* are chewed to relieve toothache due to its anesthetic action on the mucous membrane [205].

### 4.55. Piper futokadsura Siebold

The leaves of *Piper futokadsura* is used in China for the treatment of cardiac arrhythmias and asthma [116].

### 4.56. Piper elongatum Vahl.

*Piper elongatum* is traditionally believed to have astringent, balsamic and stimulant effect, and medicinally used as anti-gonorrhea and to treat constipation and wound [118].

### 4.57. Piper mikanianum (Kunth) Steud

*P. mikanianum* is used to treat amenorrhea and leucorrhea. Specifically, the plant is used in Brazil to cure toothache and stomachache, and possesses diuretic, carminative, digestive stimulant and anti-ulcer effects [118].

### 4.58. Piper medium Jacq.

In Brazil folklore medicine, *Piper medium* is utilized to improve blood circulation, and possesses abortive effects as well [118].

### 4.59. Piper wallichii (Miq.) Hand.-Mazz.

The stems of *Piper wallichii* are medicinally used by for the local people in China to treat rheumatoid arthritis, inflammatory diseases, cerebral infarction and angina by Shi et al. [206]. On the other hand, Tamuly et al. [207] reported that the fruits of *P. wallichii* are medicinally used in India to treat cold fever, cough, and as a uterine stimulant. The fruits are also believed to have cardiac and antibiotic properties. Huyan et al. [208] also added that *P. wallichii* is traditionally used among others for rheumatic arthralgia and lumbocrural pain in addition to the wind-cold dispelling, waist and knee strengthening and kidney-yang invigorating functions. Ding et al. [121] also reported the use of *P. wallichii* in China folklore medicine to promote blood circulation, which are believed to be useful in antiplatelet therapies and in the treatment of thrombotic diseases.

### 4.60. Piper truncatum Vell.

The leaves of *Piper truncatum* is traditionally used in Brazil for the treatment of hypertension [209].

### 4.61. Piper aequale Vahl

The decoction of *Piper aequale* leaves is used by the local people in Brazil to treat rheumatism and inflammation [210].

### 4.62. Piper alyreanum C.DC

According to Lima et al. [211], *P. alyreanum* is used as an immunomodulator, analgesic and antidepressant in folk medicine.

### 4.63. Piper attenuatum Buch.-Ham. ex Miq.

*Piper attenuatum* is used in Indian traditional medicines as rubefacient and its macerated roots are used as diuretic [212]. In addition, the whole plant of *P. attenuatum* is used in the Indian folklore medicine to cure headache and muscular pain [213].

### 4.64. Piper augustum Rudge

In Ecuador and Peru, *Piper augustum* is used in baths, to gain weight, for dental caries, and to clean teeth [177]. In addition, the Waorani tribes of northwest Amazonia use *P. augustum* to prevent tooth decay [117].

### 4.65. Piper darienense C.DC.

According to Durant-Archibold et al. [17] the Guna Indians of Panama used the decoction of *Piper darienense* as bath to alleviate cold and to treat snakebites while the Chocó Indians of Panama use this plant as an effective remedy for treating toothaches and as a fish poison

### 4.66. Piper reticulatum L.

*Piper reticulatum* is traditionally used in Colombia for snakebites [177].

### 4.67. Piper hongkongense C. DC

*Piper hongkongense* is traditionally used in China for promoting blood circulation and thought to be useful in antiplatelet therapies and in the treatment of thrombotic diseases [121].

### 4.68. Piper kadsura (Choisy) Ohwi

*Piper kadsura* is traditionally used in China for promoting blood circulation and thought to be useful in antiplatelet therapies and in the treatment of thrombotic diseases [121].

### 4.69. Piper macropodum C. DC

*Piper macropodum* is traditionally used in China for promoting blood circulation and thought to be useful in antiplatelet therapies and in the treatment of thrombotic diseases [121].

### 4.70. Piper mutabile C. DC

*Piper mutabile* is traditionally used in China for promoting blood circulation and thought to be useful in antiplatelet therapies and in the treatment of thrombotic diseases [121].

### 4.71. Piper puberulilimbum C. DC

*Piper puberulilimbum* is traditionally used in China for promoting blood circulation and thought to be useful in antiplatelet therapies and in the treatment of thrombotic diseases [121]. Identical uses so combine into one list

### 4.72. Piper yunnanense Tseng

*Piper yunnanense* is traditionally used in China for removing blood stasis [214], promoting blood circulation and thought to be useful in antiplatelet therapies and in the treatment of thrombotic diseases [121].

### 4.73. Piper callosum Ruiz & Pav.

The decoction of *P. callosum* leaves has been traditionally used for its diuretic, depurative, and hemostatic properties [119].

### 4.74. Piper conejoense Trel. & Yunck.

The Waorani tribes of northwest Amazonia use *Piper conejoense* to prevent tooth decay [117].

### 4.75. Piper novae-hollandiae Miq.

*Piper novae-hollandiae* is used in Australia folklore medicine to treat gonorrhea and other mucuous discharges [117].

### 4.76. Piper mullesua Buch.-Ham. ex D. Don

As a folk medicine in China, the whole plants of *Piper mullesua* are used to remove blood stasis, treat bleeding, bone fractures, injuries from falls, rheumatoid arthritis, rheumatic arthralgia, acroanesthesia, asthma, colds, stomach aches, abdominal pain, toothaches, swelling and pain of furuncles, dysmenorrhea, menoxenia, empyrosis, and snake and insect bites [214]. In addition, Manandhar [215] reported that the boiled juice of *P. mullesua* fruits is used in India folk medicine to treat coughs and colds.

### 4.77. Piper peltatum L.

Durant-Archibold et al. [17] described the use of infusions of leaves and roots of *Piper peltatum* in Brazil traditional medicine to treat erysipela, malaria, leishmaniasis and hepatitis. Peoples in the Amazon region and other parts of South America traditionally used the infusions of *P. peltatum* root to treat malaria [216]. In Brazil, particularly, infusions of the roots and/or leaves of *P. peltatum* are used in the treatment of malaria, erisipela (a skin ailment caused by *Staphylococcus* spp.), hepatitis, and leishmaniasis [217].

Fern et al. [127] have provided an extensive report on traditional uses of *P. peltatum*. The leaves of *P. peltatum* are said to have anti-inflammatory, antineuralgic, sudorific effects. The leaves of *P. peltatum* are boiled and the water is used as an herbal bath or for washing the skin to reduce high fevers. The heated leaves are tied or wrapped around the head and forehead as a poultice to treat headaches while the leaf infusion is used to treat fevers. Specifically, a decoction of *P. peltatum* leaves is used in Guyana as a purgative to clean out the uterus while the leaves, used together with oil in the form of poultice, is appied to treat bruises and swellings. The people of Guyana also used the leaves of *P. peltatum* to treat abscesses, colds and coughs, haemorrhage, headache, swellings, and for cleaning the womb and tubes. The leaves are also applied externally to the head for a prolonged period of time as an antineuralgic, as a poultice on cuts while the warmed leaves are applied locally to treat conditions such as hernia pain and arthritis pain. Moreover, a combination of macerated leaves and crushed stem together with the leaves of *P. amapaense* is used as a remedy for headache while a mixture of leaves and coconut oil or castor oil is rubbed on painful or swollen joints. In addition, the root is said to be diuretic and partially cooked root is a remedy for uterus pain.

### 4.78. Piper interruptum Opiz (syn: P. ribesoides Wall.)

In Thailand, the stem of *Piper interruptum* is used by the northern and northeastern parts communities as carminative, antiflatulent, and tonic element [136].

### 4.79. Piper guianense (Klotzsch) C.DC.

According to Fern et al. [127], the leaves of *Piper guianense* are mixed with water and crushed, and then small amounts of the infusion are given to infants who have lost their appetite for nursing.

### 4.80. Piper fragile Benth.

Fern et al. [127] has reported on the traditional uses of *Piper fragile* to treat yaws.

### 4.81. Piper coruscans Kunth.

Fern et al. [127] reported that the leaves of *Piper coruscans* are said to be purgative and a decoction of the boiled leaves is consumed as a remedy to treat high fevers. The warmed leaves are used as a poultice to treat children with swollen abdomens.

### 4.82. Piper caninum Blume

In Malaysia, the leaves of *Piper caninum* are chewed by the natives of Malay Peninsular when treating hoarseness while the soaked leaves are used after childbirth [218]. In addition the leaves are also used as antiseptic, or to treat throat ache [110]. Fern et al. [127] has reported that the leaves of *P. caninum* are traditionally used to wash the mother after giving birth or chewed to treat hoarseness.

### 4.83. Piper bantamese Blume

Fern et al. [127] also reported that a poultice consisting of a mixture of the bark of *Piper bantamese* and ginger, clove and nutmeg, can be applied to muscles of arms and legs that are cramped due to cold. In addition, a poultice prepared from a mixture of *P. bantamese* leaves with water is used to relieve headache.

### 4.84. Piper sanctum (Miq.) Schltdl.

Fern et al. [127] also reported that a decoction of *Piper sanctum* leaves can be used to treat indigestion and abdominal cramps. In addition, *P. sanctum* is also used as a stimulant, a local anesthetic, and for the treatment of toothache, stomach affections, and venereal diseases.

### 4.85. Piper sylvestre Lam.

A leaf infusion of *Piper sylvestre* is taken to prevent epileptic attacks while a tea made from the leaves is taken to treat fever and hematuria, and as a diuretic and depurative agents. Moreover, *P. sylvestre* has been used in folk medicine of Mauritius peoples especially for the treatment of asthma and hematuric fever [219].

### 4.86. Piper lanatum Roxb.

*Piper lanatum* has been traditionally used to malaria, toothache, rheumatism, deworming, fever, influenza and ulcers [110].

### 4.87. Piper porphyrophyllum N.E.Br.

*Piper porphyrophyllum* has been traditionally used to treat leprosy, stomachache, skin diseases, postpartum treatment and bone pain [110].

### 4.88. Piper cernuum Vell.

The leaves of *P. cernuum* have been commonly used by rural and urban communities in São Paulo, Brazil, to treat topical pain conditions, such as bellyache and muscle pain, in addition to hepatic and renal complications [220].

### 4.89. Piper cordulatum C. DC.

Traditionally, *Piper cordulatum* is used in Panama as a remedy for skin infections [17].

### 4.90. Piper divaricatum Meyer

In Colombia, *P. divaricatum* is popularly used in traditional medicine as an insecticide [221].

### 4.91. Piper flaviflorum C. DC.

The Dai people of southern China have been using *Piper flaviflorum* as an ethnomedicine to treat dysmenorrhea and tinea. Moreover, the ethnic people in Xishuangbanna located at the southwestern Yunnan, China have been using the leaves and stems of *P. flaviflorum* as an indigenous remedy for inner heat, stomach disorder, and relief of pain and itching, in addition to being consumed as vegetables and spices [222,223].

### 4.92. Piper gaudichaudianum (Kunth) Kunth ex Steud

In the Brazilian Atlantic forest area, the leaves infusion and fresh leaves of *Piper gaudichaudianum* are used as an anti-inflammatory agent as well as to relieve toothache [224]. In addition, the fresh roots are also used as anti-inflammatory agent and to treat liver disorders [225].

### 4.93. Piper hainanense Hemsl.

The part of *Piper hainanense* above the ground has been utilized in Chinese folk medicine to alleviate pain, relieve symptoms of asthma and treat bacterial infections [226].

### 4.94. Piper klotzschianum Kunth.

The leaves of *P. klotzschianum* are used by the local population in Brazil as poultice and infusions to treat rheumatism, flu and cough [227].

### 4.95. Piper miniatum Blume

Various parts of *Piper miniatum* are used in folk medicine of Indonesia and Papua New Guinea, as a spice, a tonic and as a food natural preservative due to its antibacterial activity [228].

### 4.96. Piper aff. pedicellatum C. DC.

The roots and stems of *P. pedicellatum* are used as a carminative in Thai folk medicine [229]. On the other hand, the tribal people of Arunachal Pradesh, India used the roots and stems in treatment of internal body pain, fever, cold and as wild vegetable. Moreover, the shoots of *P. pedicellatum* are boiled with ginger and eaten by the tribal people to relieve the body pain, diarrhea, dysentery and colonic pain [230].

### 4.97. Piper philippinum Miq. (syn. P. kwashoense Hayata)

The Tau (Yami) aborigines on Lanyu Islands in Taiwan used lime and catechu together with the older stems of *Piper philippinum* (in place of pistillate inflorescences of *P. betle*) for betel quid chewing [231].

### 4.98. Piper piscatorum Trel. & Yunck.

Numerous Amazonian ethnic groups in Venezuela and Brazil used the stem and roots of *Piper piscatorum* as a fish poison, toothache remedy, diaphoretic, barbasco and chewing tobacco substitute [232]. The root is chewed to produce a tingling-numbing sensation, which is used to alleviate pain of the teeth and gums. The Piaroa and Hoti Indians of Amazonas in Venezuela also used *P. piscatorum* as local anesthetic in addition to barbasco. When utilized as a barbasco, roots and stems of *P. piscatorum* are macerated on rocks and thrown into slow moving pools of water. Within several minutes stupefied fish can be easily captured [233].

### 4.99. Piper ossanum Trel.

The leaves of *Piper ossanum* are used in Cuban folk medicine as hemostatic, antiseptic, cicatrizing, and diuretic agents [234].

### 4.100. Piper semiimmersum C. DC.

People of Hani in Yunnan Province of China used the roots and leaves of *Piper semiimmersum* to treat bone fracture [235].

### 4.101. Piper submultinerve C. DC.

*Piper submultinerve* has been applied in traditional medicines as antiseptic or anti-inflammatory agents, and for the treatment of herpes simplex and herpes zoster viruses [236].

### 4.102. Piper loretoanum Trel.

*Piper loretoanum* is used by the Chayahuita in Peru in the treatment of leismaniasis, cuts and gastrointestinal ulcer. The dried leaves were reduced in powder and sprinkled on the leishmaniasis-related ulcer. Other than that, the fresh leaves are crushed and applied as a poultice to treat cuts or boiled with water for 2 h and drink to treat internal ulcers [237].

### 4.103. Piper mediocre C.DC.

*Piper mediocre* is used in the treatment of leismaniasis by by the Chayahuita in Peru wherein the dried leaves were reduced in powder and sprinkled on the ulcer [237].

### 4.104. Piper sanguineispicum Trel.

The fresh leaves of *Piper sanguineispicum* are crushed and applied as a poultice on the swelling by the Chayahuita in Peru [237].

### 4.105. Piper taiwanense Lin & Lu

The leaves, old stems and pistillate inflorescences of *Piper taiwanense* are used by the Paiwan aborigines in Taiwan as material for betel quid, in the same way they use the common *P. betle* [238].

### 4.106. Piper trichostachyon (Miq.) C. DC.

The traditional healers of Belagavi region in India utilize *Piper *trichostachyon** similar to *P. nigrum* [239].

## 5. Food Preservative Effects of *Piper* Plants

Food safety is a primary concern of consumers, regulatory agencies, and the food industry. Due to the emergence of antibiotic-resistant microorganisms and increasing concerns associated with the use of synthetic preservatives, there is a greater demand for natural food preservatives [240,241]. Consequently, this need for new antimicrobial agents has generated an interest in new technologies to enhance food safety and quality [242]. One such antimicrobial agent would be spices. Spices and their EOs from different plant sources are considered to be generally recognized as safe (GRAS) [243]. Among spices, *Piper* spp. seems to be very promising as a food preservative to control various food spoilage and pathogenic microorganisms. Particularly, *P. nigrum* (black pepper) is the most important species of this genus due to its pungent principle component, piperine, and its worldwide popularity as a flavoring for food [244]. In this section, we discuss the potential use of different *Piper* species in food applications with special attention given to black pepper.

### 5.1. Antioxidative Activity

Fats and oils present in many foods can easily deteriorate due to oxidation in a chain of reactions in which free radicals are formed, propagated, and finally converted into stable oxygenated compounds. It is these oxygenated compounds that are responsible for off-flavors and other undesirable characteristics [68,245,246]. However, when antioxidants are added to such foods, the shelf life is extended because the lipid peroxidation process is retarded. Synthetic antioxidants such as butylated hydroxyanisole (BHA), butylated hydroxytoluene (BHT), and propyl gallate (PG) have been used as food additives since the beginning of the 20th century in food industries, but there are some arguments about the safety and possible adverse effects of these substances [245]. The species *P. nigrum* (black pepper) is used as a spice in many countries of the world due to the presence of piperine, which may contribute its value as a food additive. The antioxidant activities of black pepper EO and oleoresins were evaluated and compared to those of BHA, BHT, and PG synthetic antioxidants [68]. The authors found that both the EO and oleoresins showed strong antioxidant activity in comparison with synthetic oxidants. The results of this study demonstrated that black pepper can be used as an easily accessible source of natural antioxidants and as a safe food preservative. It has been shown that the addition of black pepper powder in cottage cheese at a 1.0% level considerably improved the flavor and aesthetic quality of the product. In addition, the black pepper powder extended the shelf life of the product from 8 to 14 days without affecting sensory and textural properties in comparison to the control sample [247]. Similarly, the antioxidant properties of EOs from some other *Piper* spp. such as *P. betle* were evaluated by Prakash et al. [55]. The results of the study demonstrated that *P. betle* EO exhibited strong antioxidant potential as its half maximal inhibitory concentration (IC_50_) value (3.6 μg/mL) was close to that of ascorbic acid (3.2 μg/mL) and lower than that of the synthetic antioxidants such as BHT (7.4 μg/mL) and BHA (4.5 μg/mL). This finding is further supported in a study by Nakatani et al. [248] which revealed that phenolic amides from *Piper* spp. possess significant antioxidant activities that are more effective than the naturally occurring antioxidant, alpha-tocopherol. For example, one amide, feruperine, exhibits antioxidant activity as high as that of the synthetic antioxidants, BHA and BHT. These studies thus indicate that *Piper* spp. has special merits with regard to enhancement of the shelf life of food products.

Phenolic compounds appear to be responsible for the antioxidant activity of black pepper [245]. The antioxidant mechanisms of black pepper extracts may be attributed to a strong hydrogen-donating ability, metal chelating ability, and the effectiveness of these extracts as good scavengers of hydrogen peroxide, superoxide, and free radicals. Black pepper oleoresin (BPO) is a source of biologically active compounds that are responsible for food preservation [249]. However, low stability and solubility in water are two limiting factors for BPO, with some of its components being sensitive to light, heat and oxygen which compromise BPO’s chemical and sensorial characteristics [250,251]. Recently, Ozdemir et al. [252] developed BPO-BCD (β-cyclodextrin) inclusion complexes using the kneading and freeze drying methods which were more effective in keeping BPO active properties for longer periods and thereby delaying oxidative reactions and the growth of microorganisms.

### 5.2. Antimicrobial Activity

Black pepper (*P. nigrum*) volatile oil has been proven to have antimicrobial activity [253]. The phenolic compounds of black pepper are believed to be responsible for this antimicrobial activity by damaging the membranes of bacteria restricting their growth [254]. Black pepper has also been shown to exhibit antibacterial activity with reported minimum inhibitory concentrations (MICs) of around 50–500 ppm and inhibition of Gram-positive bacteria such as *Staphylococcus aureus*, followed by *Bacillus cereus* and *Streptococcus faecalis.* In addition, black pepper has demonstrated inhibition against some Gram-negative bacteria such as *Pseudomonas aeruginosa* [254]. Black pepper EO is composed primarily of monoterpenes and sesquiterpenes [255]. This EO has also been used to inhibit the growth of microorganisms such as *Vibrio cholerae*, *Staphylococcus albus*, *Clostridium diphthereae, Shigella dysenteriae, Streptomyces faecalis, Bacillus* spp. and *Pseudomonas* spp. in addition to suspending the growth and production of aflatoxins produced by *Aspergillus parasiticus*. These effects are due to chemical constituents such as piperazine, piperanine, piperidine A and piperolein B [255]. To enhance the effect of EOs, these compounds can be encapsulated. In a recent study, Rakmai et al. [256] demonstrated that cyclodextrins can be used as an encapsulating agent that can protect the active compounds. These authors found that following encapsulation in hydroxypropyl-β-cyclodextrin (HPβCD), the antibacterial activity of black pepper oil improved by a factor of four against *S. aureus* and *E. coli*. Akthar et al. [257] investigated the antimicrobial activity of leaf extract of *P. nigrum* against the foodborne pathogenic bacteria *S. aureus, E. coli, Salmonella typhimurium*, and *P. aeruginosa* and fungi (*Aspergillus* spp. and *Candida albicans*). The methanol extract exhibited greater antimicrobial activity against the selected bacterial and fungal strains. The MIC results showed that ethanol, methanol and petroleum ether leaf extract of *P. nigrum* inhibited the growth of *S. aureus* and *E. coli* at a concentration of 12.5 mg/mL. The MIC values for ethanol, methanol and petroleum ether leaf extract of *P. nigrum* for *C. albicans* were at a concentration of 25.0 mg/mL. Similarly, the minimum bactericidal and fungicidal concentrations of all the tested solvent leaf extract of *P. nigrum* plants ranged between 12.5–50.0 mg/mL for all tested strains. Table 28 lists the efficacy of black pepper extracts and black pepper EOs against food spoilage and foodborne pathogens. In addition to *P. nigrum*, other spp. of *Piper* plants have also been studied. One recent study demonstrated the antimicrobial activity of nanoemulsion of betel leaf (*Piper betle* L.) EO. The formulated nanoemulsions had a MIC of 0.5–1.25 µL/mL and a minimum bactericidal concentration (MBC) of 1–2.5 µL/mL against five strains of Gram-positive (*S. aureus, B. cereus*) and Gram-negative (*E. coli, Klebsiella pneumonia, P. aeruginosa*) bacteria. Such formulated nanoemulsions can thus serve as natural antimicrobial agents for food systems [258]. Similarly, Nouri and Nafchi [259] observed the antimicrobial activity of betel leaf extract against selected Gram-positive and Gram-negative bacteria.

The abovementioned studies suggest that not only the seeds but also leaves of *P. nigrum* could be potential candidates for developing new antimicrobial agents for a wide range of pathogenic bacteria and fungal strains. For example, Pauli [260] reported the mechanism of action of the components present in betel leaf EO compounds such as sesquiterpene, monoterpenes, and phenylpropanes where these compounds are similar to other terpenes and phenolic compounds involved in the destruction of the bacterial cytoplasmic membrane that leads to cellular material coagulation. Another study by Basak and Guha [261] describes the effect of betel leaf EO on spore inactivation and the cell viability of *Aspergillus flavus* and *Penicillium expansum* and its antifungal activity in raw apple juice. These authors reported that the cells of *A. flavus* and *P. expansum* lose viability when treated with EO. Since spore inactivation or the inhibition of spore germination is necessary in order to restrict fungal infection and mycotoxin production in food, the use of *P. betle* leaf extract as a natural antifungal agent in food systems is very promising.

Our literature review showed that *Piper* spp. could be used as a natural antioxidant and antimicrobial agent in food preservation. However, *Piper* extract could affect food organoleptic characteristics. Therefore, careful selection of appropriate concentrations of this extract with regard to the sensory and compositional status of the food system to which it is applied is required in order to gain consumers’ acceptability.

## 6. Antiparasitic Activities of *Piper* Species

Numerous *Piper* species are used in traditional medicine to treat parasitic diseases [41,266]. In general, we found 26 species that demonstrated antiparasite potentialities (Table 29), collected in different geographical areas such as Africa [267,268], Asia [266,269,270] and Latin American [41,170,271]. In particular, the most assayed plants were *P. aduncum*, *P. betle* and *P. longum*. In addition, extracts and fractions have been studied; however, a special characteristic of this genus is that many species present volatile compounds. In this sense, it is interesting to comment that potentialities of EOs from *Piper* plants have been also widely explored (Table 29).

Numerous reports on protozoa and helminths were retrieved from the search, as well the use of different in vitro or in vivo models. Among protozoa, the main parasites targeted were *Plasmodium falciparum*, *Trypanosoma cruzi* and *Leishmania* spp. (Table 29), causal agents of malaria, Chagas disease and leishmaniasis, respectively, and represent the more important protozoal diseases with respect to the mobility and mortality caused by these agents. Promising antiplasmodial activity was reported for extracts from *P. capense* [268], *P. cumanense* [170] and *P. nigrum* [272], with IC_50_ values of 2 µg/mL, 7 μg/mL and 12.5 μg/mL, respectively. In contrast, antitrypanosomal and antileishmanial potentialities were prominent in the EO from *P. aduncum* [45] with IC_50_ values of 2.8 μg/mL, 12.1 μg/mL and 9 μg/mL in cell-derived and metacyclic trypomastigotes, as well as intracellular amastigotes, and *P. malacophyllum* with 17 μg/mL against trypomastigotes [273] of *T. cruzi*; while oil from *P. demeraranum* with IC_50_ value of 15 µg/mL [93] and *P. hispidum* with 4.7 µg/mL value [274] against *Leishmania amazonensis*.

In addition, other *Piper* species have been evaluated and showed no relevant activity, for example: extracts of *P. sarmentosum* [301], *P. umbellatum* and *P. holtonii* [302] against *P. falciparum*; *P. cubeba* against *L. amazonensis* [303]; as well as *P. nigrum* and *P. sarmentosum* on *Toxoplasma gondii* [284].

Most of the studies were performed using in vitro models, so in vivo experimental approaches are needed to demonstrate the *Piper* species potentialities. In this sense, *P. betle* has been studied on different animal models infected with parasites. For example, using experimental infections of *Giardia lamblia* in Mongolian gerbils [304], a significant decline in cyst shedding in treated gerbils with the aqueous extract was appreciated; *Neospora caninum*-infected mice, treated also with *P. betle* extract, showed reduced mouse clinical scores and increased survival rates [270]; and finally, *T. gondii* in vivo infection resulted in 100% mouse survival after treatment with *P. betle* extract [284].

Scarce studies were found about antiparasite mechanisms of action for either extracts or EOs from *Piper* species. This observation could be logical since both products are complex mixtures of compounds, which could present several activities, act on multiple targets and cause the death of parasite by different mechanisms. Mitochondrial dysfunction and apoptosis have been the events more often described, as the evidences of *P. betle* on *Leishmania* [279,280]. In parallel, indirect mechanisms could contribute to antiparasite effect, acting on host cell. In this sense, herbal preparation of *P. longum* displayed only in vivo effects, probably by enhancement of the host immune system that contributed to the recovery of animals from the giardial infections [293].

## 7. Biological Activities *Piper* Plants

Chronic illnesses such as type II diabetes, cardiovascular diseases, cancer and neurodegenerative disorders are the primary causes of disability and death all over the world. However, most of these diseases can be preventable or at least be delayed by lifestyle modifications, particularly by dietary changes [305]. It has been demonstrated that *Piper* species possess therapeutic and preventive potential against several chronic disorders. Among the functional properties of *Piper* plants, the antiproliferative (Table 30), anti-inflammatory (Table 31), and neuropharmacological activities (Table 32 and Table 33) of the extracts and extract derived bioactive constituents are thought to be key effects for the protection against chronic conditions. The following sections reviewed the abovementioned functional biological activities of different *Piper* extracts/active components based on preclinical in vitro and in vivo studies, besides clinical studies (Table 34).

We examined all phytochemical papers published in English by using PubMed, Google Scholar, Elsevier and Science Direct databases. For reviewing functional properties of *Piper* plants, *in vitro/in vivo* studies from the year 2013 to 2018 have been collected. The part which summarizes clinical trials has focused on anxiolytic properties of specific kava extracts between 1991 and 2013 and has only covered studies with monotherapy.

### 7.1. Antiproliferative/Anti-Cancer Properties

According to recent world health statistics published by WHO, cancer caused 9.0 million deaths in 2016 [306]. It is expected that this number will increase in coming years unless successful treatment and/or preventive strategies can be developed [307]. Mankind has traditionally used plants to self-treat health problems throughout history; nowadays researchers focus on understanding the action mechanism of plant-based chemicals and identifying effective sources for new drugs [308]. Naturally occurring plant-based chemicals serve alternative approaches regarding chemotherapy and chemoprevention with relatively fewer side effects. In the present section, in vitro and in vivo studies involving evaluation of the anti-proliferative, anticancer and chemopreventive effects of both extracts and bioactive constituents from *Piper* plants were reviewed.

Dried samples of *P. longum* were extracted with several solvents including hexane, benzene, acetone, ethyl acetate, ethyl alcohol, chloroform, and water [309]. The anticancer activities of the various extracts were evaluated in several different human carcinoma cell lines (A549 lung, THP-1 leukemia, DU-145 prostate, IGR-OVI-1 ovary, and MCF-7 breast cancers). The cytotoxic activities of the extracts (100 μg/mL) quantified by sulforhodamine B assay on those cell lines. All extracts produced growth inhibition in THP-1 (76–90%); while hexane and benzene extracts showed cytotoxic effect (>80%) on the growth of all cell lines. It was noted that standard anticancer drugs exhibited 51–67% cytotoxicity in comparison with control groups in the study. Acetone, benzene and hexane extracts inhibited cell cycle 43, 41 and 63%, respectively in A549 cell line and resulted in increased sub-G1 DNA fraction population. The effectiveness of extracts was also studied on Wistar rats having AlCl_3_-induced hepatotoxicity. Accordingly, aqueous, chloroform and ethyl alcohol extracts showed protective effect on liver 65, 71 and 64%, respectively against peroxidative damage. In another study, ethanol extract of fruits of *P. longum* were examined by using in vitro and in vivo models to clarify its efficacy and safety [310]. Interestingly, the results indicated that treatment by extract selectively induced cell death in carcinoma cells including HCT116 colon, BxPC-3 pancreas and T cell leukemia; however extract was not effective on normal colon epithelial cells. Findings were supported by Annexin V Binding Assay confirming that ethanolic extract induced cell death by caspase-independent apoptosis. As animal models, immunocompromised mice were studied, and administered with ethanolic extract at a dose of 50 mg/kg/day routinely for 6 weeks. In treatment group, the growth of colon cancer tumors was suppressed without any toxic effect.

In vitro and in vivo studies demonstrated that *P. umbellatum* species can be a promising source for anticancer agents. In the study of Iwamoto et al. [311], the extraction of milled fresh leaves of *P. umbellatum* was prepared by dichloromethane. Total growth inhibition was achieved in several different human carcinoma cell lines (U251, MCF-7, NCI-H460, UACC-62, PC-3, 786-0, NCI-ADR/RES and OVCAR-3) with relatively small effective doses (6.8 and 14.9 µg/mL). As the concentration of total growth inhibition was determined as 144.6 µg/mL for nontumor cell line, cytotoxic effect of dichloromethane extract seem to be selective for tumor cells. In the same study, Balb/C mice with Ehrlich solid tumor were treated with 100, 200, and 400 mg/kg of the extract by oral route. They found that the sizes of the tumors were reduced 38.7 and 52.2% in 200 and 400 mg/kg extract treatment groups, respectively, without toxicity. Similar effects were also shown by another study in which crude extract of *P. tuberculatum* treated as a test material [10]. The cytotoxic activity IC_50_ of the crude extract was found to be 10 μg/mL in SF-295 cells and 4.3 μg/mL in the HCT-8 cell line. Test material was also examined in Balb/C nude mice (100–200 mg/kg/day); as a result, a hollow fiber assay showed that crude extract inhibited cell proliferation in those SF-295 and HCT-8 cell lines by 24.6–54.8%, respectively, without any sign of toxicity.

In the study of de Souza Grinevicius et al. [312], the ethanolic extract of *P. nigrum* fruits was evaluated for its antitumor activity in Balb/C mice model of Ehrlich carcinoma and breast (MCF-7) and colon cancer (HT-29) cell lines. The dytotoxicity of the extract was determined in carcinoma cells by 3-(4,5-dimethylthiazol-2-yl)-2,5-diphenyltetrazolium bromide (MTT) assays and effective concentrations (EC_50_) of 27.1 ± 2.0 µg/mL in MCF-7 and 80.5 ± 6.6 µg/mL in HT-29 were found. In vivo studies exhibited that extract treatment elicited 60% decrease of tumor growth and 76% increase of survival time together with stimulation of apoptosis which collectively resulted in inhibition of Ehrlich carcinoma in mice. Furthermore, Bax and p53 protein expression levels were increased, whereas Bcl-xL and cyclin A expressions were inhibited with the treatment of extract; so, cell cycle arrest at G1/S and increased rate of apoptosis were observed. The following year, a more effective extraction method—Supercritical Fluid Extraction (SFE)—was introduced; briefly a high-pressure unit was used to obtain extract from the *P. nigrum* fruits, specifically Bragantina cultivar [313]. SFE produced an extraction product, namely SFE200, having a minimum ratio of monoterpenes/ sesquiterpenes and a relatively higher concentration of piperine. SFE200 showed higher cytotoxic effects on MCF-7 cells than conventional extracts. In vivo studies validated the effect of this special extract for which Ehrlich tumor-bearing mice were selected as model organism. Treatment by SFE200 at a dose of 10 mg/kg/day presented more significant tumor growth inhibition and increased the time for survival comparing with conventional extract treatment group. It was also noticed that SFE200 decreased cell number founding at S phase, probably due its apoptotic effect by arresting G2/M phase of cell cycle.

In contrast to the above study where the piperine content was increased, the biological effects of piperine-free *P. nigrum* (PFPE) extract were investigated in the study of [314]. Several cancer cell lines and two normal cell lines were subjected to different doses of PFPE to determine its cytotoxicity and selectivitiy index. Among cell lines, PFPE showed higher cytotoxic effect on MCF-7 breast carcinoma cells (IC_50_: 7.45 µg/mL) together with better selectivity. Flow cytometry analysis demonstrated that PFPE induced apoptosis on MCF-7 carcinoma cells in a dose-dependent manner. The expressions of apoptosis-associated proteins were also examined by western blot, and treatment by PFPE with IC_50_ concentration upregulated p53 and cyt C and downregulated topoisomerase II. Similar results were also obtained for in vivo studies. When rats with mammary tumorigenesis were administered with 100 mg/kg PFPE, tumor grew much smaller as compared to non-treated control group. The same research group published one more article in the following year that clarifies the action mechanisms of PFPE on breast cancer [315]. *N*-nitroso-*N*-methylurea-stimulated mammary carcinoma rat models were administred with different concentrations of PFPE (100, 200 and 400 mg/kg). The most significant tumor suppression activity was obtained in 400 mg/kg treated groups and suppression scores were 2.18-fold and 1.75-fold for 100 and 200 mg/kg treatment groups, respectively. Moreover, PFPE decreased the expression level of vascular endothelial growth factor (VEGF), E-cad and c-Myc proteins as compared to control groups, while protein level of p53 was significantly increased. Western-blot analyzes over breast cancer cell line MCF-7 exhibited similar results for those proteins but expressions of proteins were not changed significantly in PFPE-treated ZR-75-1 cell line. In conclusion, PFPE was found to be a promising extract for the suppression of uncontrolled proliferation of cancer cells through increasing the expression level of p53 and by decreasing the level of c-Myc.

Not only *Piper* extracts but also major bioactive components of *Piper* plants, mainly piperine, piperlongumine, and flavokawain B (FKB), have been investigated as potential anticancer agents in several studies. Piperine is a kind of pungent alkaloid found in *P. nigrum* and *P. longum* species [316]. Besides its anticancer effectw, it has been reported as an anti-inflammatory, neuroprotective and cardiovascular protective agent [317,318,319,320]. Furthermore, its anti-angiogenic characteristic was shown for the very first time in 2013 [321]. According to this study, piperine inhibited the proliferation of HUVEC cells via inhibiting G1/S transition, cell migration and tubule formation without toxic effect. As compared to control groups, 100 μM piperine treatment inhibited the activation of PKB through phosphorylation from the residues of Ser 473 and Thr 308 in HUVEC cells, whereas it had no effect on protein expression of TRPV1. In ex vivo test design, inhibition of tubule development was achieved in the treatment group of rat aorta angiogenesis model.

In the study of Yaffe et al. [322], piperine treatment on HRT-18 human rectal adenocarcinoma cells inhibited metabolic activity in a dose-dependent manner. Flow cytometric analysis represented that piperine also induced apoptosis. However, in this study it was found that apoptosis induced by piperine was mediated by the increase in reactive oxygen species (ROS) production; particularly, hydroxyl radicals. Treatment of cells with *N*-acetylcysteine, a well-known antioxidant, suppressed apoptosis in piperine-treated cells.

Piperine exhibited its antitumor effects by inhibiting *HER2* mRNA expression l in *HER2*-overexpressing breast carcinoma cell lines [323]. In piperine-treated cells, apoptotic cell death increased by caspase-3 activation and PARP cleavage. Piperine also reduced cell migration by interfering with several signaling pathways including Akt, ERK1/2 and p38 mitogen-activated protein kinase (MAPK). Piperine demonstrated similar effects on vascular smooth muscle cells (VSMCs) found within blood vessels by inducing arrest at cell cycle and suppressing the activation of the MAPK through phosphorylation [324]. It reduced the BB and platelet-derived growth factor (PDGF)-induced uncontrolled cell proliferation by changing the expression of p27^Kip1^, cell cycle proteins including cyclin E, cyclin D and PCNA.

Piperine was found to be an anticancer agent against osteosarcoma cells including HOS and U2OS cell lines [325]. It showed growth inhibitory action in both osteosarcoma cell lines but relatively weaker inhibitory effect in normal hFOB cells. By piperine treatment, the cell population found in G2 phase was enhanced while the cell population in G1 phase was reduced, however, no changes in S phase population were observed. Therefore, piperine exerted its effect by arresting cell cycle at G2/M phase. Additionally, piperine represented its inhibitory properties on cell migration and invasion by increasing the expression of TIMP-1/-2 and by down-regulation of MMP-2/-9.

In another study, it was reported that piperine has potent antitumor activity against triple-negative breast cancer (TNBC) [326]. Both in vitro and in vivo analyses presented its selective inhibition effect on cell growth. Controlled cell death induced by piperine was shown through the mitochondrial pathway and through the suppression of Akt activation in breast cancer cells. In a recent study, piperine exhibited antitumor property against human ovarian cancer cell line (A2780) [327]. Cell viability was reduced in piperine-treated cancer cells whereas no significant changes were seen in normal cell line. The proportions of phosphorylated forms of JNK and p38 MAPK protein to non-phosphorylated forms were increased by piperine treatment in dose-dependent manner suggesting that JNK and p38 MAPK mediate intrinsic apoptotic pathway in ovarian cancer cells.

*P. methysticum*, also known as kava-kava, is another *Piper* species rich in three kinds of chalcones, namely flavokawain A, B, and C. Among these compounds, FKB has been reported as promising anti-inflammatory, antinociceptive, and as well as antitumorigenic agent toward several cancer cell lines [328,329,330,331]. Antitumorigenic effects of FKB were evaluated in both 4T1 cell line and Balb/C mice [332]. Cytotoxicity of FKB toward 4T1 cell lines was determined by MTT assay and cell growth was inhibited in a dose-dependent manner. Additionally, FKB interfered with cell cycle and raised the cell population in the SubG0/G1 phase as compared to control groups. Findings from in vivo study also showed the inhibition of tumor growth in 50 mg/kg FKB treated mice during 28 days. Interestingly, FKB treatment increased CD4/CD3 T-cell and CD8/CD4 T-cell populations significantly resulting in positive effect on immune system of test animals. Apart from that, FKB treatment reduced the cancer formation in another side of body including lung, liver, and spleen significantly while the number of colonies was higher in the non-treated group. Antiangiogenic effect of FKB has been investigated in 1.0, 2.5, and 5.0 μg/mL treated HUVEC cell lines [333]. Tube-like structures were counted manually and accordingly branch formations were found to be 38.44 ± 3.95 and 25.44 ± 5.69 in 2.5 and 5.0 μg/mL treated groups, respectively. In the same study, zebrafish embryos were subjected to FKB as test model. Up to 5.0 μg/mL FKB treatment, there were no toxicity signals. However, 10 μg/mL inhibited the formation of subintestinal veins and showed toxic effect. Therefore, the best inhibition rate was observed in 2.5 μg/mL FKB-treated group. These reports for promising anticancer activities of FKB provided the inspiration for the synthesis of potentially more effective FKB derivatives. Accordingly, in the study of Abu Bakar et al. [334], 23 FKB analogs were synthesized. The cytotoxic effects of these compounds were evaluated in two breast cancer cell lines (MCF-7 and MDA-MB-231). Five synthetic derivates showed notable cytotoxic activities in MCF-7 cell line (IC_50_ 5.5–5.9 µg/mL). Piperlongumine, also known as piplartine, is another bioactive component, an amide alkoloid found in the fruits of *Piper* species, particularly, in *P. longum.* While it was isolated in 1961 for the first time, its pharmacological properties including neuroprotective, anti-atherosclerotic and antimicrobial, have been elucidated in the past decade [335,336]. Although there were two in vitro studies showing its potent antiproliferative effects on both prostate and ovarian cancer cell lines in 2013 and 2014, the mechanism based detailed studies for its anticancer properties were published recently. The antiproliferative effects of PL at low micromolar concentrations on LNCaP and PC-3 human prostate cancer cells was found to be associated with reduction in the protein level of androgen receptor AR, which is key element of oncogenic precursor [337]. PL also inhibited cell growth in human ovarian cancer cells with IC_50_ values in the ranges of 6 to 8 µM in three different cell lines by G2/M cell cycle arrest [338]. In those cells, intracellular ROS levels were increased by PL treatment in dose dependent manner which consequently resulted in apoptosis.

Pro-apoptotic effect of PL treatment was also seen in an experimental design with human cholangiocarcinoma cell lines in concentration dependent manner [339]. In this study it was shown that the activation of caspase-3, PARP, JNK-ERK as well as stimulation of ROS accumulation underlies the action mechanism of PL. Furthermore, PL treatment induced p21 expression at protein level resulting cell cycle arrest at G2/M phase in cholangiocarcinoma cell lines (KKU-055, KKU-100, KKU-139, KKU-213, and KKU-214). Similar anticancer potency was seen in 5 to 15 μM PL-treated pancreatic, lung, kidney, and breast cancer cell lines [340]. Protein analysis showed that PL suppressed expression of Sp1, Sp3, Sp4, and Sp-regulated genes containing cyclin D1, survivin, cMyc, EGFR and hepatocyte growth factor receptor (cMet). Apoptosis was shown to be ROS-mediated and both were found to be attenuated after co-treatment with glutathione.

Recently Machado et al. [341] reported that PL did not show a genotoxic effect on plasmid DNA and CT-DNA assessed by cleavage activity and circular dichroism assays. However, studies on HCT 116 cells exhibited ROS-mediated apoptotic activity of PL.

In the study of da Nóbrega et al. [342], nineteen PL analogues have been synthesized by using the 3,4,5-trimethoxycinnamic acid-like starting material, and their cytotoxic potencies were screened in U87MG glioblastoma cell line. Among these test compounds, *(E)*-benzhydryl 3-(3,4,5-trimethoxyphenyl) acrylate, which has two aromatic rings in the side-chain, presented the best inhibition effects on viability of U87MG cell line in a dose dependent manner by oxidative and apoptotic processes. In this study, the biosafety of this compound was also assessed by sister chromatid exchange and 8-hydroxy-20-deoxyguanosine assays in human peripheral blood cells. Results indicated that this synthesized compond would be promising an agent selectively cytotoxic and genotoxic to cancer cells as well as with strong bioavailability. Taken together, some extracts and active constituents of *Piper* plants can be promising as antiproliferative and chemopreventive agents on which more in vivo studies as well as well-designed clinical trials are crucially needed.

### 7.2. Anti-Inflammatory Properties

Inflammation can be considered as a defense mechanism of living organisms to various stimuli involving pathogens, toxic compounds, and many environmental stress factors [343]. Normally, this highly regulated and self-limiting phenomenon promotes the healing process. However, uncontrolled and pro-longed process, called as chronic inflammation, may cause sustained activation of immune cells resulted in high amount of cytokine release [344,345]. This persistent response potentially leads to pathological disorders such as autoimmune diseases, diabetes, heart diseases, cancer and various neurodegenerative disorders. Understanding the basis of inflammation and characterization of novel anti-inflammatory agents may serve promising therapeutic approaches for the prevention and/or treatment of chronic inflammatory diseases.

Functional biological activities of *Piper* plants against inflammation were shown by numerous in vivo and in vitro studies. In the study of Tasleem et al. [346], ethanol and hexane extract of *P. nigrum* and one of its bioactive compound, piperine, were shown to be effective against carrageenan induced-paw edema in Swiss albino mice. Piperine treatment inhibited edema growth at all doses (5, 10 and 15 mg/kg). While the hexane extract was effective at 5 and 10 mg/kg treatment groups, the best inhibition score was obtained at 10 mg/kg treated group for 60 min. The ethanol extract also exhibited good inhibition profile at 10 mg/kg treatment group as compared to control groups. However, all inhibition scores were less than the standard drug, diclofenac sodium.

To elucidate the effects of *P. nigrum* ethanolic extract (PNE) on airway inflammation model, Balb/C mice were induced with ovalbumin (OVA) and administered orally with 200 mg/kg PNE [347]. PNE decreased the size of inflammation related cells such as eosinophils, goblet, and mast cells in bronchoalveolar lavage fluid (BALF). Although OVA induced the cytokine productions, PNE regulated balance of T cells responses by decreasing IL-1β, IL-4, IL-17A, and TNF-α cytokine levels in both BALF and lung homogenate. Thus, PNE demonstrated significant potency on inhibition of inflammation.

Laksmitawati et al. [348], investigated the anti-inflammatory activity of *Piper crocatum* extracts prepared by maceration technique using 96% ethanol. To screen the toxic effect, murine RAW 264.7 macrophage cell line was treated with several doses of extract (0–500 μg/mL) and subjected to MTS (3-(4,5-dimethylthiazol-2-yl)-5-(3-carboxymethoxyphenyl)-2-(4-sulfophenyl)-2*H*-tetrazolium) assay. In 150 μg/mL, and higher treatment concentrations, cell viability was found to be significantly affected as compared to control groups. TNF-α level was decreased significantly at 50 μg/mL treated cell group while each treatment groups (10, 50 and 75 ug/mL) showed decreased IL-1β levels as compared to control groups. Extract at 10 and 50 μg/mL concentrations exhibited significant reduction in IL-6 levels and the lowest NO level was obtained in the 50 μg/mL treatment group.

In the study of Reddy et al. [349], the analgesic and anti-inflammatory activity of a hydroalcoholic extract (70% ethanol and 30% distilled water) of *P. betle* leaves (HEPBL) were demonstrated. Wistar rats and Albino mice were administered different concentrations of HEPBL according to OECD guideline no. 425 to detect non-toxic treatment concentrations. Treatments at 100 mg/kg and 200 mg/kg exhibited significant analgesic properties in both animal groups. Interestingly, HEPBL decreased the pain by both central and peripheral mechanisms. According to literature nonsteroidal anti-inflammatory drugs inhibit the pain just peripherally, but narcotic analgesics reduce the pain both centrally and peripherally. HEPBL also showed anti-inflammatory effect against the carrageenan induced paw edema model and cotton pellet-induced granuloma model. Compared to control group, HEPBL significantly inhibited the paw edema growth in the 50, 100, 200 mg/kg treatment groups in a dose dependent manner. The most effective dose was shown to be 200 mg/kg with a 79.73% reduction score after 3 h. Similarly, reduction in the dry weights of granuloma was reported as 57.49% in a 200 mg/kg treated group as compared to control groups.

A standardized dichloromethane extract (SDE) of *P. umbellatum* leaves exhibited anti-inflammatory activity on carrageenan-induced paw edema and peritonitis models [311]. Balb/C mice were administered with carrageenan solution (2.5 mg/mL, 40 µL/animal) to induce inflammation, and after one hour 100, 200, and 400 mg/kg of SDE were treated. The paw volume was measured at certain intervals. SDE treatment demonstrated inhibitory effect on paw edema during first phase (24 h) without toxicity. Interestingly, increased leukocytes migration promoted second phase of inflammation. However, all concentrations of SDE inhibited inflammation at 48 h. Taken together, *P. umbellatum* SDE might be promising anti-inflammatory agent.

Recently, Finato et al. [350] investigated the anti-inflammatory effect of crude extracts of *P. gaudichaudianum*, *P. arboreum*, *P. umbellata*, *P. fuligineum* and, *Peperomia obtusifolia* in LPS-induced peripheral blood mononuclear cells (PBMCs). Cytotoxic concentrations of extracts were determined by MTT assays and IC_50_ (mg/mL) values of 0.55, 2.19, 1.56, 0.24, and 2.12 were found for each extract, respectively. These specified doses were treated to cells with and without an inflammatory stimulus; after 24 h incubation, cell-free supernatants were subjected to cytokine analysis. All extract exhibited differential inhibitory effect on proinflammatory cytokines such as IL-1β, IL-6, IL-8, IL-10 as well as TGF-β1 and TNF-α.

In addition to *Piper* extracts, bioactive components of *Piper* plants were also evaluated in terms of their anti-inflammatory activities. In the study of Umar et al. [351], piperine exhibited anti-inflammatory effects in collagen-induced arthritis model of Wistar rats that were administered with 100 mg/kg of piperine for 21 days. Effectiveness of piperine on production of inflammatory mediators (IL-1β, TNF-α, IL-10 and PGE_2_) were assessed by ELISA and by Griess assay for measurement of NO. Piperine treatment reduced the NO production in piperine group as compared to control group. Moreover, administration of piperine inhibited the production of pro-inflammatory mediators, IL-1β, TNF-α and PGE_2_, but increased the level of IL-10.

In the study of Ying et al. [352], the effects of piperine at non-toxic doses (10, 50 or 100 μg/mL) were investigated in LPS induced RAW264.7 cells. As compared to LPS treatment, piperine reduced the production of PGE_2_ and NO significantly in dose-dependent manner. Additionally, protein expression levels of COX-2 and inducible NOS were suppressed significantly at 50 or 100 μg/mL, while their mRNA expression levels were decreased at each treatment group. Piperine exhibited the inhibitiory effect on both TNF-α production and its mRNA expression in dose dependent manner. Moreover, piperine treatment caused a significant decrease in the activation of NF-ĸB by suppressing IĸBa phosphorylation and a reduction in the nuclear translocation of p65 subunit.

Additionally piperlonguminine (PL), a bioactive molecule of *P. longum* demonstrated protective effects against endothelial barrier disruption induced by LPS-stimulated-proinflammatory responses in cell and animal models [353]. Barrier protective effects of PL (between 5–40 µM doses) have been investigated by measuring endothelial cell permeability, migration and monocyte adhesion assays as well as by measuring the activation of proinflammatory proteins in LPS-induced HUVEC cells and in mice. PL reduced the migration of monocyte cells to HUVECs, expression of CAMs (cell adhesion molecules) thus demonstrated a protective effect against LPS-stimulated disruption of endothelial barrier. It was also demonstrated that PL inhibited LPS-induced production of IL-6 and TNF-α through inhibiting the activation of NF-ĸB and ERK 1/2 by LPS.

Kava *(P. methysticum)* extracts are commonly used as a preventive strategy for the treatment of various mental disorders such as anxiety and nervous tension. There are also experimental evidences substantiating anti-inflammatory effects of this species or its active components [354,355]. In the study of Kwon et al. [356], one of the active chalcone constituents of kava extracts called flavokawain A (FKA) has been investigated for its anti-tumor and anti-inflammatory activities in LPS-induced RAW 264.7 macrophage cell line. Accordingly, FKA pretreatment resulted in reduced production of LPS-induced NO and PGE_2_ levels compared to LPS-treated control. Besides, protein and mRNA expression levels of iNOS and COX-2 were also suppressed by FKA treatment in a dose-dependent manner. FKA also inhibited LPS-induced activation of NF-kB. Moreover, FKA downregulated LPS-induced pro-inflammatory cytokines, including IL-6, IL-1β and TNF-α at both protein and mRNA level.

Another kava component, kavain and its structural analog kava-241 obtained synthetically also investigated for their anti-inflammatory properties [357]. In this study, RAW 264.7 cells were exposed to 100–300 μg/mL of kava and kava-241 after 0.1 μg/mL of LPS stimulation. While kava reduced LPS-induced TNF-α production by 75%, kava-241 resulted in a more prominent inhibition (85%) on the same parameter as compared to control group. Moreover, kava-241 showed less cytotoxic effect than kava. In vivo studies demonstrated that in the periodontitis model of DBA1/BO male mice, 40 mg/kg kava-241 administration resulted in reduction of epithelial downgrowth (72%) and alveolar bone loss (36%) as compared to untreated-control groups. In the study of Huck et al. [358], kava-241 was found to be effective against *Porphyromonas gingivalis*-induced joint inflammation in a murine arthritis model. In the mentioned study, animals were treated with 50 μg/100 µL of *P. gingivalis*-LPS and 600 µL of kava-241 (40 mg/kg) for 17 days. Kava-241 treatment reduced the size of inflammatory cells and osteoctlasts in the side of inflammation. Moreover, Kava-241 inhibited the TNF-α production and TLRs protein expressions through the suppression of activation of ERK, MAPK, AKT and p38 proteins. Collectively, these studies support claims that *Piper* plants are potential candidates for treatment of inflammation-based diseases.

### 7.3. Neuropharmacological Activities

Uncontrolled systemic inflammation may proceed into a persistent chronic state that may have neurological impacts in the development of many neurodegenerative disorders including PD and AD [359]. Recent studies showed that therapeutic compounds acting on single targets (e.g., acetylcholinestaerase inhibitors) often show insufficient efficacy and undesirable toxic effects in the treatment of neuroinflammatory CNS disorders [360]. Studies suggest that balanced modulation of several interconnected targets can be more efficient strategy than the single target modulation for treating complex neuroinflammatory disorders with multi-factorial nature [360,361,362]. Plant species often serve good sources for the identification these kinds of multitarget agents. Extracts of *Piper* plants and their active compounds especially piperine, piperlongumine and kavalactones have been extensively investigated for the prevention and the treatment of neurodegenerative diseases through in vitro [363,364] and in vivo [365,366] studies as well as well-designed clinical trials [367,368]. In the last five years, the in vitro and in vivo studies for the effect of extracts on neurodegeneration models included *P. nigrum*, *P. betle* and *P. sarmentosum* species. Evidences from in vitro experiments support promising effects of last two species against the manifestations of neurodegenerative diseases.

*P. betle* is an abundantly found *Piper* species in certain regions of the world, particularly tropical areas. Ferreres et al. [363], investigated the phenolic profiles and anti-cholinesterase activity of both aqueous and ethanol extracts of leaves. Regarding chemical composition, hydroxychavicol was identified as a major phytochemical in both extracts. Both extracts demonstrated potent inhibitory efficacy against acetyl- and butyrylcholinesterase enzymes. In this study, the effects of extracts on the viability of neuronal cells (SH-SY5Y) were also evaluated at the mitochondrial function (MTT reduction) and membrane integrity (LDH release) levels. Accordingly, ethanol extract between 7.8–1000 μg/mL concentrations did not result in an alteration in the membrane integrity or the function of mitochondria. However, the aqueous extract at 125 µg/mL reduced the cell viability about 20% and interfered with the mitochondrial function. For this extract, more significant reductions in cell viability were reported above 500 μg/mL concentrations which were shown to be cytotoxic to neuroblastoma cells (human). The results of this study suggest that *P. betle* leaf extracts could be promising for the prevention/treatment of neurodegenerative disease.

*P. sarmentosum* (PS) is one of the edible, terrestrial species of *Piper* plants, abundantly found around the Asian regions. Traditionally, it has been extensively used for the treatment of various CNS disorders such as anxiety, depression and memory dysfunctions. Previous studies reported that *P. sarmentosum* has anti-depressant, anti-inflammatory, anti-oxidant and anti-acetylcholinesterase activities [364,369,370,371,372]. In the recent study of Yeo et al. [364], in vitro cytoprotective properties of different extracts from leaves of *P. sarmentosum* against Aβ-induced microglia-mediated neurotoxicity were investigated. Inhibitory effects of four extracts ethyl acetate (LEA), hexane (LHXN), dichloromethane (LDCM) and methanol (LMEOH) on Aβ-induced production and mRNA expression of some pro-inflammatory factors in BV-2 microglial cells were assessed. Additionally, the protective effects of extracts on human neuroblastoma cells (SH-SY5Y) were also evaluated by using Aβ-induced conditioned media from microglia cells. The LEA and LMEOH extracts resulted in reduction in the secretion levels of Aβ-induced pro-inflammatory cytokines (IL-1β and TNF-α) by downregulating the mRNA expressions of pro-inflammatory cytokines in BV-2 cells. LEA and LMEOH pre-treated conditioned media from microglia cells elicited protection on human neuroblastoma cell line against Aβ-induced neurotoxicity through downregulation of phosphorylated tau proteins. The results of this in vitro study suggest that polar extracts of *P. sarmentosum* leaves could be a promising complementary alternative in the treatment of AD.

Functional properties of *P. sarmentosum* as antidepressant were exhibited in rodent animal model by means of hypothalamic-pituitary-adrenal axis regulation [373]. In the study of Li et al. [373] these properties of the *P. sarmentosum* extract and its ethyl acetate fraction (PSY) were investigated by using several parameters including forced swimming test, open field test, and tail suspension test in mice. The results showed that treatment of mice with either *P. sarmentosum* extracts at 100 and 200 mg/kg or PSY at 12.5–50 mg/kg doses resulted in potent antidepressant effects, which is similar to response obtained by 20 mg/kg conventional therapeutic drug fluoxetine. Moreover, PSY increased the level of BDNF protein as well as phosphorylation levels of CREB and ERK proteins in the hippocampus of rats suggesting that *P. sarmentosum* can modulate the physiology of brain cells.

*P. nigrum* (black pepper) is among the most extensively studied species for its neuropharmacological activities. Anxiolytic, antidepressant, neuroprotective and antineuro-inflammatory effects of *P. nigrum* extracts have been examined in multiple animal studies. The methanolic extract prepared from the seeds of *P. nigrum* was investigated in management of AD stimulated by neuroinflammation in the rat model [374]. In this study, aluminum chloride (AlCl_3_) at 17 mg/kg b.w was used orally for one month to induce an AD model. Thereafter, animals in the group of AD were divided randomly into subgroups such as AD control; positive control (orally administered with a conventional drug-rivastigmine) and extract group (orally administerd with *P. nigrum* extract). Postmortem brains of the animal were examined for several parameters such as levels of monocyte chemoattractant protein-1 (MCP-1), acetylcholine, C-reactive protein (CRP) and NF-κB. Administration of AD rats with *P. nigrum* extract resulted in substantial improvements in the above-mentioned parameters which suggest that it may have potent anti-neuroinflammatory effects and may be promising in the treatment of AD.

In another study, the methanolic extract of *P. nigrum* was analyzed for its memory-enhancing and antioxidant properties in AD models of rats which were experimentally induced by amyloid beta (1–42) [375]. Rats were administered with extract at 50 and 100 mg/kg doses orally for 21 days. The memory-enhancing effects of the plant extract were studied by Y-maze and radial arm-maze tasks approaches. The antioxidant activities of the extract in the hippocampus were assessed by using superoxide dismutase-, catalase-, glutathione peroxidase-specific enzymatic assays and the total content of reduced glutathione (GSH), malondialdehyde, and protein carbonyl levels. Significant reduction in spontaneous alternations percentage within Y-maze task and increase of working memory and reference memory errors within radial arm-maze task were reported for AD group. However, treatment with the plant extract significantly ameliorated memory performance and showed remarkable antioxidant prospect.

Hritcu et al. [365], also elucidated anxiolytic and antidepressant properties of the methanolic extract of *P. nigrum* in a β-amyloid (1-42) rat model of AD. The mentioned effects of the extract were evaluated by numbers of open-arm entries, forced swimming tests and the time spent in open arms. In the Aβ (1-42)-treated AD models of rats, the number of open-arm entries together with percentage of the time spent in the open arms were significantly decreased which indicated that the Aβ (1-42)-treated rats experienced high levels of anxiety and they represented an efficient model to show the anxiolytic effects of extract. Accordingly, the administration of the methanolic extract increased the time spent in the open arms of Aβ (1-42)-treated rats significantly. The number of open-arm entries for Aβ (1-42)-and methanolic extract-treated rats increased as compared to the Aβ (1-42) treated group. The forced swimming test is one of the validated tools for predicting the antidepressant properties of drugs [376]. Regarding this, the swimming time significantly increased in the Aβ (1-42)- and methanolic extract-treated group as compared to the Aβ (1-42)-treated group. Taken together, results demonstrated that treatment with the methanolic extract elicited a marked anxiolytic and antidepressant effects.

There are important specialized phytoactives found in *Piper* species such as flavanones, chalcones, dihydrochalcones, and alkaloids as mentioned before. Piperine (1-piperoylpiperidine), a nitrogenous pungent alkaloid, is one of the major functionally active constituents responsible from neuropharmacological activities of *P. nigrum.* The neuroprotective, anti-neuroinflammatory and anti-depressant effects of piperine have been elucidated in multiple animal studies. In 6-hydroxydopamie (6-OHDA)-induced PD model of Wistar rats, Shrivastava et al. [366], reported that piperine treatment reduced neuronal cell apoptosis at a remarkable rate through inhibition of poly(ADP-ribose) polymerase activation and pro-apoptotic Bax levels as well as through the elevation of Bcl-2 levels. Furthermore, it was shown that piperine treatment reduced cytochrome-c release from mitochondria and diminished caspase-3 and caspase-9 activation induced by 6-OHDA. Treatment with piperine also caused a marked reduction of 6-OHDA-induced lipid peroxidation and stimulation of GSH levels in striatum brain region of rats. In this study, piperine also reduced the level of pro-inflammatory cytokines namely, TNF-α and IL-1β, in 6-OHDA-induced PD model of rats. Therefore, this study suggests that piperine has highly potent neuroprotective effect through its strong antioxidant and anti-inflammatory properties as wells as anti-apoptotic mechanism of action in 6-OHDA induced PD models.

Previous studies showed that piperine produces antidepressant-like effects through the inhibition of enzymatic activity of monoamine oxidase and increasing the levels of monoamine neurotransmitters in various mouse models of behavioral despair [324,377]. Two studies from the same research group also reported that intraperitoneal administration of piperine to mice caused a significant reduction in immobility time, an important parameter of serotonergic system, assessed by forced swim and tail suspension tests [378,379]. To clarify the molecular mechanism(s) underlying the antidepressant-like action of piperine Mao et al. [380] examined the effect of piperine treatment on depressive-like behavior and brain-derived neurotrophic factor (BDNF) protein expression in the hippocampus and frontal cortex of mice exposed to chronic unpredictable mild stress (CUMS). 10 mg/kg chronic treatment of piperine significantly attenuated behavioral deficits in CUMS mice evaluated by forced swimming and sucrose preference tests. In mechanism-based studies, piperine treatment significantly increased the expression level of BDNF protein in the hippocampus and frontal cortex of both naïve and CUMS mice. The antidepressant-like effects of piperine in CUMS mice were significantly blocked by the injection of K252a, an inhibitor of BDNF receptor (TrkB), suggesting that ameliorative potential of piperine are mostly related with its capacity to increase the BDNF level. In a different study Mao et al. [380], it was also reported that corticosterone-induced depression like behavior of model mice can be successfully suppressed by pretreating animals with piperine (at 5 and 10 mg/kg) for 21 days as assessed by diminished sucrose consumption which was found to be related with the elevated expression level of BDNF protein in the hippocampus. Regarding all these findings, it may be inferred that piperine exerts its antidepressant-like effect through modulating BDNF signaling.

Piperine treatment at 10 mg/kg also exerted a neuroprotective effects against a 1-methyl-4-phenyl-1,2,3,6-tetrahydropyridine) (MPTP)-induced mouse model of PD [381]. Accordingly, piperine treatment ameliorated the MPTP-induced disruptive effects in motor coordination and in cognition-related functions. It also reversed MPTP-induced decreases in the number of tyrosine hydroxylase-positive cells in the brain region of substantia nigra. In addition, piperine diminished the number of activated microglia and reduced the IL-1β expression and oxidative stress. Piperine also demonstrated an anti-apoptotic mechanism of action by restoring the imbalance between Bcl-2 and Bax proteins. The results of this study may suggest that piperine can be a promising therapeutic treatment alternative for PD due to its potent neuroprotective activities on dopaminergic neurons by means of anti-apoptotic and anti-inflammatory mechanisms of action.

An imbalance between autophagy and apoptosis has been detected in PD patients. While mitochondrial dependent apoptosis increases [382,383], the rate of autophagy decreases in the samples obtained from their brain [384,385]. Agents to restore this imbalance of apoptosis and autophagy have been under investigation as treatment strategy for PD. Liu et al. [386] reported that piperine elicits a cytoprotective activity in rotenone-induced SK-N-SH cells, primary rat cortical neurons and in mouse models by inducing phosphatase 2A (PP2A) and restoring the imbalance between autophagy and apoptosis. In SK-N-SH cells and primary neurons, piperine caused elevated cell viability and maintaining of mitochondrial functioning. Four week piperine administration (at two different doses; 25 and 50 mg/kg) ameliorated rotenone-induced disruption of motor functions and saved the loss of dopaminergic neurons in the substantia nigra region of PD mice models. In addition, it was reported that the rate of autophagy elevated by suppression of mammalian target of rapamycin complex 1(mTORC1) and activation of PP2A. A similar mechanism of action for piperine was also demonstrated in rat models. Wang et al. [387] showed that apoptosis was diminished in the presence of piperine, however, autophagy was stimulated therefore abolishing neuronal injury in the rotenone-induced rat model of PD. Two important results represented by both rat and mice models of PD in above-mentioned studies suggest a novel understanding for the neuroprotective action mechanism of piperine in the treatment or prevention of neurodegenerative disorders.

Piperine also exhibited strong anti-neuroinflamatory effect on LPS induced BV2 microglia cell line [388]. In this study, it was showed that piperine significantly inhibited LPS-induced TNF-α, IL-6, IL-1β, and PGE 2 production in BV2 cells through inhibition of NF-κB and activation of nuclear factor erythroid 2-related factor 2 (Nrf2). Recently, it has been suggested that chronic neuro-inflammation may lead to pathological amyloid β (Aβ) and τ accumulations in late-onset AD [389] which can be modelled in animals by intracerebroventricul-streptozotocin (ICV-STZ) treatment [390]. Recently it has been shown that piperine has relatively selective effects on cognition in the ICV-STZ animal model and it causes improved hippocampal function prior to significant Aβ deposition [390]. Accordingly, it was reported that the cognitive-enhancing effect induced by piperine at a relevant dose was simultaneous with hippocampal malonaldehyde decrement and the redox balance. In addition, histopathological outcomes were in accordance with the neuroprotective properties of piperine.

Taking all the abovementioned studies together, it can be concluded that piperine possesses profound effects on neurodegenerative disorders of the CNS such as AD and PD. However, the oral delivery of piperine to the brain is hampered due to many pharmaceutical challenges such as low water solubility, extensive first-pass metabolism and low absolute oral bioavailability [391]. Recently, the use of brain-targeted nanosystems has been extensively investigated to improve delivery characteristics of challenging lipophilic active compounds. Studies have shown that piperine encapsulation can be achieved using different solid lipid nanoparticles and results have indicated that piperine-loaded nanoparticles remarkably reduce the severity of neurodegenerative diseases modelled by experimental animals [391,392]. In a recent study by Etman et al. [393], piperine-loaded oral microemulsion as a nanosystem increased piperine efficacy and enhanced its delivery to the brain resulting in better therapeutic outcome compared to the free drug in male Wistar rats exposed to ICV-colchicine injection to induce sporadic dementia of Alzheimer’s type. In another recent study by Anissian et al. [394], piperine-loaded chitosan-sodium tripolyphosphate nanoparticles enhance the neuroprotection and ameliorate the astrocytes activation in chemical kindling model of epilepsy.

Piperlongumine (PL), an alkaloid amide, is the major active constituents of long pepper (*P. longum*). Piperlongumine has previously shown to have anti-inflammatory and anticancer activities [395,396]. Recent studies also showed that piperlongumine possess highly potent anti-neuroinflammatory functions. In vitro studies showed that piperlongumine significantly attenuated the production of proinflammatory mediators (NO and PGE_2_) and some cytokines (TNF-α and IL-6) by suppressing NF-κB signaling pathway in LPS induced BV-2 microglias, primary astrocytes, RAW264.7 macrophages and Jurkat cells [13,397,398]. Some important insights into the anti-neuroinflammatory and neuroprotective effects of piperlongumine have come from recent studies using animal models. Accordingly, piperlongumine inhibited LPS-induced memory impairment, Aβ accumulation and the activities of β- and γ-secretases in murine models [397]. Gu et al. [398], reported that the paralytic severity and neuropathology in mice model of experimental autoimmune encephalomyelitis (EAE) induced by myelin oligodendrocyte glycoprotein 35–55 immunization were reduced in piperlongumine-treated group in comparison with the EAE model group.

There are also some in vitro and animal studies showing neuroprotective effects of piperlongumine and its analogs in case of neurodegeneration. In the study of Peng et al. [399], two synthetic analogs of piperlongumine elicited low cytotoxicity and potent protection in hydrogen peroxide- and 6-hydroxydopamine-stimulated cell damage in the neuron-like PC12 cells via increasing the cellular levels of some antioxidant molecules. In rotenone-induced PD mouse models, Liu et al. [390] showed that four week piperlongumine (2 and 4 mg/kg) administration attenuated motor deficits and prevented the loss of dopaminergic neurons in the substantia nigra. In the same study, it was also indicated that piperlongumine improved cell viability and enhanced mitochondrial function in primary neurons and SK-N-SH cells through inhibition of apoptosis and induction of autophagy. Therefore, we can note that similar to piperine, piperlongumine also exerts its neuroprotective effects on PD by setting the disrupted balance between two key parameters namely, apoptosis and autophagy.

There is also some evidence to suggest that piperlongumine can be an efficient strategy to improve cognitive functions. In recent studies, piperlongumine has been reported to increase cognitive function in a transgenic mouse model of AD as well as hippocampal activities and cognitive dysfunction of aged mice [400,401]. In the hippocampus region of the aged mice, piperlongumine resulted in substantial elevation in the levels of calmodulin-dependent protein kinase II alpha (caMKIIα) and ERK1/2. Furthermore, following piperlongumine treatment in the dentate gyrus of the hippocampus the level of neurogenesis was significantly potentiated which was assessed by counting doublecortin-positive cells [401]. In regard of these results, piperlongumine can be suggested as a promising bioactive compound in treatment and prevention of age-related cognitive impairment and hippocampal changes.

Long standing neuropharmacological activities of *P. methysticum* including anxiolytic, sedative, muscle relaxant, mild anaesthetic and analgesic effects can be mostly attributable to kavalactones, particularly yangonin, kawain, dihydrokawain and methysticin, found in the lipid soluble fractions of the extracts [402]. Several in vitro and in vivo studies have elucidated possible biological action mechanisms of kavalactones including blockade of voltage-gated sodium and calcium ion channels, enhanced ligand binding to γ-aminobutyric acid A (GABA_A_) receptors, reduced neuronal reuptake of noradrenaline (norepinephrine) and dopamine, as well as weak inhibitory action on monoamine oxidase-B. Recently, Fragoulis et al. [403] reported that methysticin administration activates the Nrf2 pathway and reduces neuroinflammation, hippocampal oxidative damage and memory loss in transgenic mouse model of AD.

The medicinal use of kava extracts obtained from the root and rhizome parts as anxiolytic preparation has extended around the world since 1990 [404]. However, due to issues related with hepatotoxicity, the use of kava was forbidden by the Federal Institute of Drugs and Medical Devices of Germany in 2002. In the same year, Food and Drug Administration of USA recommended a consumer warning but never banned the use of kava [405]. Currently, in the USA kava extracts can be found in markets and on the internet. However in Germany, although the decision of court about the banning of kava use was found an inappropriate action in 2014, currently, it can be used only under the order of a referring practitioner [404,406].

### 7.4. Clinical Studies

Clinical studies on *Piper* plants have been largely focused on the use of kava extracts for treating anxiety disorders due to its widespread and as well as restricted uses around different regions of the world. Kava extracts prepared from the root and rhizome part of the plant have strong clinical records which substantiate its efficacy as anxiolytic agent.

The earliest randomized, placebo-controlled, clinical trial was performed on 58 anxiety patients with non-psychotic origin [367]. 100 mg kava extract (WS1490, a pharmaceutical extract) or placebo preparation was administered to patients daily three times during four week period. Drug receiving group demonstrated a significant reduction in overall score of anxiety symptomatology assessed by Hamilton-Anxiety-Scale (HAMA) as main target variable just after one week of treatment. The difference between drug and placebo group increased in the course of study. No adverse experiences were observed during the treatment period of kava extract.

A randomized, multicenter, placebo-controlled, double-blind clinical trial was carried out over a period of 25 weeks with 101 anxiety patients/non-psychotic origin [368]. All patients administered with either 110 mg kava extract (WS1490) or placebo three times a day. Drug receiving group showed a significant superiority in overall score of anxiety symptomatology assessed by Hamilton-Anxiety-Scale over placebo starting from week 8. Evenly distributed adverse events were observed rarely in both groups.

Another randomized, double-blind, placebo-controlled clinical trial was conducted on thirteen patients with generalized anxiety disorder (GAD) [407]. Patients were administered with kava 280 mg/day (standardized to 30% kavalactones) or placebo for 4 weeks. In this study two indication of vagal control which were defined as baroreflex control of heart rate (BRC) and respiratory sinus arrhythmia (RSA) were assessed. Accordingly, more patients showed significantly improved BRC following treatment with kava than with placebo. The magnitude of improvement in BRC was found to be significantly correlated with the degree of clinical improvement. In contrast, treatment with kava did not alter the magnitude of RSA, a measure of the heart rate changes occurring with respiration.

Mood disturbances, particularly anxiety and depression, are frequent in the premenopausal period [408,409]. Although, they spontaneously vanish with time, their manifestation may have great impact on the quality of life in women [410,411]. Hormone replacement therapy benzodiazepines, and antidepressants may ameliorate mood, but these pharmacological approaches sometimes may be associated with side effects and frequently be non-accepted by the women [409,412]. Kava extract has been proposed to exert positive effects on mood, particularly, anxiety of premenopausal women. In a 3-months randomized, prospective open study, the effects of Kava extract at two different doses (100 mg and 200 mg/day) were investigated in 80 perimenopausal women [413]. Several parameters such as anxiety assessed by state trait anxiety inventory scale, depression evaluated by Zung’s scale and climacteric symptoms shown by Greene’s scale were measured after first and third months. A placebo group was not included to the study and to compare the effects of Kava extract, a control group was used. As a result, in kava-treated groups, while anxiety and climacteric score declined at first and third months, depression was reduced at the third month. However, in the control group anxiety, depression and climacteric symptoms also showed decreasing tendency, so these findings were not significant.

In a double-blind and placebo-controlled trial, 50 anxiety patients with non-psychotic origin were treated by kava extract (WS 1490) 150 mg/daily for 4-week of period followed by 2-week observation phase to draw significant information on dosage range, safety, and efficacy of the preparation. For the primary efficacy variable assessed by HAMA score, kava treatment resulted in a significant reduction in anxiety as compared to placebo. For the secondary variables evaluated by HAMA subscales ‘somatic anxiety’ and ‘psychic anxiety’, a statistically significant advantage of the kava treatment over placebo was found to be detectable [414]. At the beginning and end of the trial, the laboratory tests demonstrated no pathological changes in the generalized biochemical parameters such as blood counts, hepatic enzyme levels, total bilirubin, glucose, and some lipid profiles. Therefore, it was concluded that the special kava extract can be classified as a well-tolerated and safe preparation without any drug-induced adverse reactions or symptoms associated with withdrawal.

In another double-blind clinical trial which was also carried out to investigate safety and efficacy profile of the same pharmaceutical kava extract (WS1490), 61 patients with sleep disturbances associated with anxiety, tension and restlessness states of non-psychotic origin were treated with daily doses of 200 mg extract or placebo over a period of 4 weeks [415]. ‘Quality of sleep’ and ‘Recuperative effect after sleep’, assessed by sleep questionnaire SF-B, demonstrated statistically significant group differences in favor of kava extract. Efficacy of kava was also indicated in the treatment of anxiety evaluated by HAMA scores. More prominent effects in terms of well-being based on self-rating and of global clinical assessment were also shown for kava extract. In clinical and laboratory parameters no differences were observed among groups. Besides, no drug-related adverse events or changes were seen. Safety and tolerability of kava were reported as good. Therefore, beyond the general anxiolytic effect of kava extract demonstrated in various controlled clinical trials this study evidenced that sleeping disorders related to non-psychotic anxiety may be treated by kava extract in an effective and safe ways.

An additional study to establish efficacy and safety of an aqueous extract of kava, a 3-week placebo-controlled double-blind, cross-over trial, specifically named as The Kava Anxiety Depression Spectrum Study (KADSS), was handled [416]. Sixty adult patients with 1 month or more of elevated GAD were prescribed daily five kava tablets (each containing 3.2 g, standardized to 50 mg of kavalactones) or placebo. Kava conferred a decrease of 11.4 points as compared to placebo on HAMA score. In addition, significant decrease in Beck Anxiety Inventory and Montgomery—Asberg Depression Rating Scale scores showed that it may also have antidepressant effects. In terms of safety concerns, extract did not resulted in serious adverse reactions.

According to a WHO commission report (Organization 2007) evaluating the safety of kava products special water-based preparation should be preferred over extracts obtained by organic solvents. In addition, more studies are needed for the developments of standardized aqueous extracts which should be validated by well-designed controlled clinical trials.

In a placebo-controlled, double blind, randomized clinical study the aqueous extract of kava was evaluated for the efficacy outcomes on anxiety, as well as was assessed on a range of secondary outcomes including liver function tests, withdrawal or addiction and female’s sexual drive [417]. Seventy five patients with GAD were administered with one tablet of kava twice a day (delivering 120 mg of kavalactones per day which could be increased to two tablets twice per day in non-response group-delivering 240 mg of kavalactones) for 6-weeks. The study contained a matched placebo group. Kava treatment resulted in significant reduction of anxiety as measured by HAMA score compared to placebo group [417,418]. No significant adverse reactions were found in kava group. In terms of liver function tests, no significant differences were detected across groups. Besides, no variations were reported between groups in terms of withdrawal or addiction behavior. According to Arizona Sexual Experience Scale (ASEX), kava resulted in significant increase in female sexual drive as compared to placebo. In males, negative effects were not reported. Interestingly, it was found that there was a highly significant correlation between improved sexual function and performance (decrease in ASEX score) and anxiety reduction in the whole sample. As a part of this trial, they also examined GABA transporter polymorphisms as potential pharmacogenetic markers of kava response and found that specific polymorphisms appear to potentially modify anxiolytic response to kava [418].

The same research group in 2015 extended the scope of the clinical trial and the number of participants [419]. The secondary outcomes enriched by several parameters including genomic and neuroimaging techniques. A bi-center, 18 weeks, randomized, double blind, placebo-controlled phase III study were designed. 210 currently anxious participants with diagnosed GAD who were non-medicated were administered with aqueous extract of kava (standardized to deliver 240 mg of kavalactones per day) or placebo. The trial has been completed by 2018. However, the results of this study have not been released yet.

Collectively, several recent well-designed clinical trials confirmed the potency and safety of kava extracts as anxiolytic preparations. However, although kava extracts can be a valuable and rational therapeutic options for patients suffering from anxiety and related disorders, physicians must be aware of the range of issues including different quality of kava extracts, patient’s liver function and simultaneous use of other medications before prescribing [418].

## 8. Conclusions and Future Perspectives

Pepper is called the “King of Spices” in the food industry, where it is used to enhance the flavor and texture of foods. Its characteristic aroma depends on its chemical composition. Some *Piper* species have a simple profile, while others, such as *P. nigrum*, *P. betle* and *P. auritum*, contain very diverse suites of secondary metabolites. The phytochemicals present in *Piper* species are responsible for their use in traditional medicine to treat several diseases worldwide. In this work, we summarize the usage of 106 *Piper* species that possess medicinal values and are used in traditional medicine in various parts of the tropical and subtropical regions.

In a world where food safety is a priority, several synthetic food additives such as BHA, BHT and PG have been investigated regarding their safety and possible adverse effects. This review also showed that *Piper* spp. could be used as food preservatives due to their antioxidant and antimicrobial potentials. In this review we have summarized the antioxidant potential of *Piper* species as antioxidants, e.g., the antioxidant activities of *P. nigrum* EO and oleoresins showed strong antioxidant activity in comparison with synthetic antioxidants [68]. Indeed, *P. nigrum* has demonstrated antibacterial and antifungal activities against human pathogens such as *C. albicans*, *E. coli*, *Aspergillus* spp., *Bacillus* spp., *Pseudomonas* spp., *Staphylococcus* spp., and *Salmonella* spp. Moreover, several *Piper* species, in particular *P. aduncum*, *P. betle* and *P. longum*, are used to treat parasitic diseases in Africa, Asia and Latin America. There is an increasing demand for natural food preservatives due to the emergence of antibiotic-resistance microorganisms. Particularly, *P. nigrum* is the most important species of this genus due to its pungent principle component, piperine, and its worldwide popularity as a flavoring for food. Spices and their EOs are considered to be GRAS which makes that *Piper* species have a promising future prospective as a food preservative to control various food spoilage and pathogenic microorganisms. However, *Piper* extract could affect food organoleptic characteristics. Therefore, careful selection of appropriate concentrations of this extract with regard to the sensory and compositional status of the food system to which it is applied is required in order to gain consumers’ acceptability. Consequently, new studies and technologies are needed to be developed to enhance food safety and quality without changing the organoleptic properties of the food itself.

Beyond the previously exposed uses of *Piper* species in traditional medicine, *Piper* species have shown their biological effects in different in vitro and in vivo studies and in clinical studies. Chronic illnesses and neurodegenerative disorders are the primary causes of disability and death all over the world. *Piper* species have demonstrated to possess therapeutic and preventive potential against several chronic disorders due their antiproliferative, anti-inflammatory, and neuropharmacological activities. Collectively, the studies developed with *Piper* species claim that these plants are potential candidate for treatment of inflammation-based diseases. Therefore, further efforts should be made to investigate standardized *Piper* plants using well-designed studies owing their widespread use. In addition, a wide range of possibilities are open for the development of functional foods based on *Piper* species.

## Figures and Tables

**Table 1:** Chemical compositions (%) of Brazilian *Piper aduncum* leaf essential oils.

Compound	Brazil [44]	Brazil	Brazil	Brazil	Brazil	Brazil	Brazil	Brazil	Brazil	Brazil	Brazil	Brazil	Brazil	Brazil	Brazil	Brazil	Brazil	Brazil	Brazil	Brazil	Brazil [45]	Brazil	Brazil	Brazil [46]	Brazil [42]	Brazil [47]
α-Humulene						5.5										8.5–10.6										
(*E*)-Nerolidol		80.6–82.5	79.2–81.2			10.3							5.9			14.3–16.7					25.2					
(*E*)-β-Ocimene				6.4		5.0			11.6				13.4							13.9					4.1	
(*Z*)-cadin-4-en-7-ol																7.5–12.2										
(*Z*)-β-Ocimene																				7.0						
1,8-Cineole				42.0–42.5								57.2		8.7	55.8											
Asaricin				9.2–10.5			5.6		15.8								14.9	80.1	73.4							
Bicyclogermacrene				3.8–6.0		11.3														20.9						
Camphene														10.9												
Camphor														17.1												
Dillapiole	76				94.8					49.5	79.0													92	6.3	52.4
Germacrene D																									6.9	
Limonene																										
Linalool						31.8										9.3–13.4					13.4					
Longipinanol		2.4–5.6	11.1–13.6																							
Myristicin																									12.4	
Piperitone							22.7	24.9	11.0	16.3				34.0											11.8	
Safrole																	13.3	10.8	10.5	6.2						
Spathulenol																0–5.6	10.6			5.3	6.3					
Terpinen-4-ol							15.0	16.8	6.7																6.3	
Valencene													6.9				9.7									
Viridiflorol														7.4											4.4	
α-Pinene				8.0–8.9								14.2					6.4									
α-Selinene																										4.7
α-Terpinene																										4.8
α-Terpineol															5.9											
β-Caryophyllene	6					9.3										5.1–6.7									5.4	5.8
β-Phellandrene							6.8	6.6																		
β-Pinene	3.5			6.6–7.0								9.0														
γ-Cadinene																				5.5						
γ-Terpinene							8.3	8.2																		9.0

Taken from [32] unless otherwise specified.

**Table 2:** Chemical compositions (%) of *Piper aduncum* leaf essential oils from countries other than Brazil.

Compound	Cuba	Cuba	Cuba	Costa Rica	Bolivia	Panama [48]	Equador [43]	Papua New Guinea [40]
α-Humulene								5.1
(*E*)-β-Ocimene							7.5	
1-*epi*-Cubenol		6.2						
1,8-Cineole					40.5			
Aromadendrene						13.4		
Asaricin					12.9			
Camphene		6.1	5.4–7.4					
Camphor		8.3	9.4–13.9					
Dillapiole	82.2						48.2	43.3
Germacrene D		8.2						
Limonene				6.7	5.0			
Linalool						8.6		
Piperitone		12.9	19.0–20.1					6.7
Sabinene				18.4				
Viridiflorol			13.0–18.8					
α-Pinene				39.3	9.0	8.8		
β-Caryophyllene		6.7				17.4	4.8	8.2
β-Pinene					7.1			

Taken from [32] unless otherwise specified.

**Table 3:** Chemical composition (%) of Brazilian *Piper aduncum* essential oils from aerial parts *.

Compound	%	%	%	%	%	%	% [49]	%	%	%	*
(*E*)-β-Ocimene							3.0				
Dillapiole	82.2	86.9	91.1	91.1	88.1	86.9	64.4	85.9	73.0	50.8	56.3
Piperitone							3.3			13.9	7.0
Terpinen-4-ol										7.3	
γ-Terpinene										6.5	
β-Caryophyllene							2.5				
Germacrene D							2.7				
Bicyclogermacrene							2.0				
Terpinolene							2.0				

Taken from [32] unless otherwise specified.

**Table 4:** Chemical compositions (%) of *Piper aduncum* essential oils from aerial parts, other countries.

Compound	Cuba [41]	Equador [36]	Bolivia [36]	China [34]
(*E*)-β-Ocimene		10.4		
1,8-Cineole			40	
Camphene	5.9			
Camphor	17.1			
Dillapiole		45.9		
Piperitone	23.7	8.5		
Terpinen-4-ol		3.1		
Viridiflorol	14.5			
γ-Terpinene		2.4		
β-Caryophyllene		2.6		
Eugenol				29.1
Spathulenol				8.3
Propiopiperone				7.1
Germacrene D				5.8
Bicyclogermacrene				3.9
Methyleugenol				3.8

**Table 5:** Chemical compositions (%) of *Piper aduncum* floral essential oils.

Compound	Brazil [32]	Brazil [42]
(*E*)-β-Ocimene	11.1	
Piperitone		23.4
Terpinen-4-ol		23.4
γ-Terpinene	12.0	
α-Terpinene	6.8	
(*Z*)-β-Ocimene	5.6	
Linalool	41.2	
(*E*)-Nerolidol	6.1	
β-Caryophyllene		7.2
α-Humulene		6.9
Myristicin		6.5

**Table 6:** Chemical compositions (%) of *Piper amalago* leaf essential oils.

Compound	Costa Rica	Brazil [50]	Brazil	Brazil	Brazil	Brazil	Brazil	Brazil	Brazil
Borneol									5.7
Bicyclogermacrene		13.0	19.4	20.8	15.0			27.9	
Camphene									8.9
Cubenol							6.2		
Germacrene A	6.5–9.7								
Germacrene D	28.9–29.4					11.7		9.9	
Limonene		6.2							6.8
Methyl geraniate							7.8		
Myrcene			6.8						
*p*-Cymene							9.4		
Sabinene			8.2	6.7					
Spathulenol			5.6	9.1				19.2	
α-Amorphene							25.7		
α-Cadinol								7.6	
α-Phellandrene	1.7–8.1								
α-Pinene		3.7		6.7	11.7	14.8			30.5
β-Caryophyllene	15.9–23.3	3.0							
β-Elemene	11.5–24.6								
β-Phellandrene			12.3	15.9	33.1	39.3			
γ-Muurolene		3.6	5.9					7.3	

Taken from [32] unless otherwise specified.

**Table 7:** Chemical compositions (%) of *Piper betle* leaf essential oils.

Compound	Malaysia	India	India ^a^	India ^a^	India [54]	India ^c^ [55]	India [56]	India [57]	India [58]	Thailand	Vietnam	Nepal [59]
(*E*)-Isoeugenol			5.2 ^b^							28.3	72.0 ^b^	
Chavicyl acetate										8.1		
Eugenyl acetate									31.4			
Allyl-pyrocatechol		8.7–10.8		2.1								
Allyl-pyrocatechyl diacetate				3.6								6.2
Allyl-pyrocatechyl monoacetate				8.5								
Aromadendrene		0.89–1.35										
Bicyclogermacrene										1.0		
Chavibetol	69.0	4.2–7.2		2.0	22.0		53.1		26.0			80.5
Chavibetyl acetate		14.7–20.7					15.5	12.5				11.7
Chavicol	6.0	47.8–48.8	1.1		11.8					2.0	3.2	0.4
Estragole					15.8							
Eugenol			63.6	9.0		63.4						0.4
Eugenyl acetate	8.3		18.7	2.2		14.1				31.8		
Germacrene D										2.9		
Isoeugenyl acetate											12.2 ^b^	
Ledene										1.0		
Linalool				1.8						0.9		
Methyl eugenol			6.9							0.7		0.4
*p*-Menth-3-en-9-ol										1.5		
Sabinene				2.6								
Safrole				39.9				48.7				
Terpinen-4-ol				6.3								
Viridiflorol										1.5		
α-Amorphene										2.5		
α-Humulene										1.0		tr
β-Bergamotene		0.7–1.3										
β-Caryophyllene	2.4			1.2	11.3	4.2				3.1		0.4
β-Cubebene					13.6							
β-Pinene				1.7								
γ-Cadinene	1.6			2.4								
γ-Muurolene	5.2											
γ-Terpinene				1.9								

Taken from [53] unless otherwise specified. ^a^ Sagar Bangla cultivar, ^b^ isomer not determined, ^c^ Magahi cultivar.

**Table 8:** Average compositions of *Piper cubeba* fruit essential oils.

Molecule	Percentage
(*E*)-Asarone	0.9–3.7
(*E*)-Nerolidol	0.1–3.6
(*E*)-α-Bergamotene	<0.1–0.2
(*E*)-β-Farnesene	<0.1–0.2
(*E*)-β-Ocimene	<0.1–0.1
*cis*-Calamenene	1.0–3.8
*cis*-Sabinene hydrate	<0.1–0.4
1-*epi*-Cubenol	0.3–3.5
1,8-Cineole	0.3–0.8
*allo*-Aromadendrene	0.2–11.0
Apiole	<0.1–0.2
Borneol	<0.1–0.3
Cadina-1, 4-diene	<0.1–0.2
Caryophyllene oxide	<0.1–0.1
Cubebol	5.6–30.9
Cuminaldehyde	<0.1–0.2
Cyclosativene	<0.1–0.2
Dillapiole	<0.1–0.2
*epi*-Cubebol	<0.1–4.6
Germacrene D	0.1–11.1
Globulol	<0.1–3.5
Ledol	<0.1–0.2
Limonene	0.1–4.4
Linalool	<0.1–1.0
Myrcene	<0.1–1.7
Myristicin	<0.1–0.1
*p*-Cymen-8-ol	0.1–0.3
*p*-Cymene	<0.1–1.1
Sabinene	0.7–29.6
Safrole	<0.1–0.1
τ-Muurolol	<0.1–0.3
Terpinen-4-ol	<0.1–2.7
Terpinolene	<0.1–0.3
α-Cadinol	0.2–1.0
α-Copaene	3.8–14.3
α-Cubebene	1.5–5.7
α-Humulene	0.5–0.9
α-Muurolene	0.6–1.7
α-Pinene	0.3–7.9
α-Terpinene	<0.1–1.3
α-Terpineol	0.1–2.8
α-Thujene	<0.1–2.5
β-Bisabolene	1.5–2.0
β-Caryophyllene	1.1–9.5
β-Cubebene	0.2–11.1
β-Elemene	1.0–9.4
β-Phellandrene	<0.1–0.8
β-Pinene	<0.1–0.2
γ-Cadinene	0.1–0.3
γ-Muurolene	<0.1–11.5
γ-Terpinene	0.1–0.7
δ-Cadinene	<0.1–9.5
δ-Elemene	0.1–0.3

**Table 9:** Chemical compositions (%) of *Piper cubeba* fruit essential oils.

Molecule	India	India	India	Indonesia	Brazil ^a^ [61]
*trans*-Muurola-4(14),5-diene			0.8		
(*E*)-Nerolidol			0.7		
*trans*-Sabinene hydrate		0.5			
(*Z*,*Z*)-Farnesol	0.6				
*cis*-Calamenene			0.7		
*cis*-Sabinene hydrate		0.9	0.7		
1-*epi*-Cubenol			0.4		
1,8-Cineole			1.3		11.9
4-*epi*-Cubenol			1.9		
Terpinen-4-ol		0.9	1.0		6.4
*allo*-Aromadendrene	2.3	3.1	4.1		
Bicyclogermacrene		1.5	1.5		
Camphor					5.6
Caryophyllene oxide	1.3		0.6		
Cedrol	0.3				
Citronellyl acetate	0.5				
Cubebol	23.6	4.7	13.3		
Germacrene D	1.5	2.6	4.7	7.5	
Guaiol	1.0				
Isoborneol					3.6
Limonene	2.0		0.5		
Linalool	1.5	4.9	3.2		5.0
Linalool oxide	1.4				
Methyl eugenol	2.3				
Myrcenol	0.6				
*p*-Cymene	0.4	1.0	0.4		4.4
Sabinene		19.4	9.6		20.0
Sativene				8.7	
Spathulenol			0.5	27.1	
Terpinolene	0.2				
α-Cadinol	0.9				
α-Copaene	0.9	8.8	7.4		4.9
α-Cubebene		4.1	3.9		
α-Gurjunene			0.7		
α-Humulene	0.3	0.9	2.0		
α-Muurolene		0.6	0.6		
α-Pinene	2.2	4.1	1.9		
α-Terpineol	1.7		0.3		4.1
α-Thujene		4.5	1.9		3.3
β-Bisabolene	3.1				
β-Bisabolol	1.0				
β-Caryophyllene	0.4	3.7	5.3		
β-Copaene	3.3				
β-Cubebene	5.6	18.3	18.9		
β-Elemene	7.3	0.6	1.4		
β-Eudesmol	2.4				
β-Phellandrene		5.9	1.7		
β-Pinene	18.2	0.7	0.6		5.8
β-Selinene			0.6		
γ-Amorphene		2.0			
γ-Cadinene		0.9			
γ-Muurolene			2.4		
γ-Terpinene	0.7		0.1		3.4
δ-3-Carene	0.3				5.3
δ-Cadinene	4.7		0.2		
δ-Elemene			0.1		
δ-Terpineol			0.6		

Taken from [60] unless otherwise specified. ^a^ The EO was purchased and not distilled in the lab.

**Table 10:** Major components (%) of *Piper nigrum* L. fruit essential oils [64,65].

Compounds	Black	White
(*E*)-β-Farnesene	tr.-3.3	
Limonene	16.4–24.4	22.6
*p*-Cymene		1.0
Sabinene	0.1–13.8	
α-Copaene	0.1–3.9	
α-Cubebene	0.2–1.6	
α-Humulene		1.0
α-Phellandrene		4.5
α-Pinene	1.1–16.2	4.0
β-Bisabolene	0.1–5.2	
β-Caryophyllene	9.4–30.9	23.4
β-Myrcene		2.7
β-Pinene	4.9–14.3	9.3
δ-3-Carene	tr.-15.5	25.2
δ-Elemene		2.1

**Table 11:** Chemical compositions (%) of *Piper nigrum* L. fruit essential oils [66].

Compound	Malaysia	Malaysia White, Green, Black	Indian Fresh and Dried Black	India Ground Black	Indian Cultivars	Cuba
(*E*)-Nerolidol			tr.-7.1		tr.-7.1	
6-Hydroxypiperitol						0.6
*ar*-Turmerone						0.7
Caryophyllene oxide	4.1	0.4–3.7		6.8–9.9		29.3
Caryophyllenol						0.5
Eugenol		1.1–41.0^a^				
Humulene epoxide II						1.4
Isocaryophyllene oxide						1.6
Isospathulenol						3.1
Limonene	8.7	2.9–14.3	18.8–20.1	22.7–26.2	8.3–23.8	3.7
Linalool						0.6
Myrcene	9.1					
Myrtenol						0.5
*p*-Cymen-8-ol						1.4
*p*-Cymene			tr.-13.0		11.0–13.2	0.8
Piperitenone oxide						0.0–2.0
Sabinene	2.4	0.8–12.1		16.7–24.5	0.0–27.5	
Terpinen-4-ol			tr.-8.9		tr.-8.9	
*trans*-Sabinol						0.5
Verbenone						2.0
α-Copaene	3.8			3.6–4.5		3.1
α-Humulene	2.8					0.8
α-Pinene	0.3	0.3–3.8	5.4–11.2	3.4–4.6	1.7–14.6	3.1
α-Selinene	1.1					
α-Terpinene						7.3
β-Bisabolene				4.4–7.2		
β-Caryophyllene	39.7	3.5–70.4	6.7–10.8	1.6–2.1	6.4–52.9	6.9
β-Elemene	1.6					
β-Eudesmol		tr.-9.7				
β-Pinene		0.7–5.9	14.2–15.2	10.5–14.4	0–23.9	
β-Selinene	1.1					
δ-Cadinene	3.9					
δ-3-Carene	10.9	1.7–13.8	0.2-8.0		0–23.4	
δ-Elemene	2.5					1.6

^a^ According to Lawrence [66] the high levels of eugenol found in oils produced from ground green and white pepper are extremely unusual [34,63,67,68,69,70,71].

**Table 12:** Chemical compositions (%) of *Piper nigrum* L. fruit essential oils from other sources.

Compound	West Africa [69]	India [68]	Malaysia [67]	China Black [63]	China White [63]	China Green [63]	China Black [34]	China Red [34]	China White [34]	Black Commercial [70]	Green Commercial [70]	Commercial [71]
β-Elemene								4.3				
Caryophylla-4(12),8(13)-dien-5β-ol							10.0					
Caryophyllene oxide		3.9		1.3–9.3	4.1–6.7	1.5–4.54	4.7–13.5				9.0	
β-Selinene							5.1–8.0					
γ-Selinene							6.2	5.6				
Isospathulenol							4.9		7.6			
Limonene	18.8		15.0	7.2–13.3	12.9–14.9	9.7–11.7					19.0	
Linalool										12.1		
*p*-Cymene					4.6–5.2							
Sabinene	16.5	5.9	13.8							7.5		
α-Copaene			3.7									4.2
α-Humulene						2.6–5.9	5.5	6.3				
α-Phellandrene				0.2–6.2								
α-Pinene		4.5	8.0	5.2	4.3–5.8	3.4–5.6					10.0	5.5
α-Selinene										5.3		
β-Bisabolene		3.9								4.7		
β-Caryophyllene	15.4	29.9	18.6	15.1–16.3	8.9–15.9	12.9–18.4	42.0–51.8	55.2	58.9	30.3		26.2
β-Pinene	15.4	7.9	9.7	6.6–8.9	9.3–11.0	7.7–8.2				7.4	24.0	
δ-3-Carene	4.4	8.6	12.3–19.4	16.0–22.1	14.5–18.9				5.3	19.7		
δ-Elemene				7.3–8.3	4.2–5.4	8.0–8.4						4.1

**Table 13:** Chemical compositions (%) of *Piper longum* L. fruit essential oils [73].

Compound	Sample 1 (1978)	Sample 2 (1997)	Sample 3 (Indonesian)	Sample 4 (2008 Bangladesh—Leaves and Inflorescence-Rich Spike)	Sample 5 (2011)
*trans*-Cadinα-1(6),4-diene					5.4
(*E*)-Cinnamyl acetate				5.9	
(*E*)-Nerolidol					1.3
(*E*)-β-Farnesene					6.8
(*Z*)-β-Farnesene		3.7			1.1
1-Pentadecene					7.1
1-Tridecene					1.5
1,8-Cineole	0.5				
14-Hydroxy-isocaryophyllene					1.9
7-*epi*-α-Selinene			3.0		
8-Heptadecene			7.4		
Apiole					3.9
*ar*-Curcumene			4.8		
Caryophyllene oxide	3.7		1.5		5.4
Cubebol	4.0				
Cubenol					1.4
Eugenol				33.1	
Germacrene B		1.8			
Germacrene D	10.3	4.9	16.5		
Globulol		2.6			1.5
Heptadecane	1.3	5.7	9.6		4.7
Heptadecene		2.3			
Muurola-4(10(14)-dien-1β-ol					1.7
Myristicin					3.0
Nonadecane isomers			5.9		
Pentadecane isomers	1.3	19.6	6.6		15.8
Selina-3,11-diene + *ar*-curcumene	13.8				
Selina-4,11-diene	1.5				
Spathulenol		3.0			6.6
Tridecane					4.7
Zingiberene		5.0			
α-Copaene		1.6			2.6
α-Humulene		2.0	2.9		
α-Terpineol + borneol	2.6				
β-Bisabolene		11.2	3.3		5.9
β-Caryophyllene		17.0	10.2	9.3	
β-Caryophyllene + terpinen-4-ol	10.0				
β-Elemene					2.4
β-Selinene	12.4		3.9		
δ-Cadinene	10.3				

**Table 14:** Chemical compositions (%) of *Piper arboreum* Aubl. leaf essential oils.

Compound	Panama [32,48]	Brazil [32]	Brazil [32]	Brazil [32]	Brazil var *latifolium* [32]	Brazil [74]
*trans*-Cadinα-1(6),4-diene				9.6		
(*E*)-Nerolidol	5.2					
(*E*)-β-Ocimene						1.4
(*Z*)-β-Ocimene						2.3
1-*epi*-Cubenol				10.4		
10-*epi*-γ-Eudesmol						4.4
Bicyclogermacrene		12.1	49.5			
Bulnesol						8.1
Caryophyllene oxide		10.2		5.9		
Germacrene A						3.3
Germacrene D	5.3		9.6		72.9	3.6
Octanal					5.5	
Sabinene	4.0					
Spathulenol		8.4		7.9		
α-Cadinol				5.4		
α-Humulene						1.2
α-Copaene	7.4			5.6		
α-Eudesmol						12.2
α-Muurolene	4.2					
α-Pinene	4.3					
β-Bisabolene				12.6		
β-Caryophyllene	4.4		25.1			
β-Pinene	6.6					
γ-Elemene					6.8	
γ-Eudesmol						14.6
δ-Cadinene	25.8					
δ-Elemene						3.1

**Table 15:** Chemical compositions (%) of *Piper cernuum* Vell. Leaf essential oils from Brazil [32].

Compounds	Sample 1	Sample 2	Sample 3	Sample 4	Sample 5	Sample 6	Sample 7	Sample 8	Sample 9 (var *cernuum*)
*trans*-Dihydroagarofuran					28.7	33.8		30.0–36.7	
*cis*-Dihydroagarofuran									32.3
(*Z*)-α-Bisabolene		5.7							
*cis*-β-Guaiene			8.2						
10-*epi*-γ-Eudesmol					13.5	12.2			
4-*epi*-*cis*-Dihydroagarofuran					10.8			11.2–13.4	
Bicyclogermacrene	21.9	25.1		19.9					
Camphene					6.3	8.7			5.3
Caryophyllene oxide			7.7				5.1		
Elemol								5.9–9.2	6.7
*epi*-Cubebol			13.1						
Germacrene D	6.7	9.3		12.7					
Spathulenol		7.2	9.6				11.5		
Valeranone			9.1						
α-Muurolol							5.8		
α-Pinene	7.2				10.0	11.8	11.4	2.6–5.4	10.2
β-Caryophyllene	20.7	22.2	8.3	16.3			6.9	5.9–8.7	
β-Elemene		7.2	11.6	30.0			10.1		
β-Pinene	6.2						7.9		7.4
γ-Eudesmol								8.3–13.3	
γ-Muurolene			7.6						
τ-Muurolol							6.2		

**Table 16:** Chemical compositions (%) of *Piper dilatatum* Rich. aerial parts essential oils from Brazil [32].

Compounds	1	2	3	4	5	6	7	8	9	10	11	12
(*E*)-Nerolidol			10.2		5.7							
(*Z*)-α-Bisabolene									39.3		23.2	
(*Z*)-β-Farnesene									7.0			
(*Z*)-β-Ocimene				10.0								
Atractylone					5.1							
Bicyclogermacrene			7.4	27.6		9.4	7.9		6.7	34.7		13.2
Caryophyllene oxide	6.1											
Curzerene					13.8						28.7	
Germacrene D	6.7	10.2	12.6			30.2	18.5		24.5	8.5	15.2	43.0
Hinesol			6.4			6.4						
Limonene								6.4				19.4
*p*-Cymene					11.7		5.1					
Spathulenol			11.8	15.0		40.6		9.3		35.2		
α-Cadinol	12.2	7.0	5.8			6.4						
α-Eudesmol					8.0							
α-Pinene				9.7								
α-Selinene	6.1	6.9										
β-Bisabolene									8.1		5.5	
β-Caryophyllene	11.7	15.5		7.4			5.1					
β-Elemene							21.8	13.8				
β-Pinene				14.8				10.5				
β-Selinene					6.4							
δ-Cadinene	5.4	8.5										
δ-Elemene								7.6				

**Table 17:** Chemical compositions (%) of *Piper gaudichaudianum* Kunth essential oils.

Compounds	Brazil	Brazil	Brazil	Brazil	Brazil	Brazil	Brazil	Brazil CT 1 [78]	Brazil CT 2 [78]	Brazil	Brazil	Brazil Inflorescence	Brazil Fol Fresh	Brazil Fructus	Brazil Leaves and Branches
(*E*)-Nerolidol	5.3–22.4		22.4		22.1				5.0						
*trans*-β-Guaiene						6.9									
1-*epi*-Cubenol								24.2							
*allo*-Aromadendrene	7.7														
Aromadendrene		15.6													
Bicyclogermacrene	7.4		7.4		13.2			5.1	6.4						
Cadalene								33.7							
Caryophyllene oxide						8.5									
Dillapiole												83.1–87.5	61.6–69.2	85.2–87.8	
Germacrene B													4.7–6.9		
Hinesol				6.4											
Ishwarane		10.0													
Limonene															0.5
Myristicin												6.2–9.3		6.9–11.9	
Selin-11-en-4α-ol		8.5													
Viridiflorene				8.1											
Viridiflorol		27.5													
α-Cadinol				7.0											
α-Humulene	13.3–37.5		16.5	23.4	21.3					13.3	29.2		8.2–13.3		13.3–29.2
α-Pinene						12.2			9.7						
α-Selinene	8.9–16.6									16.6	8.9				16.6–8.9
β-Caryophyllene	10.4–19.3		8.9	15.6	7.5	8.5			17.8	12.1	19.3				12.1–19.3
β-Pinene	5.6–7.0					7.0			13.8						
β-Selinene	3.7–15.7	10.5		6.6						15.7					15.7–3.7
γ-Elemene							5.4								
δ-3-Carene							5.9								
δ-Cadinene							45.3	6.9							

Taken from [77] unless otherwise specified.

**Table 18:** Chemical compositions (%) of *Piper hispidum* Sw. and *Piper hispidinervum* C.DC. essential oils.

Compounds	Brazil	Brazil	Brazil	Brazil	Brazil [44]	Brazil [44]	Brazil [47]	Brazil [78]	Brazil	Brazil [74]	Cuba	Colombia [79]	Venezuela	Panama [48]	Cuba [80]
(*E*,*E*)-α-Farnesene										5.6					
(*E*)-Nerolidol												23.6			
14-Hydroxy-α-muurolene											5.0				
9-*epi*-Caryophyllene										4.3					
Bicyclogermacrene								11.5							
Camphene	15.6														
Caryophyllene oxide			5.9							5.0		5.4	7.8		
Curzerene											12.9	4.9			
Dillapiole														57.7	
Elemol											7.6	3.6			
Germacrene B								6.1				4.5	5.2		
Germacrene D							9.7			7.1					
Guaiene										3.4					
Humulene epoxide II										3.6					
Khusimene									12.1						
Ledol									8.8						
Limonene			6.9												16.3
Linalool							32.5								9.6
Myristicin					2.0										
*p*-Cymene						12.1									
Piperitone														10.0	
Safrole					91.4										
Spathulenol		6.2								4.1			5.0		
Terpinolene						7.3									
Viridiflorol							6.3								
α-Cadinol		6.9													
α-Copaene			7.3	28.7–36.2											
α-Eudesmol											8.1				
α-Guaiene	11.5														
α-Humulene			9.5												
α-Pinene	5.2	9.0		7.1–13.9					6.6	1.4		3.4	15.3		
α-Selinene						9.0									
α-Terpinene						14.4									
α-Terpineol															8.5
β-Caryophyllene	5.4		10.5			5.3	22.0			6.3		5.1	6.2	4.3	
β-Elemene												5.1	8.1		
β-Eudesmol											17.5				
β-Phellandrene	9.7														
β-Pinene		19.7		7.5–13.3					12.0			3.6			14.5
β-Selinene			5.1			8.1							14.8		
γ-Cadinene	25.1								13.2	3.0					
γ-Elemene	10.9											2.8			
γ-Eudesmol											9.3				
γ-Muurolene												4.0			
γ-Terpinene						30.4									
δ-3-Carene		7.4	9.1										6.9		
δ-Cadinene									6.3	4.0					

Taken from [32] unless otherwise stated.

**Table 19:** Chemical compositions (%) of *Piper guineense* Schumach. & Thonn. fruit essential oils.

Compounds	Nigeria [84]	Nigeria [85]	Nigeria [83]	Cameroon [82]	S. Tomé e Príncipe [69]	Colombia [79]
(*E*)-Nerolidol						23.6
(*E*)-α-Bisabolene			4.3			
β-Bisabolene	5.4					
1,8-Cineole		17.2				
3,5-Dimethoxytoluene				10.9		
Bicyclogermacrene			6.0			
Camphene				4.8		
Camphor			3.2			
Caryophyllene oxide						5.4
Curzerene						4.9
Dillapiole					44.8	
Germacrene B			5.7			4.5
Ishwarane			1.4			
Limonene	5.8					
Linalool	6.1			41.8		
Myrcene		4.4				
Myristicin					9.8	
Sabinene			3.3			
α-Cubebene			3.4			
α-Phellandrene					8.2	
α-Pinene	5.3	13.6	5.8			
β-Caryophyllene			6.9			5.1
β-Elemene						5.1
β-Pinene		41.2	23.2	9.2		
β-Sesquiphellandrene	20.9					
γ-Terpinene		5.7				
δ-3-Carene			5.7			

**Table 20:** Chemical compositions (%) of *Piper marginatum* Jacq. leaf essential oils.

Compounds	Brazil [86]: 22 Samples	Brazil [32]: 24 Samples	Brazil [44]	Panama [48]	Colombia [75]
(*E*)-Anethole	0.1–26.4	13.6–26.4			
(*E*)-Asarone	0.2–10.8	6.4–10.8			
(*E*)-β-Ocimene	0.4–15.2	5.2–15.2	8.3		
(*Z*)-Anethole	3.0–8.4	6.0–8.4			
(*Z*)-Asarone	1.7–8.8	8.8–30.4			
(*Z*)-β-Ocimene	0.2–8.6	5.2–8.6	6.0		
2- Methoxy-4,5-(methylenedioxy)propiophenone	1.1–26.3	26.3			
3,4-Methylenedoxypropiophenone	7.3– 40.7				
Bicyclogermacrene	0.2–11.7	6.4–11.7			4.0
Crocatone		21.9			
Elemicin	0.1–6.5	6.5			
Elemol		9.7			
Exalataxin	7.9	7.9			
Germacrene D	0.5–10.4	5.5–10.4			
Isoosmorhizol	15.8–46.8	15.8–46.8			
Isosafrole				34.4	
Methoxy-4,5-(methylenedioxy)propiophenone isomer 5	2.0–21.9				
Methyl eugenol	0.1–5.9	5.9	5.4		
Myristicin	0.2–16.0	5.0–16.0			
Myristicin derivative				10.7	
Nothosmorhizol	2.9–24.5	11.2–24.5			
*p*-Menthα-1(7),8-diene	0.1–39.0	5.2–39.0			
Patchouli alcohol		16.0			
Propriopiperone		7.3–40.7	13.2		
Safrole	0.2–63.9	5.7–63.9	4.6		
Spathulenol	0.3–6.6	6.6			
α-Acoradiene		5.1			
α-Copaene	0.1–11.4				
α-Pinene	0.1–7.3	5.0			
β-Caryophyllene	1.2–13.6	6.0–13.6	6.3		11.0
γ-Elemene 8.5%		5.6–11.4			
γ-Terpinene	0.1–14.4	6.5–14.4		10.5	
δ-3-Carene			11.3		
τ-Muurolol		5.0			
α-Phellandrene					11.0
Limonene					8.0
β-Elemene					4.0

**Table 21:** Chemical compositions (%) of *Piper tuberculatum* Jacq. essential oils.

Compounds	Brazil (Fruit) [88]	Brazil (Fruit) [38]	Brazil (Stem) [32]	Brazil (Stem) [38]	Brazil (Leaf) [38]	Brazil (Leaf) [32]	Brazil (Leaf) [32]	Brazil (Leaf) [44]	Brazil (Leaf) [78]	Brazil (Leaf) ^a^ [32]	Brazil (Floral) [32]
(*E*)-Nerolidol							12.7	6.5			
(*E*)-β-Farnesene						8.3				6.1	
(*E*)-β-Ocimene	12.5		14.5			8.6				9.0	9.8
Caryophyllene oxide								13.3	6.4		
Germacrene D						5.5			8.4		
Limonene	3.0	2.4		2.1	4.2			1.6		6.7	
Spathulenol							15.8				
Viridiflorol							13.5				
α-Copaene								1.3	5.4		
α-Cadinol										13.7	
α-Pinene	26.5	28.7	17.3	17.3	10.4	10.4		9.4		8.4	28.7
β-Bisabolene								9.1			
β-Caryophyllene	14.4	14.0	32.1	32.1	40.2	40.2		30.1	7.9	26.3	14.0
β-Elemene					1.6			3.0			
β-Pinene	27.7	38.2	27.0	27.0	12.5	12.5		15.0		7.0	38.2
τ-Cadinol							6.3				
Sabinene	2.7	0.8									

^a^*Piper tuberculatum* Jacq. var. *tuberculatum*.

**Table 22:** Chemical compositions (%) of leaf essential oils from miscellaneous *Piper* species, part 1.

Compounds	*P. acre* Blume [35]	*P. acutifolium* Ruiz & Pav. [32]	*P. acutilimbum* C. DC. [90]	*P. aequale* Vahl [32]	*P. aleyreanum* C. DC. [32]	*P. amplum* Kunth [32]	*P. augustum* Rudge [32]	*P. barbatum* Kunth [91]	*P. bellidifolium* Yunck. [90]	*P. bisasperatum* Trel. [32]	*P. bredemeyeri* J. Jacq. [32]	*P. cachimboense* Yunck. [78]	*P. caldense* C. DC. [32]	*P. callosum* Ruiz & Pav.^a^ [44]	*P. caninum* Blume [92]	*P. durilignum* C. DC. [90]	*P. divaricatum* G. Mey.^b^ [32]	*P. diospyrifolium* Kunth [32]	*P. demeraranum* (Miq.) C. DC. [93]
(*E*)-Nerolidol	22.7								20.3						3.9	6.2		18.2	
*trans*-Sesquisabinene hydrate								8.2–20.9											
(*E*)-β-Ocimene		8.1																5.8	
*cis*-Eudesmα-6,11-diene																		21.1	
(*Z*)-β-Ocimene						10.5									3.4				
1-*epi*-Cubenol			5.6																
1,8-Cineole														3.7			8.9–9.6		
2-Undecanone															2.0				
*allo*-Aromadendrene		6.0																	
Apiole												5.7							
Aromadendrene									13.3										
Benzyl benzoate	3.5																		
Bicyclogermacrene					9.2					14.1									8.8
Caryophyllene oxide					11.5													7.7	
Cembratrienol 1							25.4												
Cembratrienol 2							8.6												
Cembrene							11.7												
Dillapiole		5.9																	
Elemol								7.2–10.2											
τ-Cadinol																5.2			
Germacrene A										13.2	13.2								
Germacrene B			6.9															6.2–6.7	
Germacrene D						5.5				9.5	21.7	6.3–27.6			4.9	11.1	6.3–6.5		5.2
Globulol																		6.6	
Hisenol									5.7										
Humulene epoxide I																		6.9	
Limonene				6.7		8.6	13.0	2.8–7.0								10.7		6.7–8.5	19.3
Linalool							10.3								7.0	5.1	23.4–29.7		
Longifolene									5.4										
Methyeugenol														7.6					
Sabinene	19.5			18.4															
Safrole														53.8	17.1				
Selin-11-en-4α-ol																		17.7	
Spathulenol					6.7							4.7						25.4	
Thujopsan-2β-ol													7.4						
α-Cadinene		6.7																	
α-Cadinol													19.0						
α-Copaene		6.1							10.9									5.4–47.7	
α-Humulene																		5.7	
α-Muurolol			6.4										9.0						
α-Phellandrene					8.6		14.7	9.8–43.16											
α-Pinene				12.6–39.3	7.0	18.1	6.0–10.5							12.2	4.0		9.0–18.8	6.7	
β-Caryophyllene		7.9			18.6	8.8	13.5				24.2	4.7–7.5			6.7	9.1		7.4–16.8	6.0
β-Elemene					16.3		12.3			46.4	34.0				2.1				33.1
β-Eudesmol	7.5							3.5–4.6											
β-Longipinene			6.2																
β-Phellandrene							5.6												
β-Pinene				15.6	14.4									7.7	8.9	5.0	19.9–25.3		6.7
γ-Amorphene												6.6–6.9							
γ-Eudesmol			7.5																
γ-Gurjunene																		6.9	
γ-Muurolene												4.0						10.6	
δ-Cadinene	12.4				6.2							6.2–9.2							
δ-Elemene				19.0	8.2														
β-Selinene																			5.0

^a^*Piper callosum leaves’ EO* can also be characterized by safrole and propiopiperone [49]. ^b^ *Piper divaricatum* has shown two phenylpropanoid chemotypes: a eugenol/methyleugenol CT and a safrole CT [32].

**Table 23:** Chemical compositions (%) of leaf essential oils from miscellaneous *Piper* species, part 2.

Compounds	*P. darienense* C. DC. [32]	*P. crassinervium* Kunth [32]	*P. curtispicum* C. DC. [32]	*P. corrugatum* Kuntze [32]	*P. corcovadensis* (Miq.) C. DC. [32]	*P. angustifolium* Lam. [32]	*P. consanguineum* (Kunth) Steud. [90]	*P. claussenianum* (Miq.) C. DC. [32,94]	*P. carpunya* Ruiz & Pav. [32,95]	*P. carniconnectivum* C. DC. Brazil [32]	*P. lanceifolium* Kunth^a^ [79,96]	*P. laosanum* C. DC. Vietnam [35]	*P. jacquemontianum* Kunth [48]	*P. ilheusense* Yunck. [32]	*P. humaytanum* Yunck. [32]	*P. hoffmanseggianum* Schult. [74]	*P. goesii* Yunck. [74]	*P. glabratum* Kunth [97]
(*E*)-Nerolidol		8.2		12.8		5.8	6.2	24.3–83.3					4.6					5.3
β-Caryophyllene		7.8–8.1			6.3			0.9–1.9			5.1–5.8			11.8		10.1	13.3	14.6
(*E*)-β-Farnesene	63.7																	
(*E*,*E*)-α-Farnesene																7.2	14.1	
(*Z*)-β-Ocimene																	1.1	
1,8-Cineole				5.9					13.0									
10-*epi*-γ-Eudesmol																16.0		
4-Butyl-1,2-methylenedioxybenzene					30.6													
Apiole											9.8–11.7							
Bicyclogermacrene		5.1–9.2							6.7									
Camphene	3.4																	
Carvotacetone acetate		8.2	8.2															
Caryophyllene oxide						13.1				21.3	3.4–5.9				16.6			
Dillapiole											9.8–74.6							
Elemicin											16.4–24.4							
*epi*-α-Selinene		5.0																
Germacrene B														7.2				
Germacrene D		9.2–14.0									10.7–42.8					4.95	28.87	
Gleenol														7.5				
Globulol																4.59		
Guiaol		5.5–5.8																
Hinesol																19.3		
Isocaryophyllene																		5.2
Limonene	6.3	26.6	26.6										12.2					
Linalool				4.2				2.1–53.5	5.65				14.5					
Longiborneol																		12.0
Patchouli alcohol														11.1				
*p*-Cymene	3.3	2.2		8.6					10.9				7.4					
Piperitone		2.6							51.0									
Sabinene												6.0						
Safrole									14.9									
Sesquicineole															5.0			
Spathulenol		9.8				23.8			9.8			5.1			6.3			
Terpinolene					17.4													
α- Humulene								2.5			2.6						3.3	
α-Cadinol												4.9						
α-Cubebene											4.3							
α-Curcumene												12.0						
α-Eudesmol																8.3		
α-Muurolol							5.0											
α-Phellandrene				4.7									13.8					
α-Pinene		10.0–11.5		12.2	5.9	5.9					3.6 -13.7		9.6					9.7
α-Selinene											7.8							
α-Terpinene		7.8							12.1									
α-Terpineol									4.7									
α-Terpinyl acetate		4.3																
β-Elemene											2.5							
β-Eudesmol		10.1														8.1		
β-Oplopenone															6.0			
β-Phellandrene				8.2														
β-Pinene		11.6–15.2		26.6						6.3	5.4–15.8		10.1					13.0
β-Selinene								0.7			4.8–7.8				15.8			
γ-Cadinene							11.3							6.9				
γ-Elemene								0.5										
γ-Eudesmol							18.0									8.5		
γ-Muurolene								3.2			4.0							
γ-Terpinene		2.9									6.9							
δ-Cadiene											6.1						3.8	6.3
δ-Elemene																3.3	5.9	

^a^*Piper lanceifolium* Kunth is a very polymorphic species, which is reflected in the high chemical diversity of the EO, that in certain cases can be dominated by phenylpropanoids, and in others be completely devoid of the same compounds [79,96].

**Table 24:** Chemical compositions (%) of leaf essential oils from miscellaneous *Piper* species, part 3.

Compounds	*P. fulvescens* C. DC. [39]	*P. friedrichsthalii* C. DC. [48]	*P. fimbriulatum* C. DC. [32,48]	*P. obliquum* Ruiz & Pav. [48]	*P. multiplinervium* C. DC. [48]	*P. mollicomum* Kunth^a^ [74,98]	*P. mosenii* C. DC. [78,99]	*P. longispicum* C. DC. [32,48]	*P. leptorum* Kunth [32]	*P. lucaeanum* Kunth [32]	*P. madeiranum* Yunck. [32]	*P. vicosanum* Yunck. [100]	*P. variabile* C. DC. [32]	*P. tectoniaefolium* (Kunth) Kunth ex C. DC. [32]	*P. solmsianum* C. DC. [32,78]	*P. reticulatum* L. [32,48]	*P. glabrescens* (Miq.) C.DC. [32]	*P. trigonum* C. DC. [48]	*P. gaudichaudianum* (Kunth) Kunth ex Steud. [77]	*P. imperiale* (Miq.) C. DC. [32]	*P. grande* Vahl [48]
(*E*)-Anethole	26.4																				
(*E*)-Isoelemecin															53.5						
(*E*)-Nerolidol					5.5	7.5–23.2													13.7		
β-Caryophyllene	3.7	4.3	11.3	27.6			8.6–16.8	45.2	45.2	5.0	11.2				9.2			7.1	23.4	25.5	
(*Z*)-β-Farnesene						5.7															
1,10-di-*epi*-Cubenol											7.0										
1,8-Cineole												10.4–15.0									
Selin-11-en-4α-ol		12.8																			
2-Tridecanone						4.3															
Aromadendrene	4.2																				
Asaricin															39.2						
Bicyclogermacrene							7.4								32.5					19.7	
Camphene						25.3							16.6								
Camphor						39.9							28.4								
Caryophyllene oxide				8.3		3.1	9.8 -12.1	5.5						10.9							
Dillapiole																			2.8	6.7	
*epi*-α-Bisabolol											5.4										
Germacrene A																				8.5	
Germacrene D		9.6	12.8–32.9	3.9		3.7		3.3										19.7		5.5	
Germacrene D-4-ol											11.1										
Globulol																			6.1		
Guaiol													6.3								
Humulene epoxide II						2.3	6.3														
Ishawarane	12.1																				
Limonene					11.4		5.1					9.1–45.5	13.9				56.6				
Linalool			5.3		16.5																
Linalyl acetate			5.3																		
Myrcene															26.1						
*p*-Cymene					9.4								6.3								43.9
Spathulenol	5.4	4.3		10.6				3.8							5.2–7.5	6.1			11.1		
Terpinolene												10.1									
Viridiflorol							5.8														
α-Alaskene												13.4									
α-Bisabolol											7.1										
α-Bulnesene																				10.8	
α-Cadinol																		5.8			
α-Copaene		3.3		5.6				3.4	3.4									6.0			
α-Guaiene																				7.6	
α-Humulene						4.2	11.3														
α-Phellandrene					11.8																
α-Pinene			10.2		7.1	6.3				30.0		6.1–7.2		12.9	7.8–22.7		26.0				6.3
α-Selinene		12.0													5.5	15.5					
α-Zingiberene										30.4											
β-Bisabolene				4.5						8.9											
β-Elemene																16.1		8.4		5.2	
β-Pinene					7.9		5.8							8.8							14.5
β-Selinene		7.9														19.0					
β-Sesquiphellandrene										11.1											
γ-Elemene												14.2									
γ-Muurulene																		3.7			
γ-Terpinene																					8.0
δ-3-Carene															23.3–66.9						
δ-Cadinene		4.2				2.3												7.2			
δ-Elemene			9.4																		

^a^ Krinski et al. [78] distinguisah two chemotypes from Brazilian plants. CT1 has is characterized by bicyclogermacrene (20.4%), asaricin (7.2%), *allo*-aromadendrene (6.4%), and (*E*)-β-ocimene (4.4%), while CT2 is characterized by spathulenol (21.1%), terpinolene (11.3%), and δ-cadinene (9.1%).

**Table 25:** Chemical compositions (%) of leaf essential oils from miscellaneous *Piper* species, part 4.

Compounds	*P. aereum* Trel.	*P. duckei* C. DC. [93]	*P. friedrichsthalii* C.DC.	*P. grande* Vahl	*P. heterophyllum* Ruiz & Pav.	*P. hostmannianum* (Miq.) C.DC.	*P. jacquemontianum* Kunth	*P. malacophyllum* (C.Presl) C.DC.	P. *mollicomum* (Kunth) Kunth ex Steud.	*P. nemorense* C.DC.	*P. ovatum* Vahl	*P. peltatum* L.	*P. pseudolindenii* C.DC.	*P. regnelli* (Miq.) C.DC. and *P. regnellii* (Miq.) C. DC. var. *regnellii*	*P. rivinoides* Kunth	*P. trigonum* C.DC.
*trans*-Calamenene												5.4				
(*E*)-Nerolidol							8.0	9.1	9.6	5.5				8.4		
*trans-p*-Menth-2-en-1-ol			7.0													
(*E*)-β-Ocimene					6.5				12.1–14.0							
*cis-p*-Menth-2-en-1-ol			5.1													
(*Z*)-α-Bisabolene															10.9	
(*Z*)-β-Ocimene									12.1–14.1							
1,8-Cineole		5.8			39.0											
Asaricin					8.8	27.4										
Bicyclogermacrene		5.2						5.4						9.7	11.8	
Camphene								20.8–30.8								
Camphor								32.8								
Caryophyllene oxide												22.9				
Dehydroaromadendrane															7.8	
Dillapiole						7.7										
*epi*-Cubebol											10.7					
Germacrene B									13.4				5.4		6.7	
Germacrene D		14.7	9.6			6.8			10.8		10.3		9.0	45.6–51.4		19.7
Guaiol	41.2															
Limonene							12.2			6.3–11.4						
Linalool							14.5–69.4			16.5				15.9		
Myrcene														15.5–52.6		
Myristicin						20.3										
Myrtenic acid									7.5							
*o*-Cymene								6.2								
*p*-Cymene				43.9			7.4			9.0						
Piperitone						5.6										
Selin-11-en-4α-ol			12.8													
Spathulenol												9.0		7.8	5.1	
Terpinolene								8.2								
α-Bisabolol									9.9							
α-Cadinol	9.2															5.8
α-Chamigrene														8.9–11.3		
α-Copaene										5.7		5.2				6.0
α-Humulene													7.0		10.0	
α-Muurolol	5.8															
α-Phellandrene							13.8			8.8–11.8						
α-Pinene			13.4	6.3	9.3		9.6	5.0		7.1	23.1				32.9–73.2	
α-Selinene			12.0													
α-Terpinene								13.9								
β-Aromadendrene														8.3		
β-Bourbonene										14.0						
β-Caryophyllene	6.6	27.1								5.6	5.3		11.8	7.2–9.5	6.6–7.6	7.1
β-Copaene										15.0						
β-Elemene													15.0			8.4
β-Phellandrene			5.2													
β-Pinene				14.5	6.2		10.1			7.9	14.2		6.7	13.3	5.2–24.7	
β-Selinene			7.9					5.9								
γ-Elemene										6.8						
γ-Eudesmol		17.9														
γ-Terpinene				8.0				21.5								
δ-Cadinene	7.3															7.2

Taken from [32] unless otherwise stated.

**Table 26:** Chemical compositions (%) of aerial parts essential oils from miscellaneous *Piper* species, part 1.

Compounds	*P. aequale* Vahl	*P. aleyreanum* C. DC. [101]	*P. anonifolium* (Kunth) Steud.	*P. artanthe* C.DC.	*P. austrosinense* Y.Q. Tseng [34]	*P. barbatum* Kunth	*P. bisasperatum* Trel. [102]	*P. bogotense* C.DC.	*P. boehmeriifolium* (Miq.) Wall. ex C. DC. [34]	*P. brachypodon* (Benth.) C. DC.	*P. bredemeyeri* J.Jacq. [75]	*P. cryptopodon* C. DC.	*P. dactylostigmum* Yunck.	*P. demeraranum* (Miq.) C.DC.	*P. divaricatum* G.Mey. [75]	*P. flaviflorum* C. DC. [34]	*P. hainanense* Hemsl. [34]
(*E*)-Asarone						14.1											
*trans*-Calamene		6.4															
(*E*)-Nerolidol									10.4			6.6				48.4	
*trans*-Sabinene hydrate								14.2									
(*Z*)-Asarone					4.7												
(*Z*)-Isoelemicin					6.2												
*cis*-β-Guaiene		29.3															
Cadalene					4.9												
1,8-Cineole															18.0		
Propiopiperone																	5.6
2′-Methoxy-4′,5′-methylenedioxypropiophenone						29.5											
Eugenol																	54.3
9-*epi*-β-Caryophyllene										5.8							
Apiole				14.5	4.2	8.0											
Asaricin					4.4												
Asarone					3.2												
Bicyclogermacrene	5.5	9.2								8.1		8.3–23.3					
Bulnesol																3.6	
Camphene		4,1–5.2															
Caryophyllene oxide		11.5			5.8					10.8			6.0				51.8
Crocatone						10.9											
Cubebol	7.2			6.3													
*epi*-Cubebol				8.9													
*epi*-α-Bisabolol												26.3					
Germacrene B												10.1–26.8					
Germacrene D		6.9	9.6							5.9	4.0	7.5–17.9					
Guaiac alcohol																4.64	
Palmitic acid					3.5												
Ishwarane			19.1														
Limonene			5.9–8.5					5.3			4.0			20.2–40.3			
Linalool															15.0		
Methyl (*E*)-cinnamate									40.7								
Myristicin				6.4													
*p*-Cymene		3.8										6.3					
Phytol					4.5												
Sabinene														12.9–22.7			
Selin-11-en-4α-ol			20.0														
Spathulenol		5.2–6.7			6.3					5.7		6.9–8.4				4.6	53.8
τ-Cadinol					6.8												
α-Bisabolol									17.6								
α-Bulnesene		5.2															
α-Cadinol												9.5	21.7				
α-Cubebene												5.1–6.7					
α-Eudesmol			33.5														
α-Phellandrene								13.7							6.0		
α-Pinene	12.6		7.3–53.1					8.7			20.3	7.5		7.3	11.0		
α-Selinene			11.9										8.0				
β-Atlantol	5.9																
β-Caryophyllene		6.2–6.6	2.5–6.3	10.2						20.2	6.3	18.1–34.6	8.9		8.0	5.4	
β-Elemene		8.8–16.3									4.0						
β-Pinene	15.6	5.1–9.0	17.2–22.9								32.3	6.0		7.7–14.4	5.0		
β-Selinene			12.7										9.0				
γ-Muurolene					4.3								5.9				
δ-Cadinene					8.1				3.8								
δ-Cadinol					8.1 -23,1												
δ-Elemene	19.0	8.2		11.7													
τ-Muurolol													7.5				

Taken from [32] unless otherwise stated.

**Table 27:** Chemical compositions (%) of aerial parts essential oils from miscellaneous *Piper* species, part 2.

Compounds	*P. hancei* Maxim. [34]	*P. krukaffii* Yunck.	*P. laetispicum* C. DC. [34]	*P. manausense* Yunck.	*P. mikanianum* (Kunth) Steud. [103]	*P. obliquum* Ruiz & Pav. ^a^ [36]	*P. vitaceum* Yunck.	*P. wallichii* [34]	*P. xylosteoides* (Miq.) Hand.-Mazz.	*P. puberulum* (Benth.) Maxim. [34]	*P. senporeiense* Yamam. [34]	*P. septuplinervium* (Miq.) C. DC. [104]	*P. sarmentosum* Roxb. [34]	*P. brachypodon* Yunck var *hirsuticaule*	*P. obrutum* Trel. & Yunck.	*P. plurinervosum* Yunck.	*P. regnellii* (Miq.) C.DC.	*P. renitens* (Miq.) Yunck.
(*E*)-Asarone					11.6													
(*E*)-Nerolidol							20.6		8.2–8.5						5.8	6.4	13.7	
*trans*-β-Guaiene									7.8									
(*Z*)-Asarone										11.5	5.1							
(*Z*)-Isoelemicin	3.5				21.5			4.9		10.1	4.4							
1,8-Cineole																31.6		
Propiopiperone	12.2							5.8		23.4	21.1							
Apiole		25.3–34.1			64.9					2.7								
Aromadendrene				5.0														
Asaricin	65.9									2.6			54.5					
Asarone										28.5	3.6							
Bicyclogermacrene				7.8–41.0	5.3–14.3				7.2					6.2				6.6
Camphene																		5.6
Caryophyllene oxide							5.2									5.7		
Elemicin	4.1									2.6			3.2					
*epi*-Cubebol												9.0						
Eudesm-7(11)-en-4-ol																		9.3
Germacrene B					7.8				10.6									
Germacrene D				3.5–6.1										16.7				13.8
Germacrene D-4-ol												5.6						
Gleenol				6.8–9.4														
Globulol			3.8	9.4													6.1	
Guaiol																6.2		13.9
Palmitic acid	2.5												8.2					
Isocaryophyllene					6.8								2.4					
Isospathulenol								3.1										
Limonene					14.8		33.2		5.1									
Linalool															15.8			
Myrcene					5.6				31.0									
Myristicin		26.7–40.6																
*p*-Cymene						2.6	12.8		12.4									
Phytol								7.4					2.9					
Safrole					44.1–82.0	45.9			47.8–84.1									
Spathulenol	7.8			15.0				18.8	12.3		7.7						11.1	
Terpinolene						11.5												
Valencene					8.0													
Viridiflorol												7.9						
Zingiberene									9.3									
α-Cadinol					5.1						4.4					8.5		
α-Eudesmol	6.9							5.8										
α-Guaiene														5.9				
α-Gurjunene			4.7															
α-Humulene			9.6												6.4			
α-Muurolol				7.6														
α-Pinene				5.2–9.1	6.5–19.4				6.0–15.3									12.5
α-Selinene	7.1		19.1															
α-Terpinene						6.2			11.3									
α-Thujene					6.0				7.9									
β-Caryophyllene			8.8	5.9–8.5	10.5				7.0			5.0		9.8		6.6	23.4	6.9
β-Copaen-4α-ol									9.4									
β-Elemene		1.7–8.2	4.6											6.4	7.6			
β-Eudesmol	6.8							6.0										
β-Phellandrene									22.6									
β-Pinene				4.7–9.2		2.0												12.4
β-Selinene	7.99		23.8															
β-Vetivone					33.5													
γ-Terpinene						17.1			26.1									
δ-Cadinene				5.8–7.0							4.7	10.9						
δ-Elemene									6.6									
τ-Muurolol		0.2–5.7																

Taken from [32] unless otherwise stated. ^a^ The essential oil from Panama is rich in β-caryophyllene (27.6%) with a pattern dominated by sesquiterpenes (78% approximately) [105].

**Table 28:** Antibacterial and antifungal activities of *P. nigrum* L. (Black pepper).

Extract Type	Bacteria and Fungi	Main Results	References
Seeds: acetone and dichloromethane extracts	Gram positive (*Staphylococcus aureus*, *Bacillus cereus, Staphylococcus faecalis);* Gram negative *(Pseudomonas aeruginosa, Salmonella typhi, Escherichia coli)*	MIC of 50–500 ppm showed excellent inhibition	[254]
EO	*Vibrio cholerae*, *Staphylococcus albus*, *Clostridium diphthereae*, *Shigella dysenteriae*, *Streptomyces faecalis*, *Bacillus* spp., *Pseudomonas* spp., *Aspergillus parasiticus*	EO inhibited bacterial and fungal growth	[255]
Encapsulated EO	*Staphylococcus aureus* and *Escherichia coli*	Antibacterial activity was four times greater with encapsulation when compared to free EO	[256]
Leaf: ethanol & methanol extract	*Staphylococcus aureus, Escherichia coli, Salmonella typhimurium*, and *Pseudomonas aeruginosa* and fungi (*Aspergillus* spp. and *Candida albicans*)	Minimum bactericidal and fungicidal concentrations were between 12.5–50.0 mg/mL for all tested strains	[257]
EO	Four strains of *E. coli* isolated from pork	MIC was found to be 1.0 μL/mL. The diameter of inhibition zone values was with range from 17.12 to 26.13 mm diameter. EO treatment also caused the physical and morphological alterations in the cell wall and membrane of *Escherichia coli*.	[262]
Seeds: chloroform extract	*Escherichia coli* and *Staphylococcus aureus*	The extract increased pyruvic acid concentration in bacterial solutions and reduced the ATP level in bacterial cells. The extract destroyed the permeability of the cell membrane, which consequently caused metabolic dysfunction, inhibited energy synthesis, and triggered cell death.	[263]
Callus, shoots, and seeds: ethanol, hexane, and chloroform, extract	*Bacillus subtilis, Bacillus cereus, Staphylococcus aureus,* and *Candida albicans*	The zone of inhibition for these tested strains ranged from 16–26 mm in diameter.	[264]
Seeds: petroleum ether extract	*Listeria monocytogenes* and *Salmonella typhimurium*	The MICs of extract against *Listeria monocytogenes* and *Salmonella typhimurium* were 0.625 and 1.25 mg/mL, respectively. Results also showed that the extract could destroy cell wall integrity, alter the permeability of the cell membrane, and inhibit the activity of intracellular enzymes, which would make the extract bactericidal.	[265]

EO essential oil; MIC minimum inhibitory concentration.

**Table 29:** *Piper* plants with reported activity on parasite of medical importance.

*Piper* Species	Tested Product	Parasite Target	References
*Piper aduncum* L.	Extract	*Leishmania amazonensis*	[271]
	Essential oil	*Leishmania braziliensis*	[275]
	Essential oil	*Trypanosoma cruzi*	[45]
	Essential oil	*Plasmodium falciparum, Trypanosoma cruzi, Trypanosoma brucei, Leishmania amazonensis, Leishmania infantum*	[41]
*Piper aduncum* var. *ossanum* C.DC.	Essential oil	*Plasmodium falciparum, Trypanosoma cruzi, Trypanosoma brucei Leishmania amazonensis, Leishmania infantum*	[276]
*Piper amalago* L.	Extract	*Leishmania amazonensis*	[277]
*Piper auritum* Kunth	Essential oil	*L. major*, *L. mexicana*, *Leishmania braziliensis* and *L. donovani*	[76]
	Extract	*Leishmania* spp.	[278]
*Piper betle* L.	Extract	*G. lamblia*	[269]
	Extract	*L. donovani*	[279]
	Extract	*L. donovani*	[280]
	Extract	*Brugia malayi*	[281]
	Extract	*P. berghei*	[282]
	Extract and fractions	*L. donovani*	[283]
	Extract	*Toxoplasma gondii*	[284]
	Extract	*Neospora caninum*	[270]
*Piper capense* L.f.	Extract	*Plasmodium falciparum*	[268]
*Piper chaba* Hunt	Extract	*Schistosoma mansoni*	[285]
	Extract	*Plasmodium falciparum*	[286]
*Piper claussenianum* (Miq.) C. DC.	Essential oil	*Leishmania amazonensis*	[94]
*Piper cubeba* L.f.	Essential oil	*Trypanosoma cruzi*	[287]
*Piper cumanense* Kunth	Extract	*Plasmodium falciparum*	[170]
*Piper demeraranum* (Miq.) C. DC.	Essential oil	*Leishmania amazonensis* and *L. guyanensis*	[93]
*Piper dennisii* Trel	Extract	*Leishmania amazonensis*	[112]
*Piper duckei* C. DC.	Essential oil	*Leishmania amazonensis* and *L. guyanensis*	[93]
*Piper friedrichsthalii* C. DC.	Extract	*P. berghei*	[288]
*Piper hispidum* Sw.	Extract	*Plasmodium falciparum*	[289]
	Extract	*Leishmania amazonensis*	[193]
	Essential oil	*Leishmania amazonensis*	[274]
*P. heptaphyllum* (Aubl.) Marchand	Essential oil	*Leishmania amazonensis*	[274]
*Piper jericoense* Trel. & Yunck.	Extract and fractions	*Trypanosoma cruzi*	[290]
	Extract and fractions	*Trypanosoma cruzi*	[291]
*Piper laevicarpum* Yunck.	Extract	*Trypanosoma cruzi*	[292]
*Piper longum* L.	Ayurvedic herbal medicine preparation	*Giardia lamblia*	[293]
	Extract	*Giardia lamblia*	[294]
	Extract	*Blastocystis hominis*	[269]
	Extract	*Leishmania donovani*	[295]
	Extract	*Echinococcus granulosus*	[296]
*Piper loretoanum* Trel.	Extract	*Leishmania amazonensis*	[237]
*Piper malacophyllum* (C. Presl) C. DC.	Essential oil	*Trypanosoma cruzi* and *Leishmania infantum*	[297]
*Piper nigrum* L.	Fractions	*Leishmania donovani*	[298]
	Extract	*Plasmodium falciparum*	[272]
*Piper sanguineispicum* Trel.	Extract	*Leishmania amazonensis*	[237]
*Piper strigosum* Trel.	Extract	*Leishmania amazonensis*	[193]
*Piper tuberculatum* Jacq.	Extract	*Rhipicephalus* (*Boophilus*) *microplus*	[299]
	Extract	*Haemonchus contortus* and *Strongyloides venezuelensis*	[300]
	Essential oil	*Leishmania braziliensis*, *Leishmania infantum* and *Trypanosoma cruzi*	[88]
*Piper umbellatum* L.	Extract	*Onchocerca ochengi* and *Loa loa*	[149]

**Table 30:** Antiproliferative properties of different *Piper* extracts.

*Piper* Species Extract	Design/Model	Key Effects	References
***Piper longum*L.** Hexane, Benzene, Acetone, Ethyl Acetate, Ethyl Alcohol, Chloroform, And Aqueous Extracts	**in-vitro studies** Du-145, A549, Thp-1, Igr-Ovi-1 Ovary and MCF-7 cells **in-vivo studies** Metal-induced Wistar rats	-growth inhibition for all extracts in THP-1 (76–90%) -growth inhibitory effect of hexane and benzene extracts (>80%) on all cell lines. -increased cell cycle inhibition (41, 63 and 43%, respectively) and sub-G1 DNA fraction population for hexane, benzene and acetone extracts in A549 cell line. -protective effect of aqueous, chloroform and ethyl alcohol extracts on liver (65, 71 and 64%), respectively against peroxidative damage.	[309]
***Piper longum*L.** Ethanol Extract	**in-vitro studies** G-361, HT-29 And HCT116, OVCAR-3, BXPC-3 cells **in-vivo studies** Immunocompromised mice	-selectively induced cell death in cancer cells-colon (HCT116), pancreatic (BxPC-3), leukemia (T cell)-but was not effective for normal colon epithelial cells. -the growth of colon cancer tumors in ethanolic extract treated ımmunocompromised mice group of animal models was suppressed without any toxic effect.	[310]
***Piper umbellatum*L.** Dichloromethane Extract	**in-vitro studies** UACC-62, -U251, MCF-7, NCI-H460, PC-3, NCI-ADR/RES, and OVCAR-3 cells **in-vivo studies** Carrageenan-induced paw edema and peritonitis models Balb/C mice	-total growth inhibition in several different human tumor cell lines. -the sizes of Ehrlich solid tumor Balb/C mice were reduced 38.7 and 52.2% in 200 and 400 mg/kg extract treatment groups, respectively, without toxicity.	[311]
***Piper tuberculatum*Jacq.** Crude Extract and Piplartine	**in-vitro studies** SF-295 and HCT-8 cells **in-vivo studies** Bal/C (Nu/Nu) mice	-cytotoxic activity in SF-295 cells (10 μg/mL) and in HCT-8 cell line (4.3 μg/mL). -inhibition of cell proliferation (24.6–54.8%) in Ehrlich solid tumor Balb/C (Nu/Nu) mice for (100–200 mg/kg/day) crude extract.	[10]
***Piper nigrum*L.** Ethanolic Extract	**in-vitro studies** MCF-7 and HT-29 cells **in-vivo studies** Ehrlich carcinoma mice	-EC50 in MCF-7: 27.1±2.0 µg/mL and in HT-29: 80.5±6.6 µg/mL. -In Ehrlich carcinoma model of mice 60% decrease of tumor growth and %76 increase of survival time -increased rate of apoptosis. -increasing expression levels of Bax and p53 protein inhibition of Bcl-xL and cyclin A expressions.	[312]
***Piper nigrum*L.** Supercritical Fluid Extraction SFE200 Extract/Piperine Higher Piperine Content	**in-vitro studies** MCF-7 cells **in-vivo studies** Ehrlich carcinoma mice	-higher cytotoxic effect in MCF-7 cells than conventional extract. -more significant tumor growth inhibition and increased survival time as compared to conventional extract treatment group of the mice. -decreased cell number founding at S phase.	[313]
***Piper nigrum*L.** Piperine-free *P. nigrum* Extract (PFPE)	**in-vitro studies** Several cell line including MCF-7 **in-vivo studies** N-nitroso-N-methylurea–induced mammary tumorigenesis sprague-dawley rats	-higher cytotoxic effect of PFPE on MCF-7 breast cancer cell line (IC50:7.45 µg/mL), together with better selectivity. -PFPE induced apoptosis in MCF-7 cells in dose dependent manner. -upregulation of p53 and cyt C and downregulation of topoisomerase II. -100 mg/kg PFPE treatment result in decrease of tumor growth in rats with mammary tumorigenesis.	[314]

**Table 31:** Anti-inflammatory properties of different *Piper* extracts.

*Piper* Species Extract	Design/Model	Key Effects	References
***Piper betle* L.** Hydroalcoholic Extract	**in-vivo studies** Carrageenan-induced paw edema (Wistar rats) Cotton pellet-induced granuloma (Albino mice)	-significant analgesic property in both animal groups. -decreasing in the pain by both central and peripheral mechanisms. -inhibition of the growth of paw at 50, 100, 200 mg/kg treatment groups in dose dependent manner. -reduction in the dry weights of granuloma.	[349]
***Piper crocatum* Ruiz & Pav.** 96% Ethanol Extract	**in-vitro studies** LPS-induced RAW 264.7 Cells	-cytotoxic effect of extract in 150 μg/mL or higher concentration treatment group. -significant decrease in TNF-α level at 50 μg/mL treated cell group. -reduction in IL-1β level at 10, 50 and 75 ug/mL treatment groups. -significant reduction in IL-6 and NO levels at 50 μg/mL treatment group.	[348]
***Piper nigrum* L.** Ethanol, Hexane Extracts	**in-vivo studies** Carrageenan-induced Paw edema model Swiss albino mice	-reduction of edema growth in 5–10 mg/kg of hexane extract treatment groups. -good inhibition profile for edema growth at 10 mg/kg of ethanol extract treatment group.	[346]
***Piper nigrum* L.** Ethanol Extract	**in-vivo studies** OVA-induced allergic asthma model BALB/c mice	-decreasing in number of inflammation related cells. -regulation of balance of T cells responses by decreasing IL-1β, IL-4, IL-17A, and TNF-α cytokine levels.	[347]
***Piper umbellatum* L.** Standardized Dichloromethane Extract	**in-vivo studies** Carrageenan-induced paw edema and peritonitis in Balb/C mice	-inhibitory effect on paw edema at 48 h without toxicity.	[311]
**Piperaceae species** Ethanolic-Crude Extract	**in-vitro studies** LPS-induced PBMC	-differential inhibitory effect on proinflammatory cytokines.	[350]

**Table 32:** Neuropharmacological activities of different *Piper* extracts.

*Piper* Species Extract	Design/Model	Key Effects	References
***Piper betle* L.** Aqueous and Ethanol Extracts	**in-vitro studies** SH-SY5Y cells	-inhibitory activity for both acetyl- and butyrylcholinesterase. -no effect of ethanolic extract on the mitochondrial function or the membrane integrity between 7.8–1000 µg/mL concentrations. -cell viability reduction about 20% by 125 µg/mL of aqueous extract.	[363]
***Piper nigrum* L.** Methanol Extract	**in-vivo studies** AlCl3-induced AD model Sprague-Dawley rats	-marked improvement in the biochemical parameters (Ach, CRP, NF-ĸB, MCP-1) in brain of AD rats.	[374]
***Piper nigrum* L.** Methanol Extract	**in-vivo studies** Aβ (1-42)-induced AD model Wistar rats	-significantly improved memory performance and exhibited remarkable antioxidant potential.	[375]
***Piper nigrum* L.** Methanol Extract	**in-vivo studies** Aβ (1-42)-induced AD model Wistar rats	-significantly increased the swimming time. -increase in the number of open-arm entries.	[365]
***Piper sarmentosum*Roxb.** Hexane, Dichloromethane, Ethyl acetate, and Methanol Extracts	**in-vitro studies** Aβ-induced BV-2 cells Aβ-induced microglia-mediated neurotoxicity in SH-SY5Y cells	-reduction in the secretion levels of Aβ-induced pro-inflammatory cytokines (IL-1β and TNF-α) by downregulating the mRNA expressions of pro-inflammatory cytokines in BV-2 cells with ethyl acetate and methanol extract. -protection of SH-SY5Y cells against microglia-mediated neurotoxicity through downregulation of phosphorylated tau proteins with ethyl acetate and methanol extract.	[364]
***Piper sarmentosum*Roxb.** Ethanol Extract	**in-vivo studies** CUMS-treated ICR mice	-reduced immobility time in the forced swimming test and tail suspension test. -no influence on the locomotor activity in the open field test -up-regulation of BDNF protein levels. -increased CREB and ERK phosphorylation levels in the hippocampus on CUMS rats.	[373]

**Table 33:** Neuropharmacological activities of piperine as one of the active compounds of *Piper* plants.

Design/Model	Key Effects	References
**in-vivo studies** 6-OHDA-induced PD Model of Wistar Rats	-reduction of neuronal cell apoptosis at a remarkable rate through inhibition of poly (ADP-ribose) polymerase activation and pro-apoptotic Bax levels as well as through the elevation of Bcl-2 levels. -decreasing in cytochrome-c release from mitochondria. -reduction in caspase-3 and caspase-9 activation. -reduction in lipid peroxidation and stimulated glutathione levels in striatum of rats. -depletion of inflammatory markers, TNF-α and IL-1β in 6-OHDA-induced PD model of rats.	[366]
**in-vivo studies** CUMS-treated mice	-significant reduction of immobility time in both forced swim and tail suspension tests. -significant amelioration on behavioural deficits of CUMS-treated mice. -significantly increased BDNF protein expression in the hippocampus and frontal cortex of both naive and CUMS-treated mice.	[380]
**in-vivo studies** Corticosterone-induced Depression Model in ICR mice	-suppression of corticosterone induced-depression like behavior in mice. -significant decrease in sucrose consumption. -increasing BDNF expression.	[380]
**in-vivo studies** MPTP-induced PD Model in C57BL/6 Mice	-neuroprotective effects against MPTP-induced mouse model of PD. -decreasing MPTP-induced deficits in motor coordination and cognitive functioning. -prevention MPTP-induced decreases in the number of tyrosine hydroxylase-positive cells in the substantia nigra. -reduction the number of activated microglia. -inhibition of expression of cytokine IL-1β and oxidative stress. -maintaining the balance of Bcl-2/Bax.	[381]
**in-vitro studies** Rotenone-induced SK-N-SH and Primary Rat Cortical Neuron Cells **in-vivo studies** Rotenone-induced PD Model in C57BL mice	-increasing cell viability and restored mitochondrial functioning (in-vitro studies). -reduction of rotenone-induced motor deficits of PD mice models. -recovery the loss of dopaminergic neurons in the substantia nigra of PD mice models. -increasing in rate of autophagy by inhibiting mTORC1 and activation of PP2A in PD mice model.	[386]
**in-vivo studies** Rotenone-induced PD Model in Wistar Rats	-abrogation of apoptosis. -stimulation of autophagy likely mitigating neuronal injury in the rotenone-induced rat model of PD.	[387]
**in-vitro studies** LPS-induced BV2 Microglia	-inhibition in LPS-induced TNF-α, IL-6, IL-1β, and PGE 2 production. -inhibition of LPS-induced NF-κB and activation of Nrf2.	[388]
**in-vivo studies** ICV-STZ-induced AD Model in Wistar rats	-cognitive enhancing effect. -hippocampal malonaldehyde decrement and keeping redox balance.	[420]
**in-vivo studies** ICV- colchicine induced AD Model in Wistar rats	**Piperine-loaded oral microemulsion:** -associated with increased delivery to brain. -increased efficacy. -better therapeutic outcome.	[393]
**in-vivo studies** NMRI mice	**Piperine-loadedchitosan-sodium tripolyphosphate nanoparticles:** -associated with enhanced neuroprotection. -amelioration of the astrocytes activation.	[394]

**Table 34:** Clinical trials on patients with generalized anxiety disorder.

Design	Treatment	Patients (n)	Key Effects	References
**Clinical trial** randomized double-blind placebo-controlled	Kava Extract (3 X 100 mg)/day **Duration:** 4 Weeks	*n* = 58	-Hamilton-Anxiety-Scale (HAMA) Score significantly reduced in drug receiving group compared to placebo group. -No adverse effects.	[367]
**Clinical trial** randomized double-blind placebo-controlled	Kava Extract (3 X 110mg)/day **Duration:** 25 Weeks	*n* = 101	-Significant superiority of kava extract in Hamilton-Anxiety-Scale (HAMA) Score over the placebo starting from week 8. -Rare and evenly distributed adverse effects.	[368]
**Clinical trial** randomized double-blind placebo-controlled	Kava Extract (280 mg)/day **Duration:** 4 Weeks	*n* = 13	-Significantly improved baroreflex control of heart rare following treatment with placebo. -No effect on respiratory sinus arrhythmia.	[407]
**Clinical trial** randomized open study	Kava Extract (100 and 200 mg)/day **Duration:** 3 Months	*n* = 80	-In kava treatment groups anxiety climacteric score declined at 1.and 3. Months. -Depression reduced at month 3. -In control group no significant result.	[413]
**Clinical trial** double-blind crossover trial placebo-controlled	Kava Extract 5 tablets/day Containing 3.2 g extract (delivering 50 mg kavalactones) **Duration:** 1 month	*n* = 60	-Kava resulted in 11.4 points reduction on HAMA Score over placebo. -Antidepressant effect. -No serious adverse effect. -No clinical hepatotoxicity.	[416]
**Clinical trial** randomized double-blind placebo-controlled	Kava Extract1 or 2 tablet/day (120 or 240 mg) kavalactone **Duration:** 6 Weeks	*n* = 75	-Significant increase in female’s sexual drive in Kava group compared to placebo (assessed by ASEX). -Significant reduction of anxiety in Kava group compared to placebo (assessed by HAMA). -No significant differences across groups for liver function test. -No differences in withdraw or addiction.	[417,418]
